# Reactivity of an arsanyl-phosphagallene: decarbonylation of CO_2_ and COS to form phosphaketenes†‡

**DOI:** 10.1039/d5sc00295h

**Published:** 2025-03-17

**Authors:** Lilian S. Szych, Jonas Bresien, Lukas Fischer, Moritz J. Ernst, Jose M. Goicoechea

**Affiliations:** a Department of Chemistry, University of Oxford, Chemistry Research Laboratory 12 Mansfield Road Oxford OX1 3TA UK; b Institut für Chemie und Biochemie, Freie Universität Berlin Fabeckstraße 34/36 Berlin 14195 Germany; c Institut für Chemie, Universität Rostock Albert-Einstein-Straße 3a Rostock 18059 Germany; d Department of Chemistry, Indiana University Bloomington, 800 E. Kirkwood Ave. Bloomington IN 47405-7102 USA jgoicoec@iu.edu

## Abstract

The synthesis of an arsanyl-phosphagallene [H_2_CN(Dipp)]_2_AsP=Ga(NacNac) (NacNac = HC[C(Me)N(Dipp)]_2_; Dipp = diisopropylphenyl) and its reactivity towards heterocumulenes and ketones is described. Reactions with azides, carbodiimides, isocyanates and ketones give rise to heterocycles *via* cyclization reactions involving the Ga=P π-bond (with the Ga–P σ-bond remaining unperturbed in the final products). By contrast, reactions with CO_2_, CS_2_ and COS are more intriguing, revealing a reactivity profile in which the phosphorus atom can abstract carbon monoxide from the oxygen-containing heterocumulenes. These reactions result in the formation of gallium phosphaethynolate compounds. Such reactivity is enabled by the presence of a weakly Lewis basic arsanyl moiety which, in contrast to other related compounds featuring phosphanyl groups, is insufficiently nucleophilic to play a role in frustrated Lewis-pair like reactivity.

## Abstract 2

The synthesis of an arsanyl-phosphagallene [H_2_CN(Dipp)]_2_AsP=Ga(NacNac) (NacNac = HC[C(Me)N(Dipp)]_2_; Dipp = diisopropylphenyl) and its reactivity towards heterocumulenes and ketones is described.

## Graphical abstract

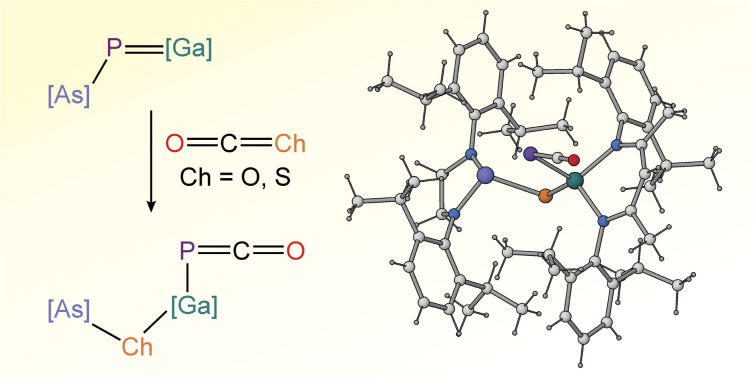

## Introduction

Compounds containing multiple bonds between the heavier main group elements (*n* > 2) are of fundamental interest due to their relationship with unsaturated organic molecules such as alkenes and alkynes. Recently, such species have attracted attention due to their ability to activate small molecule substrates.^1–3^ Pioneering research in the early 1980s by West and Yoshifuji, on disilenes and diphosphenes, respectively,^4^ paved the way for the successful isolation of a variety of stable homo-diatomic compounds.^5,6^ Since then, various hetero-diatomic compounds have also been reported.^6^ These include species with double bonds between the following element combinations: G13=G14,^7,8^ G13=G16,^9^ G14=G15,^10–15^ and G15=G16.^16–18^ Compounds with double bonds between elements of the group 13 and 15 are of special interest due to their valence isoelectronic relationship with C=C bonds. These species can be challenging to access due to the proximity of Lewis basic and Lewis acidic sites. Nonetheless, due to their increased G13=G15 bond polarity, the reactivity of these compounds is expected to differ significantly from their carbon-based analogues, making them interesting synthetic targets. Even though a handful of stable G13=G15 compounds have been synthesized recently,^19–27^ the reactivity of these compounds towards small molecules, especially towards heterocumulenes, remains largely unexplored. A recent review article published by Hering-Junghans and co-workers provides a detailed overview of compounds with G13=G15 double bonds, their syntheses and reactivity.^28^

**Fig. 1:**
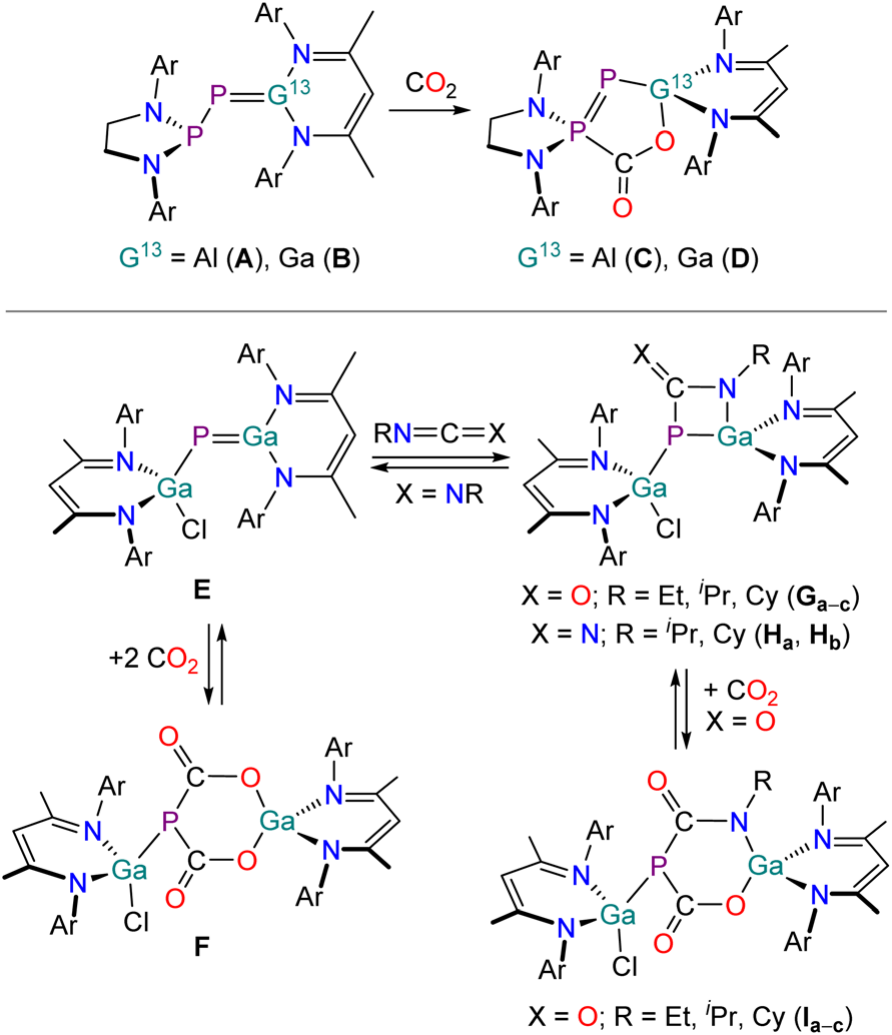
Previously reported phosphagallenes (B and E) and their reactivity towards heterocumulenes. Ar = 2,6-diisopropylphenyl (Dipp).

We recently reported that the phosphanyl-substituted phosphaalumene (A) and phosphagallene (B) can be accessed by reaction of a phosphaketene [H_2_CN(Dipp)]_2_PPCO (Dipp = 2,6-^*i*^Pr_2_C_6_H_3_) with group 13 carbenoids E(NacNac) (E = Al, Ga; NacNac = HC[C(Me)N(Dipp)]_2_) (Fig. 1, top).^29,30^ These compounds react with small molecules in frustrated Lewis pair (FLP)-type manner (*e.g.* H_2_, CO_2_, amines) or in hydroelementation reactions (*e.g.* silanes). Due to its highly polarized Al=P multiple bond compound A also undergoes intramolecular C–H activation reactions. To date, the only reported reactions of heterocumulenes with such compounds involve the reactions of A and B with CO_2_. This was found to selectively and irreversibly afford the five-membered ring systems C and D, respectively,^29,30^ as previously reported for other FLPs.^31–40^ Schulz and co-workers also recently reported the synthesis of a gallium-substituted phosphagallene (E) by reaction of the gallium phosphaketene (NacNac)Ga(Cl)PCO with Ga(NacNac) (Fig. 1, bottom).^41,42^ In contrast to A and B, the reactivity of E is mainly dominated by (partly reversible) reactions across the Ga=P multiple bond, involving 1,2-addition reactions (*e.g.* amines, alcohols, thioles, selenoles)^41^ and cycloaddition reactions (with heterocumulenes, ketones).^42,43^ Reaction of E with CO_2_ yields F, which readily loses CO_2_ upon heating to 95 °C (Fig. 1, bottom). Compound E reacts with isocyanates RNCO (R = Et, ^*i*^Pr, Cy) in (2 + 2) cycloaddition reactions with the C=N bonds yielding the four-membered heterocycles G_a–c_. Carbodiimides also undergo cycloaddition reactions yielding the products H_a_ and H_b_. When the heterocyclic products G_a–c_ were reacted with CO_2_ the formation of six-membered ring compounds I_a–c_ analogous to compound F was observed.

In relation to the aforementioned studies, the compounds K[Al(NON^Dipp^)(NMes)] and K[In(NON^Ar^)(NMes)] (NON^Ar^ = [O(SiMe_2_NAr)_2_]^2−^, Ar = Dipp; Mes = 2,4,6-Me_3_C_6_H_2_) which exhibit G13=N (G13 = Al, In) double bonds also exhibit interesting reactivity towards heterocumulenes. The aluminum complex reacts with CO_2_ resulting in the formation of a dimeric carbamate dianion in a (2 + 2)-addition reaction.^44^ The reaction of the corresponding indium derivative with azides RN_3_ (R = Mes, SiMe_3_) affords tetrazole analogues.^45^

The reactivity of phosphagallenes appears to be sensitive to substitution effects, as proven by the differing reactivity of B and E (*vide supra*). Thus, we were interested in how exchanging the adjacent basic phosphanyl group in B with a less basic arsanyl moiety affects the reactivity of the resulting species towards small molecules. These studies are described herein.

## Results and discussion

### Synthesis of [H_2_CN(Dipp)]_2_AsP=Ga(NacNac) (1)

**Scheme 1:**
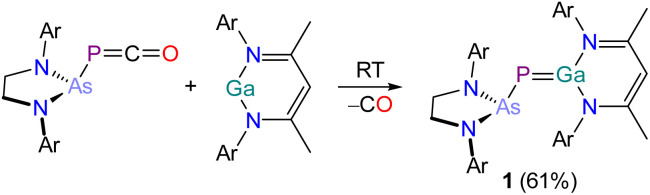
Synthesis of 1. Ar = 2,6-diisopropylphenyl (Dipp).

**Fig. 2:**
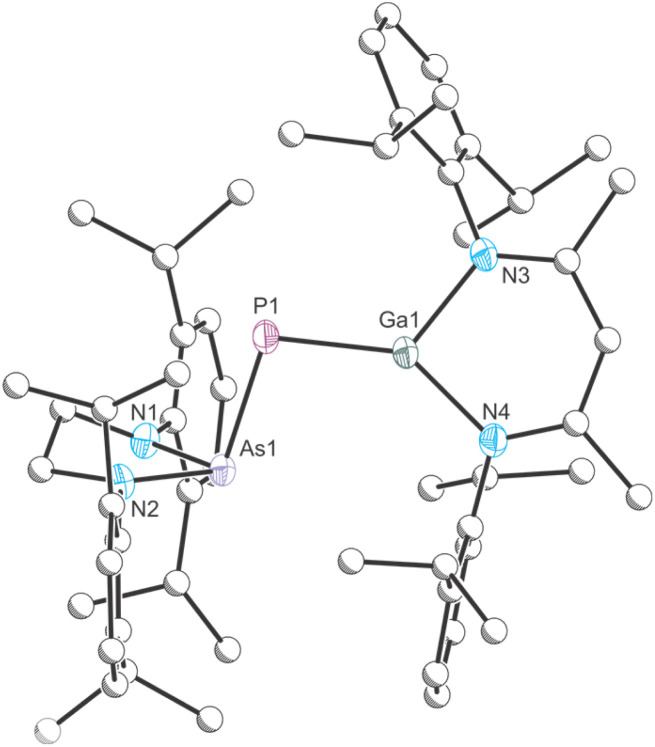
Single crystal X-ray structure of 1. Thermal ellipsoids set at 50% probability level; hydrogen atoms and solvent of crystallization omitted for clarity. Carbon atoms are depicted as spheres of arbitrary radius. Selected interatomic distances [Å] and angles [°]: As1–P1 2.3166(4), P1–Ga1 2.1734(4), As1–P1–Ga1 99.703(15).

Dissolution of an equimolar ratio of [H_2_CN(Dipp)]_2_AsPCO^46^ and Ga(NacNac) in toluene at RT resulted in rapid effervescence and an immediate color change of the reaction mixture from yellow to deep red (Scheme 1). The ^31^P{^1^H} NMR spectrum of the solution shows the quantitative formation of a product with a chemical shift of −72.2 ppm, which is significantly deshielded relative to the starting material (*cf.* [H_2_CN(Dipp)]_2_AsPCO: −256.8 ppm). A comparable shift was observed for the formation of B (*cf.* [H_2_CN(Dipp)]_2_P*P*CO: −245.6 ppm; [P]*P*[Ga] −61.3 ppm).^30^ Product 1 crystallizes in form of deep red single crystals from saturated *n*-hexane or *n*-pentane solution at RT in the monoclinic space group *P*2_1_/*c* (Fig. 2). The unit cell parameters of 1 are very similar to the parameters observed for B (see Table S2‡). The crystal structure of 1 reveals a Ga1–P1 bond length of 2.1734(4) Å which suggests the presence of a double bond (∑*r*_cov_ (Ga–P) = 2.35 Å; ∑*r*_cov_ (Ga=P) = 2.19 Å).^47,48^ The P1–As1 bond length of 2.3166(4) Å is in the range of a single bond (∑*r*_cov_ (P–As) = 2.32 Å; ∑*r*_cov_ (P=As) = 2.16 Å).^47,48^

No subsequent reactivity was observed on heating 1 in C_6_D_6_ solution to 80 °C over a period of five days. Dissolution of 1 in THF reveals no evidence of adduct formation, as evidenced by the fact that the ^31^P{^1^H} NMR chemical shift does not change.

## Additions involving the Ga=P bond

### Reaction of 1 towards azides

**Scheme 2:**
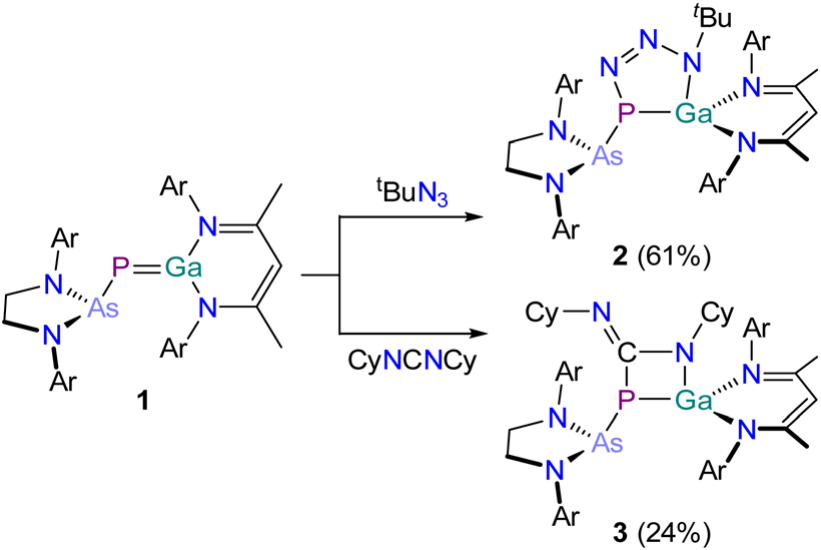
Synthesis of 2 (top) and 3 (bottom). Ar = 2,6-diisopropylphenyl (Dipp).

**Fig. 3:**
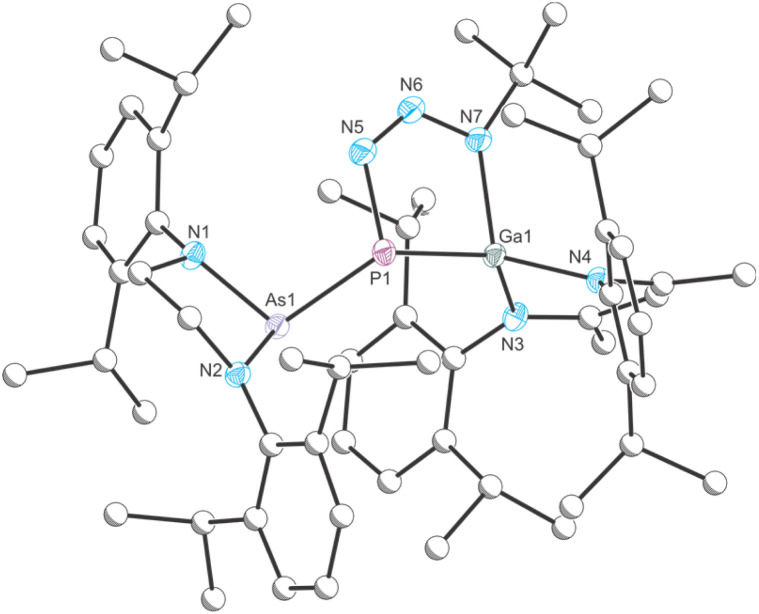
Single crystal X-ray structure of 2. Thermal ellipsoids set at 50% probability level; hydrogen atoms and solvent of crystallization omitted for clarity. Carbon atoms are depicted as spheres of arbitrary radius. Selected interatomic distances [Å] and angles [°]: As1–P1 2.4232(4), P1–Ga1 2.2782(3), P1–N5 1.7794(12), N5–N6 1.2738(17), N6–N7 1.3456(16), N7–Ga1 1.9843(11), As1–P1–Ga1 109.576(14).

Upon addition of *tert*-butyl azide to a red solution of 1 in C_6_H_6_ at room temperature the reaction mixture instantly turns orange (Scheme 2, top). The ^31^P{^1^H} NMR spectrum suggests quantitative conversion to a single species, 2, which appears as a singlet at −31.6 ppm. Compound 2 crystallizes in the triclinic space group *P*1̄ from a concentrated *n*-hexane/benzene solution in form of light orange crystals suitable for X-ray crystallography (Fig. 3). The crystal structure reveals the formation of a (2 + 3) addition product exhibiting a five-membered GaPN_3_ ring system. The reaction proceeds regio-selectively with the functionalized nitrogen atom of the azide selectively bonded to the gallium atom. The Ga–N7, Ga1–P1 and P1–N5 bond legnths are as expected for single bonds. The N6–N7 bond is slighty longer than the N5–N6 bond, but both lie in between what is expected for a N–N single bond and a N=N double bond. The GaPN_3_ ring system in 2 is not planar but slightly twisted. When evaporating a light orange, crystalline sample of 2 for one hour at room temperature, the orange color slowly intensifies/darkens. The same color change can be observed when heating a sample of 2 in C_6_D_6_ to 80 °C for one hour. The ^31^P{^1^H} NMR spectrum of this heated solution shows that 1 is reformed, indicating that the addition of the azide is reversible. This makes the isolation of compositionally pure samples of 2 challenging as they are often contaminated with the phosphagallene starting material.

### Reactivity of 1 towards carbodiimides

**Fig. 4:**
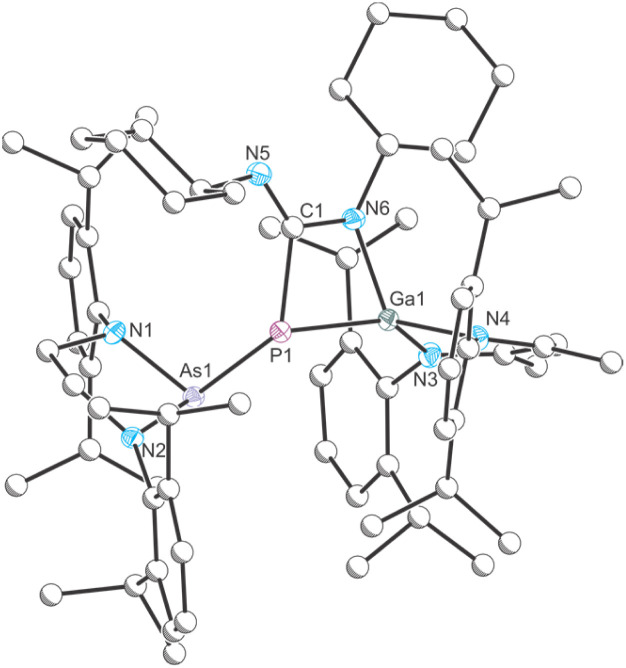
Single crystal X-ray structure of 3. Thermal ellipsoids set at 50% probability level; hydrogen atoms and solvent of crystallization omitted for clarity. Carbon atoms are depicted as spheres of arbitrary radius. Selected interatomic distances [Å] and angles [°]: As1–P1 2.3887(7), P1–Ga1 2.3188(7), P1–C1 1.916(3), C1–N6 1.386(3), N6–Ga1 1.918(2), C1–N5 1.273(3), As1–P1–Ga1 106.21(3).

Schulz and co-workers have previously reported that E undergoes (2 + 2) cycloaddition reactions with carbodiimides yielding four-membered heterocycles H_a_ and H_b_ (*vide supra*).^42^ The same reactivity was observed on reaction of 1 with *N*,*N*′-dicyclohexylcarbodiimide C(NCy)_2_. Upon the addition of the carbodiimide to a red solution of 1 in C_6_H_6_ at room temperature the mixture quickly turns yellow (Scheme 2, bottom). The product, 3, exhibits a singlet in its ^31^P{^1^H} NMR spectrum at −27.6 ppm, and the resonance of the N*C*N atom appears as a doublet in the ^13^C{^1^H} NMR spectrum at 164.0 ppm (^1^*J*_P–C_ = 68.1 Hz). 3 crystallizes from benzene at room temperature and X-ray crystallography confirms the formation of a slightly puckered four-membered PCNGa heterocycle (Fig. 4). The P1–Ga1 (2.3188(7) Å; ∑*r*_cov_ (Ga–P) = 2.35 Å), Ga1–N6 (1.918(2) Å; ∑*r*_cov_ (Ga–N) = 1.95 Å) and the P1–C1 bond lengths (1.916(3) Å; ∑*r*_cov_ (P–C) = 1.86 Å) are in the range of single bonds. The C1–N5 bond length (1.273(3) Å) is as expected for an average double bond, while the C1–N6 bond (1.386(3) Å) is notably shorter than expected for a single bond (∑*r*_cov_ (C–N) = 1.46 Å; ∑*r*_cov_ (C=N) = 1.27 Å). This shortened bond length was also observed in an analogous product from the reaction of E with carbodiimides.^42^

### Reactivity of 1 towards isocyanates

**Scheme 3:**
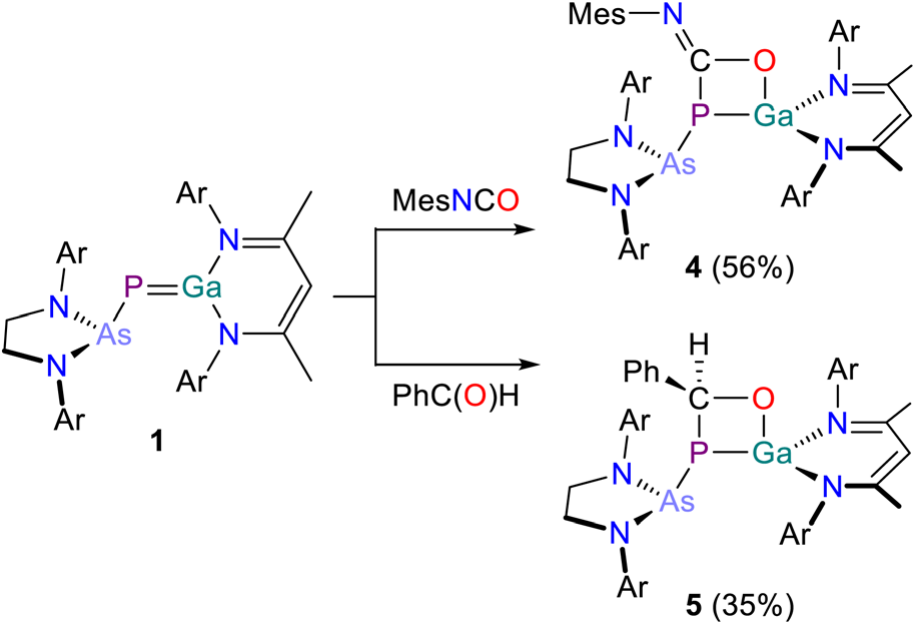
Synthesis of 4 (top) and 5 (bottom). Ar = 2,6-diisopropylphenyl (Dipp).

**Fig. 5:**
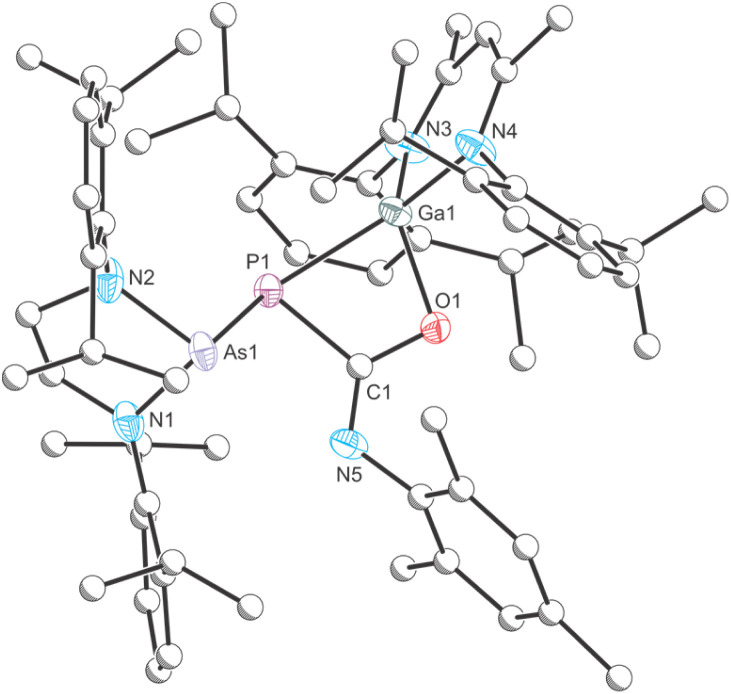
Single crystal X-ray structure of 4. Thermal ellipsoids set at 50% probability level; hydrogen atoms and solvent of crystallization omitted for clarity. Carbon atoms are depicted as spheres of arbitrary radius. Selected interatomic distances [Å] and angles [°]: As1–P1 2.3811(6), P1–Ga1 2.3209(6), P1–C1 1.853(2), C1–O1 1.343(3), O1–Ga1 1.9089(15), As1–P1–Ga1 123.94(2).

Reaction of 1 with a slight excess of mesityl isocyanate MesNCO results in a slow color change of the reaction mixture overnight from red to bright yellow (Scheme 3, top). The ^31^P{^1^H} NMR spectrum of the solution shows selective conversion to a species exhibiting a singlet resonance at −34.5 ppm, indicating the formation of a new compound 4. This is further supported by the ^13^C{^1^H} NMR spectrum which exhibits a doublet resonance at 139.9 ppm (^1^*J*_C–P_ = 46.3 Hz) arising from the heteroallenic isocyanate carbon atom. Crystals suitable for X-ray crystallography were grown from benzene solution at room temperature (Fig. 5). The crystal structure confirms the formation of a (2 + 2) addition product as observed for reactions with carbodiimides. In contrast to the reactivity reported by Schulz and co-workers for E, the addition of MesNCO to 1 selectively proceeds at the C=O bond of the isocyanate, yielding a four-membered ring system consisting of Ga1, P1, C1 and O1. The Ga1–P1 bond (2.3209(6) Å), the Ga1–O1 bond (1.9089(15) Å; ∑*r*_cov_ (Ga–O) = 1.87 Å), the P1–C1 bond (1.853(2) Å) and the C1–O1 bond (1.343(3) Å; ∑*r*_cov_ (C–O) = 1.38 Å) are all in the range of corresponding single bonds. The four-membered GaPCO ring system is also slightly puckered, the dihedral angle between the P1/C1/O1 plane and P1/Ga1/O1 plane is 162.59(10)°.

### Reactivity of 1 towards benzaldehyde

Given the contrasting reactivity of 1 and E towards isocyanates, we were intrigued to investigate the reactivity of 1 towards other carbonyl-containing compounds. For this purpose, we chose to investigate its reactivity towards benzaldehyde. In addition to its ability to undergo (2 + 2) addition reactions, benzaldehyde also has a relatively protic C(sp^2^)–H bond that could react with the Ga=P bond.

**Fig. 6:**
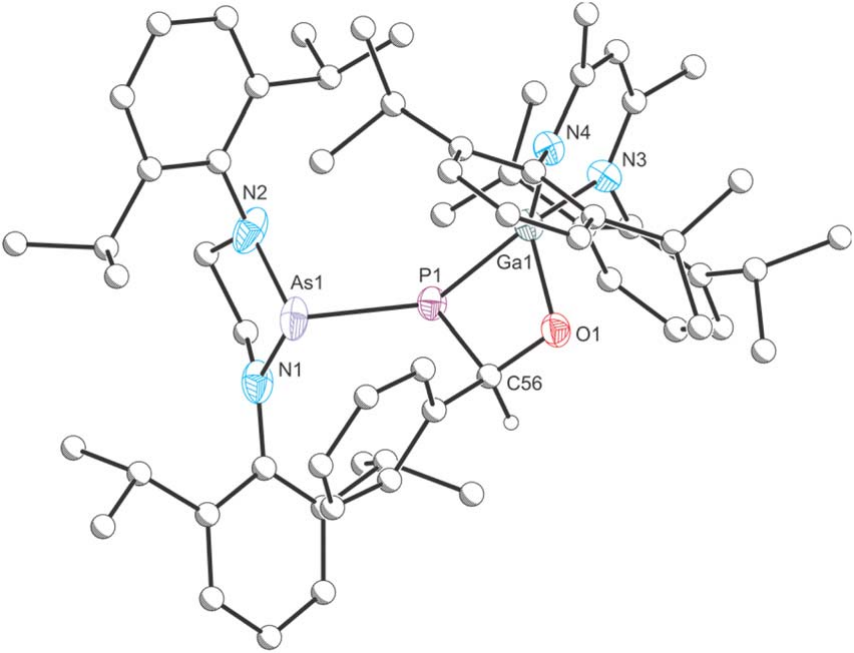
Single crystal X-ray structure of 5. Thermal ellipsoids set at 50% probability level; hydrogen atoms and solvent of crystallization omitted for clarity. Carbon atoms are depicted as spheres of arbitrary radius. Selected interatomic distances [Å] and angles [°]: As1–P1 2.3821(5), P1–Ga1 2.3457(5), P1–C56 1.929(2), C56–O1 1.423(2), O1–Ga1 1.8633(15), As1–P1–Ga1 124.17(2).

Addition of benzaldehyde to a red solution of 1 in C_6_H_6_ at room temperature the reaction solution instantly turns yellow (Scheme 3, bottom). The ^31^P{^1^H} NMR spectrum of the reaction mixture indicates selective formation of a new product, 5, which shows a singlet resonance at 67.6 ppm. The ^13^C{^1^H} NMR spectrum reveals a doublet at 79.3 ppm (^1^*J*_C–P_ = 27.3 Hz) which can be assigned to the Ph*C*HO atom. Crystals suitable for X-ray crystallography were grown from a saturated benzene/*n*-hexane solution. 5 is the product of a (2 + 2) addition reaction along the C=O bond, yielding a four-membered GaPCO heterocycle (Fig. 6). This heterocycle and its molecular parameters are similar to those observed for compound 4. The Ga1–O1 bond length (1.8633(15) Å) is in the range expected for a single bond, as are the Ga1–P1 (2.3457(5) Å), the P1–C56 (1.929(2) Å) and the C56–O1 bond lengths (1.423(2) Å). The ring system is again not planar but slighty puckered, the dihedral angle between the P1–C56–O1 plane and P1–Ga1–O1 plane is 166.35(11)°.

## Ga=P bond insertion reactions and phosphorus atom extrusion

### Reactivity of 1 towards CO_2_

The aforementioned reactions involve the formation of addition products of heterocumulenes or ketones with the Ga=P bond of 1. Given that closely related compounds such as A and B have been shown to exhibit FLP-like reactivity towards other heterocumulenes, *e.g.* CO_2_, we were interested to explore the role played by the pendant phosphanyl/arsanyl moiety in these reactions. The arsanyl moiety in 1 is notably less Lewis basic than the phosphanyl moiety present in B, and thus the reactivity of this compound should differ significantly.^49^

**Scheme 4:**
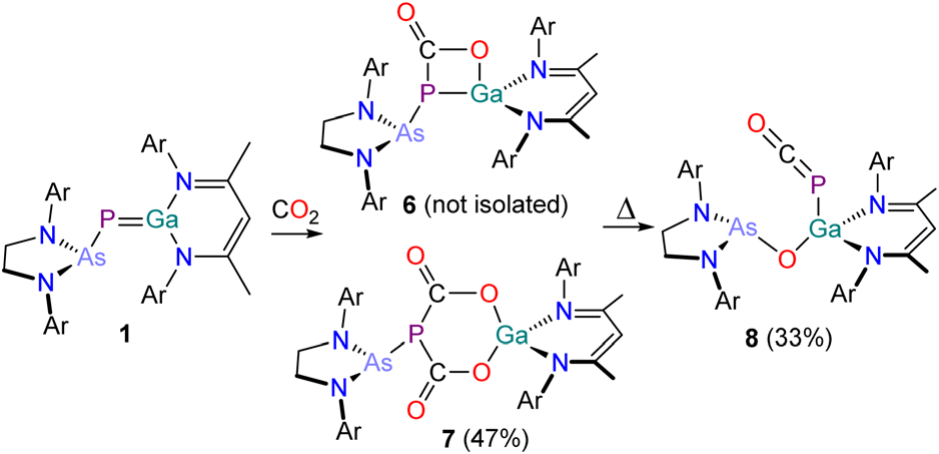
Synthesis of 6, 7 and 8. Ar = 2,6-diisopropylphenyl (Dipp).

Reaction of a C_6_D_6_ solution of B with 2 bar of CO_2_ at room temperature was reported to selectively yield the colourless five-membered ring system D which exhibits two doublet resonances in its ^31^P{^1^H} NMR spectrum at 80.7 ppm and −291.0 ppm (^1^*J*_P–P_ = 588 Hz).^30^ Contrastingly, the analogous reaction of 1 with CO_2_ yields an orange reaction mixture containing multiple products (Scheme 4).

**Fig. 7:**
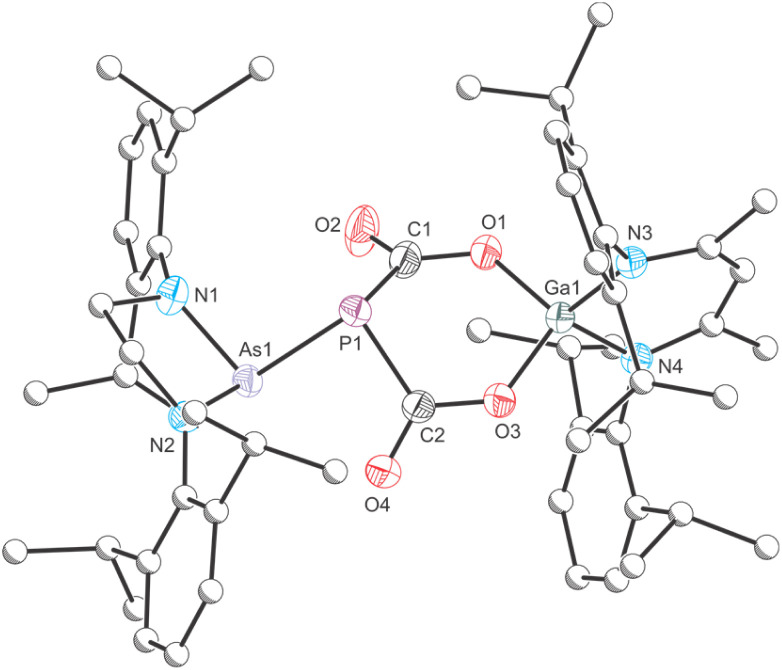
Single crystal X-ray structure of 7. Thermal ellipsoids set at 50% probability level; hydrogen atoms and solvent of crystallization omitted for clarity. Carbon atoms (except for C1 and C2) are depicted as spheres of arbitrary radius. Selected interatomic distances [Å] and angles [°]: As1–P1 2.3985(10), P1–C1 1.874(4), P1–C2 1.873(4), Ga–O1 1.852(3), Ga1–O3 1.831(3), C1–O1 1.319(5), C1–O2 1.204(5), C2–O3 1.321(4), C2–O4 1.210(5), As1–P1–C1 98.29(15), As1–P1–C2 92.63(11), C1–P1–C2 100.99(18).

Directly after addition, the ^31^P{^1^H} NMR spectrum of the reaction mixture exhibits three singlets at −72.2 (unreacted 1), −29.5 (6) and −9.0 ppm (7). After one day at room temperature, the reaction solution decolorizes and exclusively exhibits one singlet at −9.0 ppm (see Fig. S11‡). The corresponding product, 7, can be crystallized from *n*-pentane at −30 °C in form of thin, colourless needles. X-ray crystallography reveals that 7 is the formal insertion product of two equivalents of CO_2_ into the Ga=P bond of 1 (Fig. 7). This motif has been reported previously by Schulz and co-workers on reaction of E with CO_2_.^43^ The six-membered GaO_2_C_2_P-heterocycle is almost planar, with the phosphorus atom protruding from the ring plane (*Σ*_angles_ = 291.91°). The Ga–O bonds (Ga–O1 1.852(3) Å; Ga–O3 1.831(3) Å) are nearly identical and in the range of typical single bonds, as are the C–O bonds in the heterocycle (C1–O1 1.319(5) Å; C2–O3 1.321(4) Å) and the P–C bonds (P1–C1 1.874(4) Å; P1–C2 1.8743(4) Å; ∑*r*_cov_ (P–C) = 1.86 Å).

Given that E has been shown to reversibly bind CO_2_,^43^ we were curious to probe the thermal stability of 7. Heating a colorless solution of 7 in C_6_D_6_ to 80 °C for one-hour results in an intense orange solution. This solution exhibits traces of 6 and the starting material 1 in its ^31^P{^1^H} NMR spectrum, indicating that the CO_2_ binding is thermally reversible. Upon prolonged heating, another small singlet with a chemical shift of −371.1 ppm corresponding to a new compound 8 arises in the ^31^P{^1^H} NMR spectrum. Heating a solution of 7 for three days at 80 °C cleanly affords 8. The ^13^C{^1^H} NMR spectrum of 8 shows a doublet at 181.2 ppm with a ^1^*J*_C–P_ coupling constant of 92.9 Hz. The IR spectrum of 8 reveals an absorption band at 1922 cm^−1^. These data are in line with those previously reported for gallium phosphaketenes (^31^P{^1^H} NMR shifts ranging from −354.9 ppm to −394.6 ppm; ^13^C{^1^H} NMR shifts in the range of 180.8 ppm to 186.4 ppm and ^1^*J*_C–P_ coupling constants ranging from 85 Hz to 101 Hz; PCO IR stretching bands between 1910 cm^−1^ and 1936 cm^−1^).^43,50,51^ To the best of our knowledge, there are only a handful of examples of deoxygenation of CO_2_ by compounds of the main group elements,^52–55^ while examples involving transition metals are more numerous.^56–60^

**Fig. 8:**
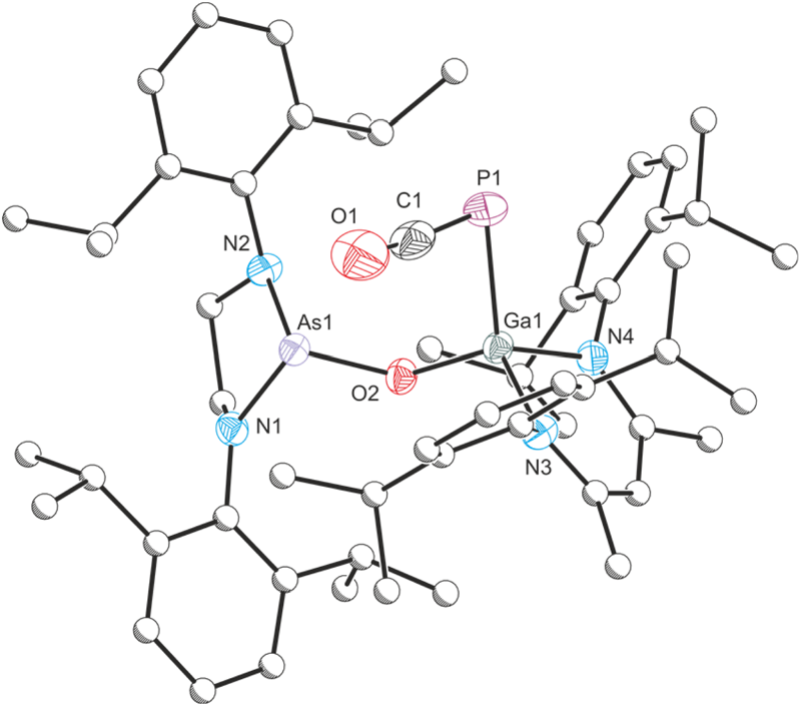
Single crystal X-ray structure of 8. Thermal ellipsoids set at 50% probability level; hydrogen atoms and solvent of crystallization omitted for clarity. Carbon atoms (except for C1) are depicted as spheres of arbitrary radius. Selected interatomic distances [Å] and angles [°]: As1–O2 1.7826(10), O2–Ga1 1.8220(10), Ga1–P1 2.3512(4), P1–C1 1.644(2), C1–O1 1.160(3), As1–O2–Ga1 125.12(6), Ga1–P1–C1 92.98(6), P1–C1–O1 174.38(17).

Compound 8 can be crystallized from a saturated *n*-hexane/benzene mixture at room temperature and X-ray elucidation confirms the formation of a gallium-phosphaketene with a Ga1–P1 bond length of 2.3512(4) Å (Fig. 8). Additionally, the Ga1 atom in 8 is bound to the arsanyl moiety *via* a bridging oxygen atom which originates from the carbon dioxide (Ga1–O1 1.8220(10) Å). The Ga1–P1–C1 angle of 92.98(6)° is typical for gallium phosphaketenes, as well as the almost linear P1–C1–O2 moiety (174.38(17)°).^43,50,51^

Having isolated and characterized species 7 and 8 from the reaction of 1 with CO_2_ [^31^P NMR: −9.0 (7) and −371.1 ppm (8)] we were intrigued by the nature of the unidentified intermediate observed in crude reaction mixtures which exhibits a singlet resonance at −29.5 ppm. Given that the chemical shift of this species is close to that of 3 and 4, we suspect that this compound arises from the (2 + 2) addition of CO_2_ and 1. This hypothesis is further supported by the observation that upon reaction of 1 with ^13^CO_2_, this resonance appears in the ^31^P{^1^H} NMR spectrum as a doublet with a ^1^*J*_C–P_ coupling of 37.5 Hz. Furthermore, the ^13^C{^1^H} NMR spectrum of the reaction mixture exhibits a doublet resonance for the P^13^*C*O_2_ moiety at 179.7 ppm (^1^*J*_C–P_ = 37.5 Hz; see ESI,‡ chapter 3).

In an attempt to characterize this compound, we halted the reaction 30 min after the addition of CO_2_ by removing the solvent and all volatiles under a dynamic vacuum. Redissolving the resulting orange residue in C_6_D_6_ shows the presence of 1 and of 7 by ^31^P{^1^H} NMR spectroscopy, with no evidence of 6. This suggests that the formation of 6 is reversible. Unfortunately, we were unable to isolate compound 6 from mixtures of CO_2_ and 1 despite repeated attempts. Varying the pressure of CO_2_ was attempted, giving rise to varying ratios of 1, 6, 7 and 8 at different stages of the reaction, however the reactions always contained product mixtures.

### Reactivity of 1 with CS_2_

**Scheme 5:**
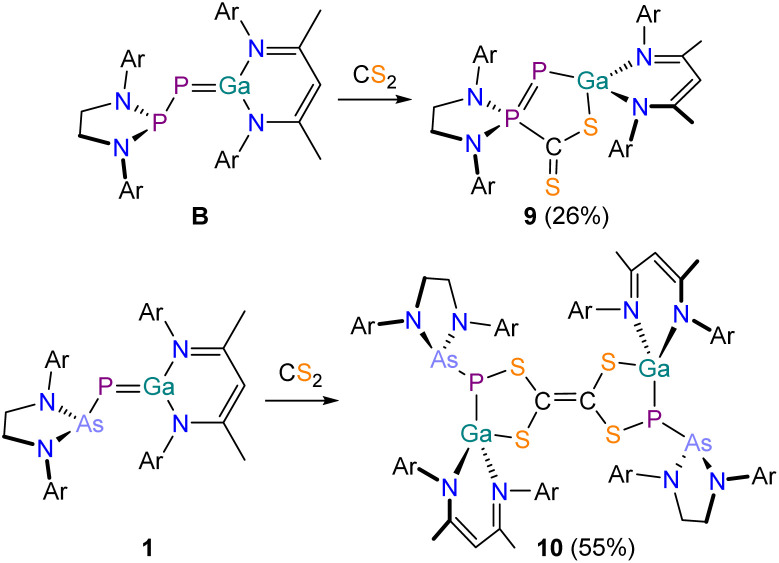
Contrasting reactivity of B (top) and 1 (bottom) with CS_2_. Ar = 2,6-diisopropylphenyl (Dipp).

**Fig. 9:**
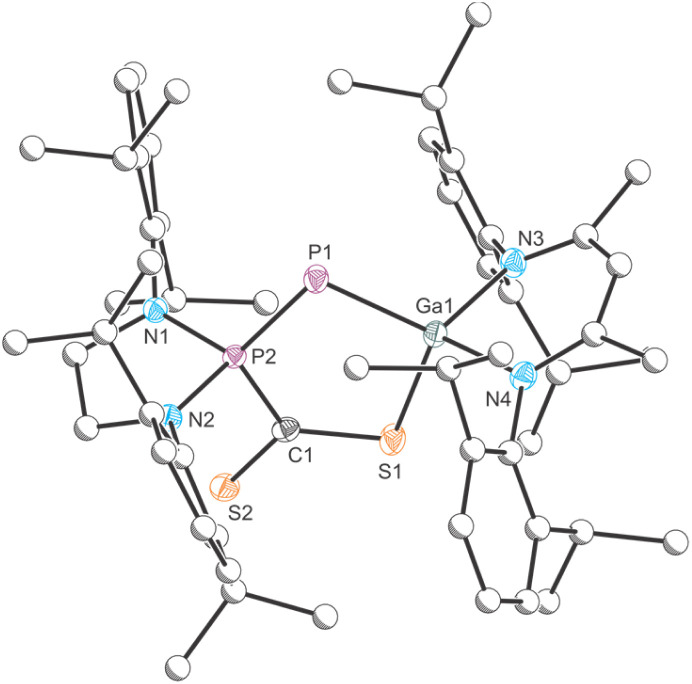
Single crystal X-ray structure of 9. Thermal ellipsoids set at 50% probability level; hydrogen atoms and solvent of crystallization omitted for clarity. Carbon atoms (with the exception of C1) are depicted as spheres of arbitrary radius. Selected interatomic distances [Å] and angles [°]: P1–P2 2.0630(6), P1–Ga1 2.2874(5), P2–C1 1.877(2), C1–S1 1.715(2), C1–S2 1.6477(19), S1–Ga1 2.2935(5), P2–P1–Ga1 95.89(2).

Due to the significant differences in the reactivity of B and 1 towards CO_2_ we investigated the thus far unexplored reactivity of both species towards carbon disulfide CS_2_. Reaction of B with CS_2_ in C_6_H_6_ resulted in a color change of the solution from red to a dark red/purple (Scheme 5, top). The ^31^P{^1^H} NMR spectrum of the reaction exhibits two doublets at 102.7 and −287.3 ppm (^1^*J*_P–P_ = 614.0 Hz). The product, 9, crystallizes from *n*-hexane at −30 °C in form of purple needles. X-ray crystallography confirms the formation of a formal FLP-type activation product, analogous to the CO_2_ activation product D (Fig. 9). 9 exhibits a significantly contracted P1–P2 bond (2.0630(6) Å; ∑*r*_cov_ (P=P) = 2.04 Å) which is in the range of a corresponding double bond.^48^ The Ga1–P1 bond (2.2874(5) Å) is enlogated in comparison to B and in the range of a single bond.^47,48^ Adduct formation is not reversible under mild conditions (*e.g.* evaporation at room temperature or heating in C_6_D_6_ solution to 80 °C overnight).

**Fig. 10:**
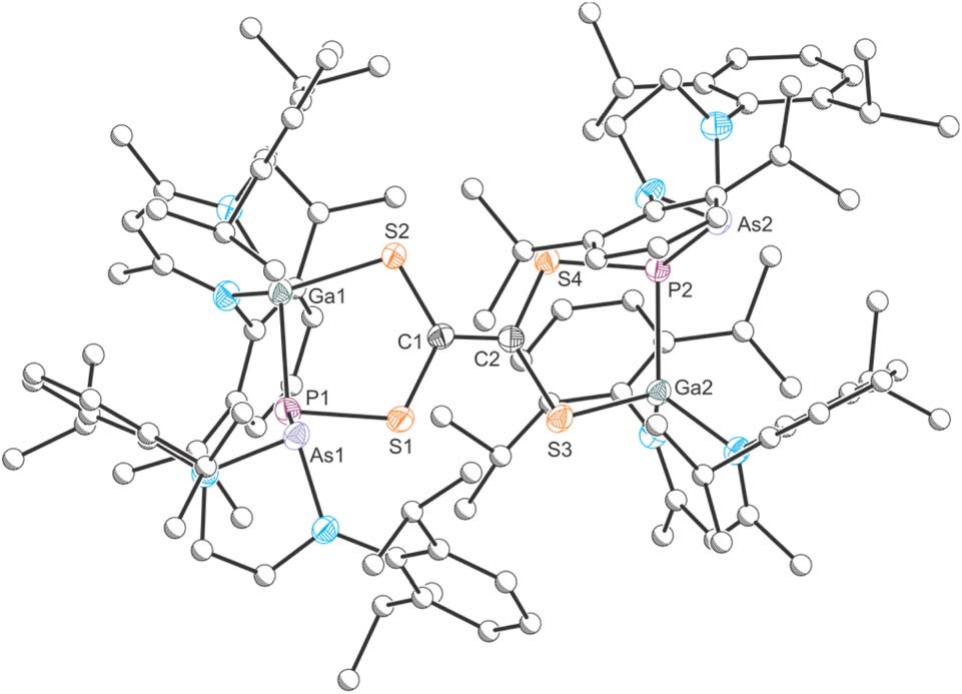
Single crystal X-ray structure of 10. Thermal ellipsoids set at 50% probability level; hydrogen atoms and solvent of crystallization omitted for clarity. Carbon atoms (with the exception of C1 and C2) are depicted as spheres of arbitrary radius. Selected interatomic distances [Å] and angles [°]: As1–P1 2.4056(7), P1–Ga1 2.3544(7), P1–S1 2.1175(9), S1–C1 1.787(3), C1–S2 1.756(2), S2–Ga1 2.2349(7), C1–C2 1.350(3), As2–P2 2.3994(6), P2–Ga2 2.3103(7), P2–S4 2.0994(8), S4–C2 1.804(2), C2–S3 1.760(2), S3–Ga2 2.2574(7).

By contrast, the ^31^P{^1^H} NMR spectrum of a C_6_D_6_ solution of 1 after treatment with excess CS_2_ shows two singlets at −25.9 and −52.5 ppm in a 1 : 1 ratio. The ^1^H NMR spectrum displays two singlets at 4.80 and 4.67 ppm stemming from two inequivalent NacNac γ-H protons, also in a 1 : 1 ratio. Single crystals of the product 10 were grown from *n*-pentane at room temperature (Fig. 10). X-ray elucidation reveals the formation of a dimeric compound, in which two identical moieties are connected *via* a C1=C2 bond (1.350(3) Å; ∑*r*_cov_ (C=C) = 1.34 Å).^47^ This suggests that a carbene-like intermediates arising from a (2 + 3) addition reaction of 1 and CS_2_ dimerizes in the reaction solution to form 10. Mayer and coworkers reported on a similar product formed in the reaction of a disilene with CS_2_.^61^ A similar C_2_S_4_^2−^ moiety was recently accessed by reaction of an anionic gallium(i) compound with CS_2_.^62^ The two hereocycles in 10 are crystallographically inequivalent, but their bond metric parameters are very similar. The P–Ga bonds (P1–Ga1 2.3544(7) Å; P2–Ga2 2.3103(7) Å) are in the range expected for single bonds, as are the Ga–S bonds (Ga1–S2 2.2349(7) Å; Ga2–S3 2.2574(7) Å; ∑*r*_cov_ (Ga–S) = 2.27 Å) and the C–S bonds (C1–S1 1.787(3) Å; C1–S2 1.756(2) Å; C2–S3 1.760(2) Å; C2–S4 1.804(2) Å; ∑*r*_cov_ (C–S) = 1.78 Å). Compound 10 is stable at room temperature and does not undergo any rearrangement reactions even when heating a solution to 80 °C in C_6_D_6_. Given the structural similarities of 10 to tetrathiafulvalene, this product may exhibit interesting redox behavior and electrochemical properties. The formation of 10 rather than of an FLP-type activation product analogous to 9 can be rationalized due to the less nucleophilic arsanyl moiety of 1. However, to rationalize the differing reactivity of 1 towards CO_2_ and CS_2_, we performed more detailed calculations (*vide infra*).

### Reactivity of 1 towards COS

**Scheme 6:**
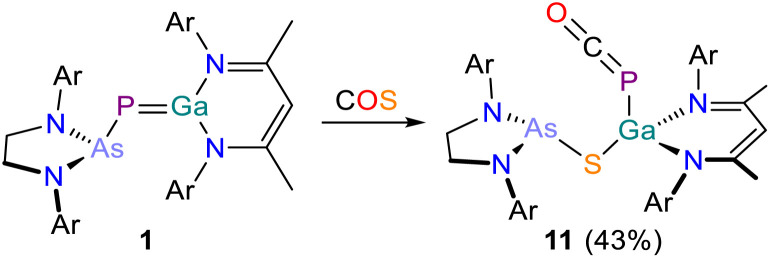
Synthesis of 11. Ar = 2,6-diisopropylphenyl (Dipp).

**Fig. 11:**
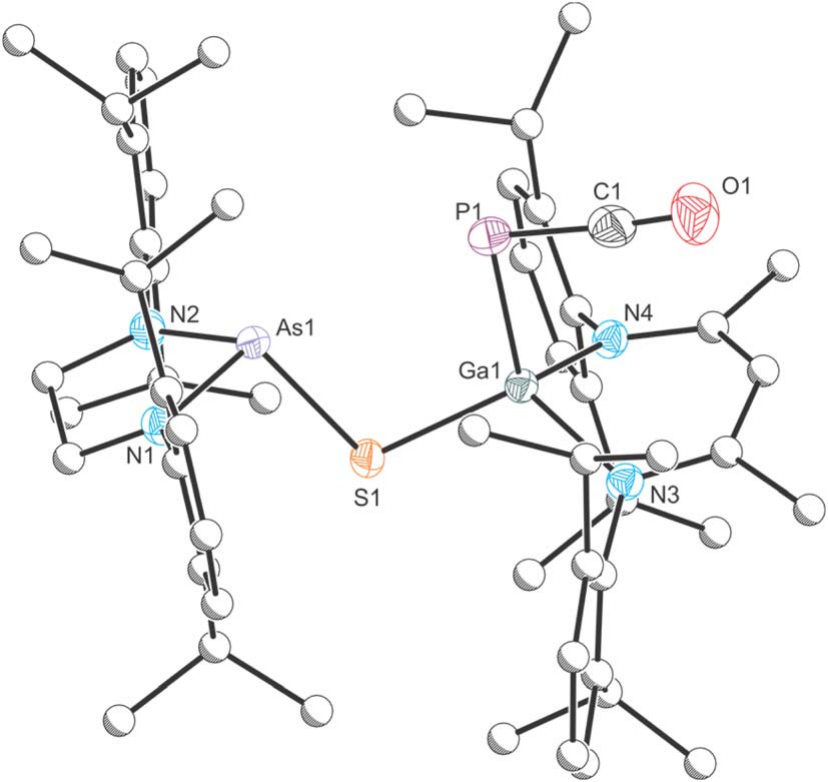
Single crystal X-ray structure of 11. Thermal ellipsoids set at 50% probability level; hydrogen atoms and solvent of crystallization omitted for clarity. Carbon atoms (with the exception of C1) are depicted as spheres of arbitrary radius. Selected interatomic distances [Å] and angles [°]: As1–S1 2.3216(7), S1–Ga1 2.2261(7), Ga1–P1 2.3877(8), P1–C1 1.642(4), C1–O1 1.165(5), As1–S1–Ga1 99.77(3), Ga1–P1–C1 95.25(11), P1–C1–O1 173.6(3).

To better understand the differing reactivity of 1 towards CO_2_ and CS_2_, we reacted 1 with carbonyl sulfide COS (Scheme 6). On addition of COS, the color of the solution changed from deep red to light yellow within a minute. The ^31^P{^1^H} NMR spectrum of the reaction mixture revealed the formation of a single species, 11, exhibiting a singlet resonance at −365.4 ppm. In contrast to the reactions between 1 with CO_2_ (*vide supra*) which required elevated temperatures, 11 is formed immediately upon addition of COS at room temperature, and no intermediate species could be observed spectroscopically. 11 crystallizes from saturated *n*-pentane/toluene solution at room temperature in form of colorless blocks in the space group *Pna*2_1_ with two inequivalent molecules per unit cell (for clarity bond metric data for only one crystallographically independent molecule is discussed). Single-crystal X-ray analysis confirms the formation of a phosphaketene-containing compound analogous to 7 (Fig. 11). The Ga–P bond lengths are in the range of a corresponding double bond (2.3877(8) Å). In contrast to 7, the Ga1 atom in 11 is bound to the arsanyl moiety *via* a bridging sulfur atom (Ga1–S1 2.2261(7) Å). Compound 11 exhibits an almost linear phosphaketenyl moiety (P–C–O 173.6(3)°). In the IR spectrum the absorption band of the PCO stretching vibration can be found at 1907 cm^−1^.

## Computations

### Electronic structure

To gain further structural insights, density functional theory (DFT) calculations^63^ and natural bond orbital (NBO)^64–66^ analyses were performed using Gaussian09 (ref. 67) at the PBE level of theory^68–70^ using the def2-TZVP basis set^71^ with dispersion correction (D3(BJ);^72,73^ notation: PBE-D3/def2-TZVP). The bond parameters of 1_DFT_ were found to be similar to the experimentally observed ones (Ga–P: 2.1688 Å and P–As: 2.3175 Å). The natural population analysis (NPA) of 1_DFT_ reveals that the Ga1–P1 π-bond in 1_DFT_ has an occupancy of 1.87 e, (indicating a slight delocalization to the neighboring Ga–N(NacNac) bonds). This bond is almost exclusively formed from p atomic orbitals (Ga1: 99%, P1: 99% p-character) and is significantly polarized towards the P1 atom (Ga1: 17%, P1: 83%). The Ga1 atom (+1.32) and the As1 atom (+1.03) are positively charged, whilst the P1 atom is negatively charged (−0.86). Natural bond analysis (NBO) reveals a Wiberg bond index of 1.45 for the Ga–P bond in 1_DFT_ and of 1.03 for the respective P–As bond.

A comparison of the parameters calculated for 1_DFT_ and the analogues A_DFT_ and B_DFT_ is well-suited to explain the experimentally observed differences in reactivity of these species. In both A_DFT_ and B_DFT_ the HOMO is mainly located on the lone pairs of the P1 and P2 atoms, whereas the HOMO−1 reflects the respective π-bonding interations along the G13–P1 bond (see Fig. S15‡). In accordance with these findings, A and B show FLP-type reaction behavior upon the activation of small molecules such as *e.g.* H_2_, CO_2_, CS_2_ and amines.^30,74^ Exchanging the [Ga] moiety in B with an [Al] moiety results in an increased reactivity, *e.g.* towards CH bonds, and also reduces the stability of A in comparison to B. This can be rationalized with the higher polarization of the G13=P multiple bond in A.

In contrast, the HOMO in 1_DFT_ reflects the π-bonding interations along the Ga1–P1 bond and the HOMO−1 is located at the lone pairs of the P1 and the As1 atoms. Thus, the reactivity of 1 is mainly dominated by its Ga=P multiple bond (*vide infra*). Exchanging the pendant phosphanyl [P] moiety with an arsanyl [As] moiety not only affects the energies of relevant orbitals, but also orbital shape and hybridization. According to NBO analysis, the lone pair at the As1 atom in 1_DFT_ exhibits a significantly higher s-character (69%) than the analogous lone pair at the P2 atom in B_DFT_ (58%). Additionally, the LP at the As1 atom in 1_DFT_ is more diffuse. Both factors result in a less localized electron charge at the As1 atom, and thus in a decreased basicity of the As1 atom in 1_DFT_, in comparison to the basicity of the P2 atom in B_DFT_. These findings are suitable to rationalize why 1_DFT_ does not exhibit FLP-type reactivity, as proven experimentally (*vide ante*).

### Reaction mechanisms

Compound B was experimentally found to react with CO_2_ and CS_2_ in a FLP-type fashion, yielding analogous products for both CO_2_ and CS_2_. However, we were surprised by the differing reactivity of 1 towards CO_2_, CS_2_, and COS. Using ORCA 5.0.4 ^75^ and the NEB method (nudged elastic band),^76–79^ we calculated the Gibbs free energies of potential reaction products and reaction mechanisms of formal (2 + 2) and (3 + 2) addition reactions of 1 and CO_2_, CS_2_ or COS, respectively. Regarding the molecular frontier orbitals of CO_2_, CS_2_ and 1_DFT_ (see Fig. S16‡) we would expect a formal (2 + 2) addition reaction to proceed in a stepwise manner, as opposed to a concerted (3 + 2) addition reaction.

**Fig. 12:**
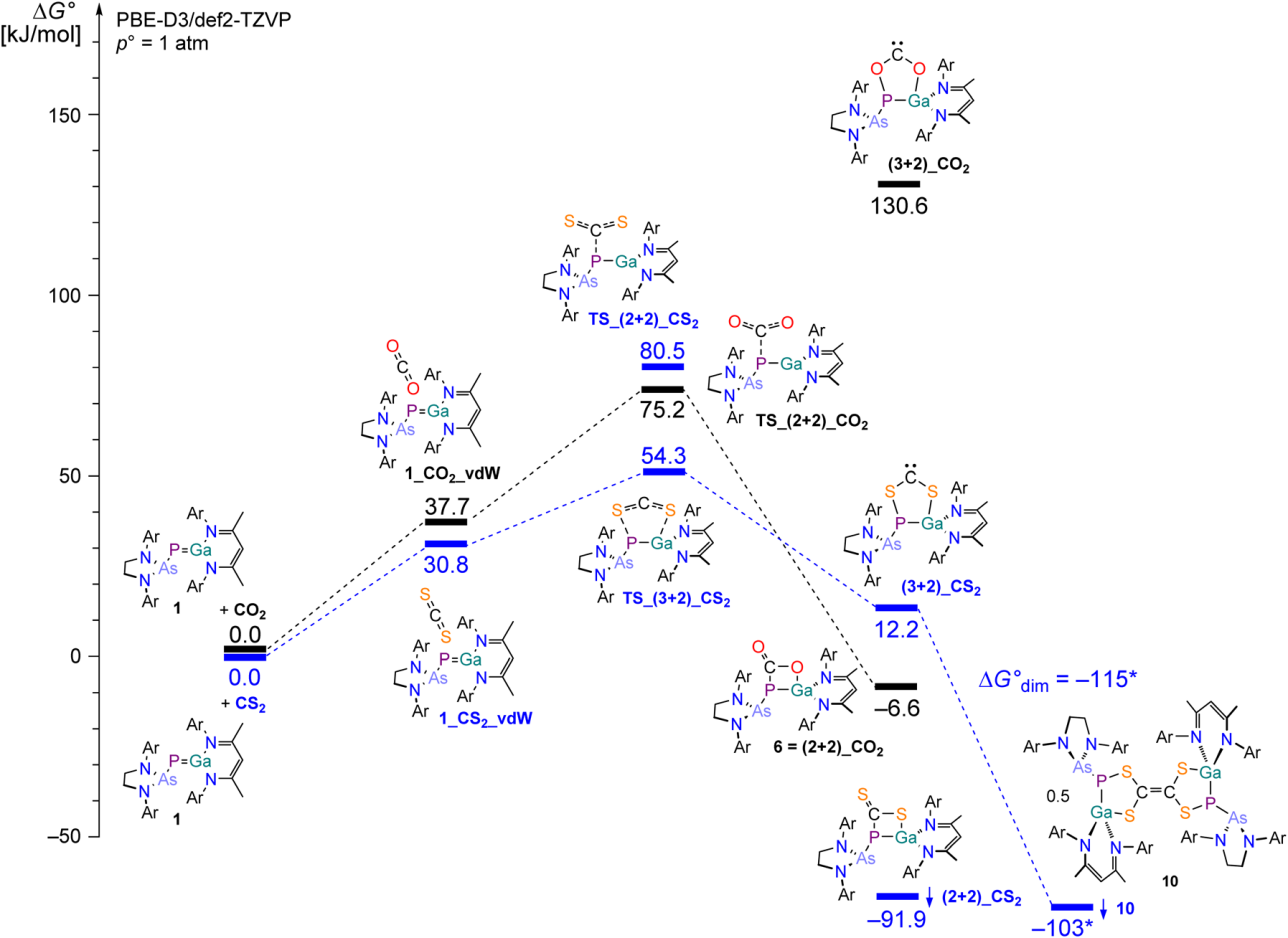
Schematic view of the energy diagram of the (2 + 2) or (2 + 3) addition reactions of 1 and CO_2_ or CS_2_, respectively, at the PBE-D3/def2-TZVP level of theory. *For computational reasons, the dimerization energy Δ*G*_dim_° formally yielding 0.5 eq. 10 was calculated for a truncated model system 10_Ph (for computational details, see ESI‡).

The reaction of 1 and CO_2_ proceeds initially *via* a metastable van-der-Waals adduct (Δ*G*° = +37.7 kJ mol^−1^, see Fig. 12). The experimentally observed, formal (2 + 2) addition product is the thermodynamically favored reaction product (−6.6 kJ mol^−1^), with an activation barrier of +75.2 kJ mol^−1^ with respect to the starting materials. The transition state resembles a R–PCO_2_^−^ formate-like structure as the product of a nucleophilic attack of the P1 lone pair at the C1 atom of the CO_2_ molecule. The formation of a (3 + 2) carbene-type intermediate of 1 and CO_2_ was calculated to be significantly energetically uphill (+130.6 kJ mol^−1^). This is in accordance with the experimental observation of the formation of the (2 + 2) product 6 in solution (*vide ante*), which then further reacts to yield either product 7 or product 8.

The reaction of 1 and CS_2_ also leads to a metastable van-der-Waals adduct (+30.8 kJ mol^−1^) in the first instance. However, even though the formal (2 + 2) addition product would be the thermodynamically favored product (−91.9 kJ mol^−1^), the R–PCS_2_^−^ thioformate-like transition state (+80.5 kJ mol^−1^, see Scheme S17‡) is energetically unfavorable. The formation of a carbene-type product *via* a (3 + 2) addition has a significantly lower activation barrier (+54.3 kJ mol^−1^) and is therefore the dominant reaction pathway, eventually leading to the experimentally observed dimerization product. We calculated the dimerization process of the (3 + 2) carbene-type intermediate yielding compound 10 by using a model system with phenyl-substituents instead of Dipp substituents. These calculations suggest that the dimerization reaction is a highly exergonic process (Δ*G*_dim_° = −115.3 kJ mol^−1^ for the reaction (3 + 2)_CS_2_ → ½ 10). Thus, the dimerization reaction yielding 10 is an irreversible process.

For the conversion of 1 with COS, one potential product of a (3 + 2) addition reactions was calculated to be thermodynamically uphill (65.5 kJ mol^−1^), whilst the formation of the formal (2 + 2) addition products was found to be exergonic (−15.9 kJ mol^−1^ and −64.6 kJ mol^−1^). This indicates that the formation of experimentally characterized compound 11 proceeds *via* a (2 + 2) intermediate, as analogously observed for the conversion of 1 and CO_2_. In this case, no further mechanistic studies were conducted.

## Conclusion

We show that altering the pendant functionality of a phosphagallene has a profound effect on the reactivity of the P=Ga double bond. The previously reported phosphanyl-phosphagallene exhibits frustrated Lewis pair reactivity towards heteroallenes. By contrast, tempering the reactivity of the pendant Lewis base by replacement of the phosphanyl group for an arsanyl group unlocks the reactivity of the P=Ga bond. We have shown this through cyclization reactions with species such as azides, carbodiimides and isocyanates. More interesting are reactions occur with CO_2_, CS_2_ and COS. In the case of carbon monoxide, a deoxygenation reaction was observed cleaving one of the C=O bonds. Such reactivity is very rare amongst compounds of the main-group elements. COS reacts in an analogous manner, however in this case, the weaker C=S bond is cleaved. The reactivity towards CS_2_ is, by contrast, much more different, proceeding *via* a carbene intermediate that dimerizes to give a molecule with a tetrathio-oxalate core. These studies serve to expand the known reactivity of a burgeoning class of molecules possessing heteroatomic double bonds between the heavier main-group elements.

## Experimental

All manipulations were carried out under oxygen- and moisture free conditions under argon or nitrogen atmosphere using standard Schlenk or drybox techniques. The reported products are (mostly very) sensitive towards oxygen and moisture. All starting materials were produced or purified as stated in the ESI.‡ Unless stated otherwise, the experiments were carried out at room temperature (approx. 23 °C) and the removal of solvents was realized *in vacuo* (1 × 10^−3^ mbar) at room temperature. Further information on experimental procedures, data acquisition and processing, purification of solvents and starting materials, on computational investigations, additional spectroscopic data and a full set of analytical data for each compound can be found in the ESI.‡

### Synthesis of 1

Toluene (7 mL) was added to a mixture of Ga(NacNac) (1.05 eq., 80.0 mg, 0.164 mmol) and [As]PCO (80.0 mg, 0.156 mmol) at ambient temperature (25 °C). The reaction mixture turned dark red within seconds and rapid effervescence was observed (caution: the reaction evolves of CO). The mixture was stirred at ambient temperature for one hour, after which all volatile components were removed *in vacuo* (1 × 10^−3^ mbar, 25 °C). The dark red solid was extracted with *n*-pentane (10 mL) and the resulting slightly turbid solution filtered. The clear, red filtrate was concentrated to a volume of approx. 3 mL *in vacuo* using a warm water bath (1 × 10^−3^ mbar, 35 °C). The product crystallizes in form of large red blocks from a concentrated *n*-pentane solution overnight (these crystals are also suitable for X-ray diffraction). The supernatant was removed with a syringe and discarded. The resulting crystalline product 1 was dried under a dynamic vacuum using a warm water bath for three hours (1 × 10^−3^ mbar, 45 °C). Yield: 92.0 mg, 0.0947 mmol, 60.7%.

EA for C_55_H_79_AsGaN_4_P (M.W. = 971.88 g mol^−1^) calcd (found) in %: C 67.97 (67.50), H 8.19 (8.69), N 5.76 (5.66). ^31^P{^1^H} NMR (C_6_D_6_, 298.0 K, 202.37 MHz): *δ* (ppm) −72.2 (m; AsPGa). ^1^H NMR (C_6_D_6_, 298.0 K, 499.93 MHz): *δ* (ppm) 7.16–7.25 (m, 8H; ArC*H*), 7.05 (d, ^3^*J*_H–H_ = 7.6 Hz, 4H; ArC*H*), 4.86 (s, 1H; NacNac γ-H), 4.32–4.36 (m, 2H; [SP]Dipp{C*H*(CH_3_)_2_}), 3.95–4.03 (m, 2H; (NC*H*_2_)_2_), 3.56 (sept, ^3^*J*_H–H_ = 6.8 Hz, 2H; [SP]{C*H*(CH_3_)_2_}), 3.27–3.36 (m, 2H; NC*H*_2_), 2.89 (sept, ^3^*J*_H–H_ = 6.8 Hz, 4H, NacnNac{C*H*(CH_3_)_2_}), 1.35–1.42 (m, 18H; NacNacC*H*_3_ and Dipp{CH(C*H*_3_)_2_}), 1.31 (d, *J* = 6.9 Hz, 6H; Dipp{CH(C*H*_3_)_2_}), 1.29 (d, *J* = 6.9 Hz, 6H; Dipp{CH(C*H*_3_)_2_}), 1.02 (d, ^3^*J*_H–H_ = 6.7 Hz, 12H; Dipp{CH(C*H*_3_)_2_}), 0.98 (d, ^3^*J*_H–H_ = 6.9 Hz, 12H; Dipp{CH(C*H*_3_)_2_}).

### Synthesis of 2

1 (35 mg, 0.036 mmol) was dissolved in C_6_H_6_ (0.7 mL) in a J. Young NMR tube. *Tert*-butyl azide (1.05 eq., 3.70 mg, 0.0370 mmol) was added to the NMR tube causing the solution to quickly turn orange. All volatile components were removed *in vacuo* (1 × 10^−3^ mbar, 25 °C) and the orange solid extracted with *n*-hexane. The solution was filtered into a small vial and kept at −30 °C overnight resulting in the formation of orange crystals of 2. The supernatant was removed, discarded and the product dried under a dynamic vacuum for three hours (1 × 10^−3^ mbar, 25 °C). Yield: 23.2 mg, 0.0217 mmol, 61.0%. Crystals suitable for X-ray diffraction were grown from a mixture of *n*-hexane/benzene (1 : 1) at room temperature. Due to the (partly) reversible adduct formation with ^*t*^BuN_3_ at high temperatures, it was not possible to obtain an accurate elemental analysis of 2.

EA for C_59_H_88_AsGaN_7_P (M.W. = 1071.01 g mol^−1^) calcd (found) in %: C 66.17 (67.85), H 8.28 (8.00), N 7.00 (7.20). ^31^P{^1^H} NMR (C_6_D_6_, 298.0 K, 202.38 MHz): *δ* (ppm) −31.6 (s; AsPGa). ^1^H NMR (C_6_D_6_, 298.0 K, 499.98 MHz): *δ* (ppm) 7.16–7.20 (m, 2H; ArC*H*), 7.12–7.15 (m, 2H; ArC*H*), 7.02–7.08 (m, 4H; ArC*H*), 6.86–6.97 (m, 4H; ArC*H*), 4.88 (s, 1H; NacNac γ-H), 4.50–4.65 (m, 2H; Dipp{C*H*(CH_3_)_2_}), 4.15–4.30 (m, 2H; NC*H*_2_), 3.30–3.50 (m, 4H; NC*H*_2_ and Dipp{C*H*(CH_3_)_2_}), 3.15–3.38 (m, 2H; Dipp{C*H*(CH_3_)_2_}), 2.80–2.95 (m, 2H; Dipp{C*H*(CH_3_)_2_}), 1.60 (s, 9H; ^*t*^BuCH_3_), 1.29 (s, 6H; NacNacC*H*_3_), 1.25 (d, ^3^*J*_H–H_ = 6.9 Hz, 6H; Dipp{CH(C*H*_3_)_2_}), 1.18–1.23 (m, 12H; Dipp{CH(C*H*_3_)_2_}), 1.13–1.17 (m, 6H; Dipp{CH(C*H*_3_)_2_}), 1.02 (br. d, ^3^*J*_H–H_ = 6.7 Hz, 12H; Dipp{CH(C*H*_3_)_2_}), 0.74 (br. s, 6H; Dipp{CH(C*H*_3_)_2_}), 0.60 (br. d, ^3^*J*_H–H_ = 6.3 Hz, 6H; Dipp{CH(C*H*_3_)_2_}).

### Synthesis of 3

1 (34.0 mg, 0.0350 mmol) was dissolved in C_6_H_6_ (0.7 mL) in a J. Young NMR tube. *N*,*N*′-Dicyclohexylcarbodiimide (1.05 eq., 7.57 mg, 0.0370 mmol) was added to the NMR tube, after which the solution quickly turned yellow. The solution was filtered into a small vial affording colourless crystals of 3 within minutes. The filtration needs to be carried out quickly, otherwise the product can precipitate in the filter. The supernatant was removed, discarded and the resulting colourless crystalline solid was washed with small amounts of cold *n*-hexane (3 × 0.5 mL, −30 °C). The resulting crystalline solid was dried *in vacuo* for three hours (1 × 10^−3^ mbar, 25 °C). Yield: 9.70 mg, 0.008 mmol, 23.5%.

EA for C_68_H_101_AsGaN_6_P (M.W. = 1178.21 g mol^−1^) calcd (found) in %: C 69.32 (70.27), H 8.64 (8.18), N 5.92 (6.79). ^31^P{^1^H} NMR (THF-d_8_, 298.0 K, 243.05 MHz): *δ* (ppm) −27.6 (s; AsPGa). ^1^H NMR (THF-d_8_, 298.0 K, 600.42 MHz): *δ* (ppm) 7.08–7.13 (m, 6H; ArC*H*), 7.03–7.07 (m, 4H; ArC*H*), 7.00–7.03 (br. s, 2H; ArC*H*), 6.93–6.98 (m, 4H; ArC*H*), 5.32 (s, 1H; NacNac γ-H), 3.90–3.98 (m, 2H; NC*H*_2_), 3.70–3.78 (m, 2H; Dipp{C*H*(CH_3_)_2_}), 3.40–3.47 (m, 1H; CyC*H*), 3.25–3.35 (m, 6H; NC*H*_2_ and Dipp{C*H*(CH_3_)_2_}), 3.18–3.25 (m, 1H; CyC*H*), 2.89 (sept, ^3^*J*_H–H_ = 6.9 Hz, 2H; Dipp{C*H*(CH_3_)_2_}), 2.42 (br. d, ^3^*J*_H–H_ = 10.9 Hz, 2H; CyC*H*_2_), 1.88 (br. d, ^3^*J*_H–H_ = 12.9 Hz, 2H; CyC*H*_2_), 1.65 (br. s, 8H; CyC*H*_2_ and NacNacC*H*_3_), 1.53–1.62 (m, 2H; CyC*H*_2_), 1.25–1.38 (m, 10H; CyC*H*_2_), 1.09 (br. d, ^3^*J*_H–H_ = 6.8 Hz, ^3^*J*_H–H_ = 2.3 Hz, 12H; Dipp{CH(C*H*_3_)_2_}), 1.05 (br. s, 3H; Dipp{CH(C*H*_3_)_2_}), 1.04 (br. d, ^3^*J*_H–H_ = 3.8 Hz, 6H; Dipp{CH(C*H*_3_)_2_}), 1.02 (br. d, ^3^*J*_H–H_ = 4.5 Hz, 6H; Dipp{CH(C*H*_3_)_2_}), 1.00 (br. s, 6H; Dipp{CH(C*H*_3_)_2_}), 0.99 (br. s, 3H; Dipp{CH(C*H*_3_)_2_}), 0.93–0.98 (m, 2H; CyC*H*_2_), 0.85 (br. d, ^3^*J*_H–H_ = 6.7 Hz, 6H; Dipp{CH(C*H*_3_)_2_}), 0.68 (br. d, ^3^*J*_H–H_ = 6.7 Hz, 6H; Dipp{CH(C*H*_3_)_2_}).

### Synthesis of 4

1 (52.0 mg, 0.0540 mmol) was dissolved in C_6_H_6_ (0.7 mL) in a J. Young NMR tube. 2,4,6-Trimethylphenyl isocyanate (1.06 eq., 9.10 mg, 0.0560 mmol) was added to the NMR tube, and the resulting reaction mixture turned yellow. After the addition, all volatile components were removed *in vacuo* (1 × 10^−3^ mbar, 25 °C) and the yellow solid was extracted with *n*-hexane. The solution was filtered into a small vial. Storage of the solution at −30 °C overnight afforded yellow crystals of 4. The supernatant was removed and discarded, and the solid washed with small amounts of cold *n*-hexane (3 × 0.3 mL, −30 °C). The resulting yellow product was dried under a dynamic vacuum for three hours (1 × 10^−3^ mbar, 25 °C). Yield: 33.7 mg, 0.030 mmol, 55.5%. Crystals suitable for X-ray diffraction were grown from a mixture of *n*-hexane/benzene (1 : 1) at room temperature.

EA for C_65_H_90_AsGaN_5_OP (M.W. = 1133.08 g mol^−1^) calcd (found) in %: C 68.90 (69.55), H 8.01 (7.91), N 6.18 (6.29). ^31^P{^1^H} NMR (C_6_D_6_, 298.0 K, 243.05 MHz): *δ* (ppm) −34.5 (s; As*P*Ga). ^1^H NMR (C_6_D_6_, 298.0 K, 600.42 MHz): *δ* (ppm) 7.20–7.35 (m, 6H; ArC*H*), 7.02–7.15 (m, 3H; ArC*H*), 6.95–7.01 (br. s, 2H; Mes ArC*H*), 6.85–6.90 (br. s, 1H; Mes ArC*H*), 6.59 (s, 1H; Mes ArC*H*), 4.66 (s, 1H; NacNac γ-H), 4.08 (br. s, 1H; Dipp{C*H*(CH_3_)_2_}), 3.94 (br. s, 1H; NC*H*_2_), 3.85 (br. s, 1H; Dipp{C*H*(CH_3_)_2_}), 3.74 (br. s, 2H; Dipp{C*H*(CH_3_)_2_}), 3.65 (br. s, 1H; NC*H*_2_), 3.48 (br. s, 2H; NC*H*_2_ and Dipp{C*H*(CH_3_)_2_}), 3.24 (br. s, 1H; NC*H*_2_), 2.87–3.05 (m, 2H; Dipp{C*H*(CH_3_)_2_}), 2.79 (br. s, 1H; Dipp{C*H*(CH_3_)_2_}), 2.14 (s, 3H; MesC*H*_3_); 1.88 (br. s, 3H; MesC*H*_3_), 1.15–1.53 (m, 42H; MesC*H*_3_ and/or NacNacC*H*_3_ and/or Dipp{CH(C*H*_3_)_2_}), 0.90–1.05 (m, 9H; MesC*H*_3_ and/or NacNacC*H*_3_ and/or Dipp{CH(C*H*_3_)_2_}), 0.73 (br. s, 3H; MesC*H*_3_ and/or NacNacC*H*_3_ and/or Dipp{CH(C*H*_3_)_2_}), 0.50 (br. s, 3H; MesC*H*_3_ and/or NacNacC*H*_3_ and/or Dipp{CH(C*H*_3_)_2_}).

### Synthesis of 5

1 (58.0 mg, 0.060 mmol) was dissolved in C_6_H_6_ (0.7 mL) in a J. Young NMR tube. Benzaldehyde (1.35 eq., 8.60 mg, 0.0810 mmol) was added to the NMR tube causing the solution to quickly turn yellow. After addition, all volatile components were removed *in vacuo* (1 × 10^−3^ mbar, 25 °C) and the yellow solid extracted with *n*-hexane. The solution was filtered into a small vial. After storage at −30 °C overnight, yellow crystals of 5 were obtained. The supernatant was removed and discarded, and the product was dried *in vacuo* for three hours (1 × 10^−3^ mbar, 25 °C). Yield: 22.5 mg, 0.0210 mmol, 35.0%. Crystals suitable for X-ray diffraction were grown from a mixture of *n*-hexane/benzene (1 : 1) at room temperature.

EA for C_62_H_85_AsGaN_4_OP (M.W. = 1078.00 g mol^−1^) calcd (found) in %: C 69.08 (69.27), H 7.95 (7.63), N 5.20 (5.28). ^31^P{^1^H} NMR (C_6_D_6_, 298.0 K, 202.38 MHz): *δ* (ppm) 67.6 (s, As*P*Ga). ^1^H NMR (C_6_D_6_, 298.0 K, 499.93 MHz): *δ* (ppm) 7.32–7.37 (m, 2H; ArC*H*), 7.20–7.28 (m, 3H; ArC*H*), 7.17–7.19 (m, 2H; ArC*H*), 7.08–7.14 (m, 5H; ArC*H*), 6.70–6.75 (m, 1H; Ph ArC*H*), 6.61 (t, ^3^*J*_H–H_ = 7.6 Hz, 2H; Ph ArC*H*), 5.96 (br. d, ^3^*J* = 7.1 Hz, 2H; Ph ArC*H*); 5.59 (d, ^3^*J*_P–H_ = 34 Hz, 1H; PhC*H*O); 4.81 (s, 1H; NacNac γ-H), 4.40 (dq, ^3^*J*_H–H_ = 12.8 Hz, *J* = 6.6 Hz, 1H; Dipp{C*H*(CH_3_)_2_}), 4.23 (td, ^3^*J*_H–H_ = 9.7 Hz, *J* = 5.7 Hz, 1H; NC*H*_2_), 3.91 (sept, ^3^*J*_H–H_ = 6.9, 1H; Dipp{C*H*(CH_3_)_2_}), 3.57 (sept, ^3^*J*_H–H_ = 6.7, 1H; Dipp{C*H*(CH_3_)_2_}), 3.30–3.50 (m, 5H; NC*H*_2_ and Dipp{C*H*(CH_3_)_2_}), 3.20 (sept, ^3^*J*_H–H_ = 6.8, 1H; Dipp{C*H*(CH_3_)_2_}), 3.09–3.17 (m, 1H; NC*H*_2_); 2.89 (sept, ^3^*J*_H–H_ = 6.7, 1H; Dipp{C*H*(CH_3_)_2_}), 1.94 (d, ^3^*J*_H–H_ = 6.7, 3H; Dipp{CH(C*H*_3_)_2_}), 1.59 (s, 3H; Dipp{CH(C*H*_3_)_2_}), 1.52 (s, 3H; Dipp{CH(C*H*_3_)_2_}), 1.45–1.49 (m, 6H; Dipp{CH(C*H*_3_)_2_}), 1.32–1.40 (m, 12H; Dipp{CH(C*H*_3_)_2_}), 1.20–1.28 (m, 12H; NacNacC*H*_3_ and Dipp{CH(C*H*_3_)_2_}), 1.19 (d, ^3^*J*_H–H_ = 6.9, 3H; Dipp{CH(C*H*_3_)_2_}), 1.10–1.16 (m, 6H; Dipp{CH(C*H*_3_)_2_}), 0.93 (d, ^3^*J*_H–H_ = 6.7, 3H; Dipp{CH(C*H*_3_)_2_}), 0.39 (d, ^3^*J*_H–H_ = 6.7, 3H; Dipp{CH(C*H*_3_)_2_}).

### Synthesis of 7

1 (50.0 mg, 0.0520 mmol) was dissolved in C_6_H_6_ (0.7 ml) in a J. Young NMR tube. Dry CO_2_ (2 bar) was added to the NMR tube, which was then sealed and stored at ambient temperature overnight (approx. 16 hours). Reaction progress was monitored using ^31^P{^1^H} NMR spectroscopy. When conversion to the main product in the ^31^P NMR spectrum (−9.0 ppm) was complete, all volatiles were removed *in vacuo* (1 × 10^−3^ mbar, 25 °C). The resulting colourless precipitate was extracted with *n*-pentane (0.5 mL). The resulting solution was filtered into a small vial and kept at −30 °C overnight affording small, colourless crystals of 7. The supernatant was removed with a syringe and discarded. The resulting colourless crystals were dried *in vacuo* for three hours (1 × 10^−3^ mbar, 25 °C). Yield: 25.6 mg, 0.0240 mmol, 47.0%.

EA for C_57_H_79_AsGaN_4_O_4_P (M.W. = 1059.9 g mol^−1^) calcd (found) in %: C 64.59 (65.64), H 7.51 (6.85), N 5.29 (5.07). ^31^P{^1^H} NMR (C_6_D_6_, 298.0 K, 202.37 MHz): *δ* (ppm) −9.0 (s; As*P*(CO_2_)_2_). ^1^H NMR (C_6_D_6_, 298.0 K, 499.93 MHz): *δ* (ppm) 7.17–7.19 (m, 1H; ArC*H*), 6.96–7.00 (m, 7H; ArC*H*), 7.03–7.14 (m, 4H; ArC*H*), 4.79 (s, 1H; NacNac γ-H), 3.88–3.98 (m, 2H; NC*H*_2_), 3.65–3.73 (m, 2H; Dipp{C*H*(CH_3_)_2_}), 3.52–3.62 (m, 2H; Dipp{C*H*(CH_3_)_2_}), 3.15–3.25 (m, 2H; NC*H*_2_), 3.65–3.73 (sept, ^3^*J*_H–H_ = 6.8 Hz, 4H; Dipp{C*H*(CH_3_)_2_}), 1.44 (s, 6H; NacNacC*H*_3_), 1.32 (d, ^3^*J*_H–H_ = 6.7 Hz, 12H; Dipp{CH(C*H*_3_)_2_}), 1.24 (d, ^3^*J*_H–H_ = 6.9 Hz, 12H; Dipp{CH(C*H*_3_)_2_}), 1.22 (br. d, ^3^*J*_H–H_ = 6.9 Hz, 6H; Dipp{CH(C*H*_3_)_2_}), 1.04 (d, ^3^*J*_H–H_ = 6.7 Hz, 6H; Dipp{CH(C*H*_3_)_2_}), 0.96 (d, ^3^*J*_H–H_ = 6.9 Hz, 12H; Dipp{CH(C*H*_3_)_2_}).

### Synthesis of 8

1 (55.0 mg, 0.0570 mmol) was dissolved in C_6_H_6_ (0.7 ml) in a J. Young NMR tube. Dry CO_2_ (2 bar) was added to the NMR tube, which was sealed and heated to 80 °C for approx. 3 days in an oil bath. The reaction progress was monitored by ^31^P NMR spectroscopy. When the major species formed corresponded to a singlet at −371.1 ppm in the ^31^P{^1^H} NMR spectrum, all volatiles were removed *in vacuo* (1 × 10^−3^ mbar, 25 °C) and the colourless precipitate was extracted with *n*-hexane (0.5 mL). The resulting solution was filtered into a small vial and kept at −30 °C overnight affording small, colourless crystals of 8. The supernatant was removed with a syringe and discarded. The resulting colourless crystals were dried *in vacuo* for three hours (1 × 10^−3^ mbar, 25 °C). Yield: 18.9 mg, 0.0190 mmol, 32.9%. Notice: due to very broad ^1^H NMR signals at 298 K (see Fig. S7‡), the ^1^H NMR spectrum was recollected at 343 K, where the signals are sharper. In the ^31^P NMR spectrum at 298 K, no signal broadening was observed.

EA for C_56_H_79_AsGaN_4_O_2_P (M.W. = 1015.89 g mol^−1^) calcd (found) in %: C 66.21 (66.00), H 7.84 (7.34), N 5.52 (5.08). ^31^P{^1^H} NMR (C_6_D_6_, 298.0 K, 243.05 MHz): *δ* (ppm) −371.1 (s; Ga*P*CO). ^1^H NMR (C_6_D_6_, 343.0 K, 499.93 MHz): *δ* (ppm) 6.98–7.15 (m, 12H; ArC*H*), 4.89 (s, 1H; NacNac γ-H), 4.03–4.25 (m, 4H; Dipp{C*H*(CH_3_)_2_}), 3.71–3.85 (m, 2H; NC*H*_2_), 3.37–3.51 (m, 4H; Dipp{C*H*(CH_3_)_2_}), 3.05–3.19 (m, 2H; NC*H*_2_), 2.14 (br. s, 3H; Dipp{CH(C*H*_3_)_2_} and NacNacC*H*_3_), 1.53 (br. s; 6H; Dipp{CH(C*H*_3_)_2_} and NacNacC*H*_3_), 0.98–1.50 (45H; Dipp{CH(C*H*_3_)_2_} and NacNacC*H*_3_).

### Synthesis of 9

Compound B (33 mg, 0.036 mmol) was dissolved in C_6_H_6_ (0.7 ml) in a J. Young NMR tube. CS_2_ (2.1 μL, 2.7 mg, 0.036 mmol) was added to the NMR tube at ambient temperature (25 °C) resulting in an immediate colour change of the reaction mixture from red to dark purple. The tube was kept at ambient temperature for one hour after which all volatile components were removed *in vacuo* (1 × 10^−3^ mbar, 25 °C). The purple solid was extracted with *n*-hexane (0.5 mL) and the resulting solution was filtered into a small vial and kept at −30 °C overnight to give purple crystals of 9. The supernatant was removed with a syringe and discarded. The resulting purple crystals were dried *in vacuo* for three hours (1 × 10^−3^ mbar, 25 °C). Yield: 9.2 mg, 0.0090 mmol, 26%.

EA for C_56_H_79_GaN_4_P_2_S_2_ (M.W. = 1004.07 g mol^−1^) calcd (found) in %: C 66.99 (65.81), H 7.93 (6.99), N 5.58 (4.77). ^31^P NMR (C_6_D_6_, 298.0 K, 243.05 MHz): *δ* (ppm) 102.7 (d, ^1^*J*_P–P_ = 614.0 Hz; *P*PGa), −287.3 (d, ^1^*J*_P–P_ = 614.0 Hz; P*P*Ga). ^1^H NMR (C_6_D_6_, 298.0 K, 600.42 MHz): *δ* (ppm) 7.14–7.19 (m, 2H; ArC*H*), 7.04–7.10 (m, 4H; ArC*H*), 6.93–6.98 (m, 4H; ArC*H*), 6.88–6.92 (m, 2H; ArC*H*), 4.66 (s, 1H; NacNac γ-H), 3.85–3.93 (m, 2H; NC*H*_2_), 3.78 (sept, ^3^*J*_H–H_ = 6.9 Hz, 2H; Dipp{C*H*(CH_3_)_2_}), 3.72 (sept, ^3^*J*_H–H_ = 6.8 Hz, 2H; Dipp{C*H*(CH_3_)_2_}), 3.20–3.30 (m, 2H; NC*H*_2_), 3.15 (sept, ^3^*J*_H–H_ = 6.8 Hz, 2H; Dipp{C*H*(CH_3_)_2_}), 3.05 (sept, ^3^*J*_H–H_ = 6.8 Hz, 2H; Dipp{C*H*(CH_3_)_2_}), 1.38 (s, 6H; NacNacC*H*_3_), 1.36 (d, ^3^*J*_H–H_ = 6.7 Hz, 6H; Dipp{CH(C*H*_3_)_2_}); 1.12–1.17 (m, 18H; Dipp{CH(C*H*_3_)_2_}), 1.04–1.10 (m, 12H; Dipp{CH(C*H*_3_)_2_}), 0.99 (d, ^3^*J*_H–H_ = 6.9 Hz, 6H; Dipp{CH(C*H*_3_)_2_}), 0.92 (d, ^3^*J*_H–H_ = 6.7 Hz, 6H; Dipp{CH(C*H*_3_)_2_}).

### Synthesis of 10

1 (50.0 mg, 0.0520 mmol) was dissolved in C_6_H_6_ (0.7 ml) in a J. Young NMR tube. An excess of CS_2_ (1 drop) was added to the NMR tube at ambient temperature (25 °C) after which the colour of the reaction mixture changed from dark red to orange. The tube was kept at ambient temperature for one hour and afterwards all volatile components were removed *in vacuo* (1 × 10^−3^ mbar, 25 °C). The orange precipitate was extracted with *n*-hexane (0.5 mL) and the resulting slightly turbid solution was filtered into a small vial. Storage of this solution at ambient temperature overnight afforded orange crystals of 10 (suitable for X-ray diffraction). The supernatant was removed with a syringe and discarded. The resulting orange crystals were dried *in vacuo* for three hours (1 × 10^−3^ mbar, 25 °C). Yield: 29.4 mg, 0.0140 mmol, 54.5% (of dimer, max. yield is 100%).

EA for C_112_H_158_As_2_Ga_2_N_8_P_2_S_4_ (M.W. = 2096.04 g mol^−1^) calcd (found) in %: C 64.18 (64.42), H 7.60 (8.23), N 5.35 (4.93). ^31^P NMR (C_6_D_6_, 298.0 K, 243.05 MHz): *δ* (ppm) −25.9 (s; As*P*(S)Ga), −52.5 (s; As*P*(S)Ga). ^1^H NMR (C_6_D_6_, 298.0 K, 600.42 MHz): *δ* (ppm) 7.25–7.38 (m, 13H; ArC*H*), 7.19 (^3^*J*_H–H_ = 7.5 Hz, 1H; ArC*H*), 7.12–7.15 (m, 2H; ArC*H*), 7.08 (^3^*J*_H–H_ = 7.5 Hz, 1H; ArC*H*), 6.94–7.05 (m, 6H; ArC*H*), 6.79 (^3^*J*_H–H_ = 7.3 Hz, 1H; ArC*H*), 4.80 (s, 1H; NacNac γ-H), 4.67 (s, 1H; NacNac γ-H), 4.30–4.40 (m, 1H; NC*H*_2_), 4.20–4.28 (m, 1H; NC*H*_2_), 4.02–4.11 (m, 2H; Dipp{C*H*(CH_3_)_2_}), 3.85–4.01 (m, 4H; Dipp{C*H*(CH_3_)_2_} and NC*H*_2_), 3.60–3.65 (m, 1H; Dipp{C*H*(CH_3_)_2_}), 3.53–3.58 (m, 2H; Dipp{C*H*(CH_3_)_2_} and NC*H*_2_), 3.40–3.50 (m, 4H; Dipp{C*H*(CH_3_)_2_} and NC*H*_2_), 3.30–3.40 (m, 1H; NC*H*_2_), 3.20–3.30 (m, 2H; Dipp{C*H*(CH_3_)_2_}), 3.05–3.10 (m, 3H; Dipp{C*H*(CH_3_)_2_}), 2.90–3.00 (m, 3H; Dipp{C*H*(CH_3_)_2_} and NC*H*_2_), 1.86 (d, ^3^*J*_H–H_ = 6.5 Hz, 3H; NacNacC*H*_3_), 1.81 (d, ^3^*J*_H–H_ = 6.5 Hz, 3H; NacNacC*H*_3_), 1.68 (d, ^3^*J*_H–H_ = 6.7 Hz, 3H; Dipp{CH(C*H*_3_)_2_}), 1.60–1.64 (m, 6H; NacNacC*H*_3_), 1.59 (d, ^3^*J*_H–H_ = 6.9 Hz, 3H; Dipp{CH(C*H*_3_)_2_}), 1.52–1.56 (m, 6H; Dipp{CH(C*H*_3_)_2_}), 1.44–1.48 (m, 21H; Dipp{CH(C*H*_3_)_2_}), 1.40 (d, ^3^*J*_H–H_ = 6.7 Hz, 3H; Dipp{CH(C*H*_3_)_2_}), 1.32–1.37 (m, 6H; Dipp{CH(C*H*_3_)_2_}), 1.30 (d, ^3^*J*_H–H_ = 6.9 Hz, 3H; Dipp{CH(C*H*_3_)_2_}), 1.27 (d, ^3^*J*_H–H_ = 6.7 Hz, 3H; Dipp{CH(C*H*_3_)_2_}), 1.52–1.56 (m, 9H; Dipp{CH(C*H*_3_)_2_}), 1.14–1.18 (m, 6H; Dipp{CH(C*H*_3_)_2_}), 1.08–1.12 (m, 9H; Dipp{CH(C*H*_3_)_2_}), 0.99 (d, ^3^*J*_H–H_ = 6.7 Hz, 3H; Dipp{CH(C*H*_3_)_2_}), 0.97 (d, ^3^*J*_H–H_ = 6.7 Hz, 3H; Dipp{CH(C*H*_3_)_2_}), 0.94 (d, ^3^*J*_H–H_ = 6.7 Hz, 3H; Dipp{CH(C*H*_3_)_2_}), 0.92 (d, ^3^*J*_H–H_ = 6.7 Hz, 3H; Dipp{CH(C*H*_3_)_2_}), 0.61 (d, ^3^*J*_H–H_ = 6.7 Hz, 3H; Dipp{CH(C*H*_3_)_2_}), 0.40–0.47 (m, 6H; Dipp{CH(C*H*_3_)_2_}), 0.21 (d, ^3^*J*_H–H_ = 6.5 Hz, 3H; Dipp{CH(C*H*_3_)_2_}).

### Synthesis of 11

1 (60.0 mg, 0.0610 mmol) was dissolved in C_6_H_6_ (0.7 mL) in a J. Young NMR tube. Dry COS (1 bar) was added to the NMR tube, which was then sealed and stored at room temperature for approx. 1 hour. Reaction progress was monitored using ^31^P{^1^H} NMR spectroscopy. When the spectrum revealed a singlet at −371.1 ppm, the volatile components were removed *in vacuo* (1 × 10^−3^ mbar, 25 °C) and the yellow precipitate was extracted with *n*-pentane/toluene (0.3 mL/0.3 mL). The resulting solution was filtered into a small vial and kept at room temperature overnight affording small, colourless crystals of 11. The supernatant was removed with a syringe, discarded and the resulting colourless crystals were dried *in vacuo* for three hours (1 × 10^−3^ mbar, 25 °C). Yield: 27.3 mg, 0.0270 mmol, 43.4%.

EA for C_56_H_79_AsGaN_4_OSP (M.W. = 1031.95 g mol^−1^) calcd (found) in %: C 65.18 (65.63), H 7.72 (7.76), N 5.43 (5.14). ^31^P NMR (C_6_D_6_, 298.2 K, 242.95 MHz): *δ* (ppm) −365.4 (s, Ga*P*CO). ^1^H NMR (C_6_D_6_, 298.2 K, 600.16 MHz): *δ* (ppm) 7.17–7.21 (m, 2H; ArC*H*), 7.08–7.11 (m, 3H; ArC*H*), 7.00–7.05 (m, 5H; ArC*H*), 6.96–6.99 (m, 2H; ArC*H*), 4.83 (s, 1H; NacNac γ-H), 3.81–3.90 (m, 2H; Dipp{C*H*(CH_3_)_2_}), 3.65–3.75 (m, 4H; Dipp{C*H*(CH_3_)_2_} and NC*H*_2_), 3.50–3.60 (m, 2H; Dipp{C*H*(CH_3_)_2_}), 3.15–3.25 (m, 4H; Dipp{C*H*(CH_3_)_2_} and NC*H*_2_); 1.54 (d, 6H, ^3^*J*_H–H_ = 6.9 Hz; Dipp{CH(C*H*_3_)_2_}), 1.39 (s, 6H; NacNacC*H*_3_), 1.54 (d, 6H, ^3^*J*_H–H_ = 6.9 Hz; Dipp{CH(C*H*_3_)_2_}), 1.42 (d, 6H, ^3^*J*_H–H_ = 6.9 Hz; Dipp{CH(C*H*_3_)_2_}), 1.26 (d, 6H, ^3^*J*_H–H_ = 6.5 Hz, Dipp{CH(C*H*_3_)_2_}), 1.23 (d, 6H, ^3^*J*_H–H_ = 6.9 Hz, Dipp{CH(C*H*_3_)_2_}), 1.11 (d, 6H, ^3^*J*_H–H_ = 6.9 Hz; Dipp{CH(C*H*_3_)_2_}), 1.06 (d, 6H, ^3^*J*_H–H_ = 6.9 Hz; Dipp{CH(C*H*_3_)_2_}), 0.58 (d, 6H, ^3^*J*_H–H_ = 6.9 Hz; Dipp{CH(C*H*_3_)_2_}), 0.53 (d, 6H, ^3^*J*_H–H_ = 6.9 Hz; Dipp{CH(C*H*_3_)_2_}).

## Data availability

Further information on experimental procedures, data acquisition and processing, purification of solvents and starting materials, on computational investigations, additional spectroscopic data and all full set of analytical data for each compound can be found in the ESI.‡ ^80–85^

## Author contributions

L. S. S. carried out the experimental work and recorded the SCXRD data. L. S. S. and J. B. performed the computational studies. L. F. assisted with the experiments involving COS and ^13^CO_2_. M. Ernst assisted with the SCXRD measurement on compound 11. J. M. G solved the SCXRD structures. L. S. S. and J. M. G. conceptualized the project and wrote the manuscript. All authors contributed for further revision of the ESI‡ and manuscript.

## Conflicts of interest

There are no conflicts to declare.

## Supplementary Material

SC-016-D5SC00295H-s001

SC-016-D5SC00295H-s002

SC-016-D5SC00295H-s003
