# Supplementary material for: Reactivity of an arsanyl-phosphagallene: decarbonylation of CO2 and COS to form phosphaketenes
Source: Chem Sci. 2025 Mar 17;16(17):7397–410. doi: 10.1039/d5sc00295h (PMC11938936; doi:10.1039/d5sc00295h)
Supplement: SC-016-D5SC00295H-s001 [file SC-016-D5SC00295H-s001.pdf]

This journal is © The Royal Society of Chemistry 2025

Contents

1 **Experimental** 2

2 **Synthesis of compounds** 4

2.1 **Synthesis of 1** 4

2.2 **Synthesis of 2** 7

2.3 **Synthesis of 3** 10

2.4 **Synthesis of 4** 12

2.5 **Synthesis of 5** 16

2.6 **Synthesis of 7** 19

2.7 **Synthesis of 8** 21

2.8 **Synthesis of 9** 24

2.9 **Synthesis of 10** 27

2.10 **Synthesis of 11** 31

3 **Additional spectroscopic details** 34

4 **Single crystal X-ray diffraction** 36

5 **Additional computational details** 41

6 **References** 47

1 Experimental

General information. Unless otherwise stated, all reactions and product manipulations were carried out using standard Schlenk-line techniques under an inert atmosphere of argon, or in a dinitrogen filled glovebox (MBraun UNIlab glovebox maintained at <0.1 ppm H_2O and <0.1 ppm O_2). All glassware was flame-dried and cooled under argon atmosphere. Solvents were transferred using syringes or teflon cannula, which were purged with argon prior to use. *n*-Hexane (Sigma Aldrich HPLC grade), *n*-pentane (Sigma Aldrich HPLC grade), benzene (Rathburn HPLC grade) and toluene (Sigma Aldrich HPLC grade) were purified using an MBraun SPS-800 solvent system and degassed. THF (Sigma Aldrich HPLC grade) was distilled over sodium/benzophenone. C_6D_6 (Sigma Aldrich, 99.5%) was degassed and dried over sodium/benzophenone. THF-d_8 (Sigma Aldrich, 99.5%) was degassed and dried over sodium/potassium alloy. All dry solvents were stored under argon in gas-tight J. Young ampoules over activated 4 Å molecular sieves. Reactants were either obtained from commercial sources or synthesized as detailed in Table S1.

Table S1: Origin and purification of solvents and reactants.

Substance	Origin	Purification
Ga(NacNac)	synthesized[80]	used as synthesized
$[H_2CN(Dipp)]_2$AsPCO	synthesized[46]	used as synthesized
CO_2	BOC	99.8 % purity, dried over molar sieves (4 Å)
CS_2	Sigma	dried over P_4O_{10}, distilled, stored over molar sieves (4 Å) at RT
COS	Aldrich chemical company, Inc.	dried over molar sieves (3 Å)
2,3-Dimethyl-1,3-butadiene	Sigma 98%	used as received
N,N'-Dicyclohexyl-carbodiimide	Sigma 99%	used as received
2,4,6-trimethylphenyl isocyanate	Sigma	used as received
PhCHO	Sigma	purified according to literature procedure[81]
^{t}BuN$_3$	synthesized[82]	dried over molar sieves (3 Å)
**B**	synthesized[30]	used as synthesized

Please note: In the manuscript/SI, RT (room temperature) = “ambient temperature” refers to 25(2) °C.

NMR spectra. The NMR samples were prepared inside an inert atmosphere glovebox (N_2 atmosphere, see general information) in Young NMR tubes fitted with a gas-tight valve; or prepared in a Schlenk glassware using a constant Argon flow and a suba seal. NMR spectra were acquired on either a Bruker AVIII 400 MHz NMR spectrometer (^1H 400 MHz, ^{31}P 162 MHz), a Bruker AVIII 500 spectrometer (^1H 500 MHz, ^{31}P 202 MHz) or a Bruker Avance NEO 600 MHz NMR spectrometer with a broadband helium cryoprobe (^1H 600 MHz, ^{31}P 243 MHz, ^{13}C 151 MHz) or on a ECZ600 JEOL spectrometer (^1H 600 MHz, ^{31}P 243 MHz, ^{13}C 151 MHz). ^1H and ^{13}C NMR spectra were referenced to their respective solvent resonance (^1H NMR C_6D_6: δ = 7.16 ppm, THF-d_8: 1.73 ppm or 3.58 ppm; ^{13}C NMR C_6D_6: δ = 128.4 ppm, THF-d_8: 25.37 ppm or 67.57 ppm). ^{31}P NMR spectra were externally referenced to an 85% solution of H_3PO_4 in H_2O. Multiplicities are defined as (s) for singlets, (d) for doublets, (t) for triplets, (quin) for quintets, sept for septets, (m) for multiplets, and the addition of (br.) for a broad signal. NMR spectra were measured of dissolved crystalline material, which was dried in vacuo for 3 h (1×10^{-3} mbar, 25 °C) prior to measurement.

IR spectra of crystalline samples were acquired on a Varian FTS-7000 Fourier Transform Infrared Spectrometer inside of a Glovebox with a N_2 atmosphere. Due to the measurement conditions, the IR spectra might exhibit some background noise between 1700 cm^{-1} and 2200 cm^{-1}.

Elemental analyses were carried out by Elemental Analysis Services Team (London Metropolitan University, U.K.). Samples (approx. 5 mg) were submitted in flame sealed glass tubes.

If you are interested in the original data files (NMR and IR spectra), please contact us.

2 Synthesis of compounds

2.1 Synthesis of 1

Toluene (7 ml) was added to a mixture of Ga(NacNac) (1.05 eq., 80.0 mg, 0.164 mmol) and [As]PCO (80.0 mg, 0.156 mmol) at ambient temperature (25 °C). The reaction mixture turned dark red within seconds and rapid effervescence was observed (Caution: the reaction evolves of CO). The mixture was stirred at ambient temperature for one hour, after which all volatile components were removed *in vacuo* (1×10^{-3} mbar, 25 °C). The dark red solid was extracted with *n*-pentane (10 ml) and the resulting slightly turbid solution filtered. The clear, red filtrate was concentrated to a volume of approx. 3 ml *in vacuo* using a warm water bath (1×10^{-3} mbar, 35 °C). The product crystallizes in form of large red blocks from a concentrated *n*-pentane solution overnight (these crystals are also suitable for X-ray diffraction). The supernatant was removed with a syringe and discarded. The resulting crystalline product **1** was dried under a dynamic vacuum using a warm water bath for three hours (1×10^{-3} mbar, 45 °C). Yield: 92.0 mg, 0.0947 mmol, 60.7%.

EA for $C_{55}H_{79}AsGaN_4P$ (M.W. = 971.88 g/mol) Calcd. (found) in %: C 67.97 (67.50), H 8.19 (8.69), N 5.76 (5.66). **^{1}H NMR** (C_6D_6, 298.0 K, 499.93 MHz): δ (ppm) 7.16–7.25 (m, 8H; ArC*H*), 7.05 (d, $^3J_{H–H}$ = 7.6 Hz, 4H; ArC*H*), 4.86 (s, 1H; NacNac γ-H), 4.32–4.36 (m, 2H; [SP]Dipp{C*H*(CH_3)$_2$}), 3.95–4.03 (m, 2H; NC*H*$_2$)$_2$), 3.56 (sept, $^3J_{H–H}$ = 6.8 Hz, 2H; [SP]{C*H*(CH_3)$_2$}), 3.27–3.36 (m, 2H; NC*H*$_2$), 2.89 (sept, $^3J_{H–H}$ = 6.8 Hz, 4H, NacnNac{C*H*(CH_3)$_2$}), 1.35–1.42 (m, 18H; NacNacC*H*$_3$ and Dipp{CH(C*H*$_3$)$_2$}), 1.31 (d, *J* = 6.9 Hz, 6H; Dipp{CH(C*H*$_3$)$_2$}), 1.29 (d, *J* = 6.9 Hz, 6H; Dipp{CH(C*H*$_3$)$_2$}), 1.02 (d, $^3J_{H–H}$ = 6.7 Hz, 12H; Dipp{CH(C*H*$_3$)$_2$}), 0.98 (d, $^3J_{H–H}$ = 6.9 Hz, 12H; Dipp{CH(C*H*$_3$)$_2$}). **^{13}C{^{1}H} NMR** (C_6D_6, 298.1 K, 125.71 MHz): δ (ppm) 170.0 (s; Ar*C*), 151.5 (s; Ar*C*), 149.7 (d, $^3J_{C–P}$ = 1.8 Hz; Ar*C*), 144.4 (s; Ar*C*), 143.2 (s; Ar*C*), 140.4 (s; Ar*C*), 128.8 (s; Ar*C*H), 128.5 (s; Ar*C*H), 128.3 (s; Ar*C*H), 125.8 (s; Ar*C*H), 125.4 (s; Ar*C*H), 124.7 (s; Ar*C*H), 123.5 (s; Ar*C*H), 99.1 (s; NacNac*C*H), 57.7 (s; N*C*H$_2$), 29.3 (s; Dipp{*C*H(CH_3)$_2$}), 29.1 (d, $^3J_{C–P}$ = 7.3 Hz; Dipp{*C*H(CH_3)$_2$}), 27.1 (s; Dipp{CH(*C*H$_3$)$_2$}), 26.9 (s; Dipp{CH(*C*H$_3$)$_2$}), 26.1 (d, $J_{C–P}$ = 2.7 Hz; Dipp{CH(*C*H$_3$)$_2$}), 25.2 (d, $J_{C–P}$ = 1.8 Hz; Dipp{CH(*C*H$_3$)$_2$}), 24.5 (s; Dipp{*C*H(CH_3)$_2$}), 23.7 (s; NacNac*C*H$_3$). **^{31}P{^{1}H} NMR** (C_6D_6, 298.0 K, 202.37 MHz): δ (ppm) −72.2 (m; As*P*Ga). **IR** (ATR measurement, 64 scans, cm^{-1}): 3058 (w), 3022 (w), 2960 (s), 2926 (m), 2866 (m), 2819 (m), 1588 (w), 1554 (m),

1526 (s), 1460 (s), 1438 (s), 1383 (s), 1361 (s), 1318 (s), 1255 (m), 1210 (m), 1178 (m), 1146 (m), 1103 (m), 1068 (m), 1056 (m), 1039 (m), 1021 (m), 934 (m), 895 (m), 868 (m), 797 (vs), 775 (m), 755 (s), 708 (m), 632 (m), 605 (m), 571 (m), 552 (m), 507 (m), 483 (m).

Figure S1. NMR and IR spectra of **1** (C_6D_6 solvent signals indicated by asterisks).

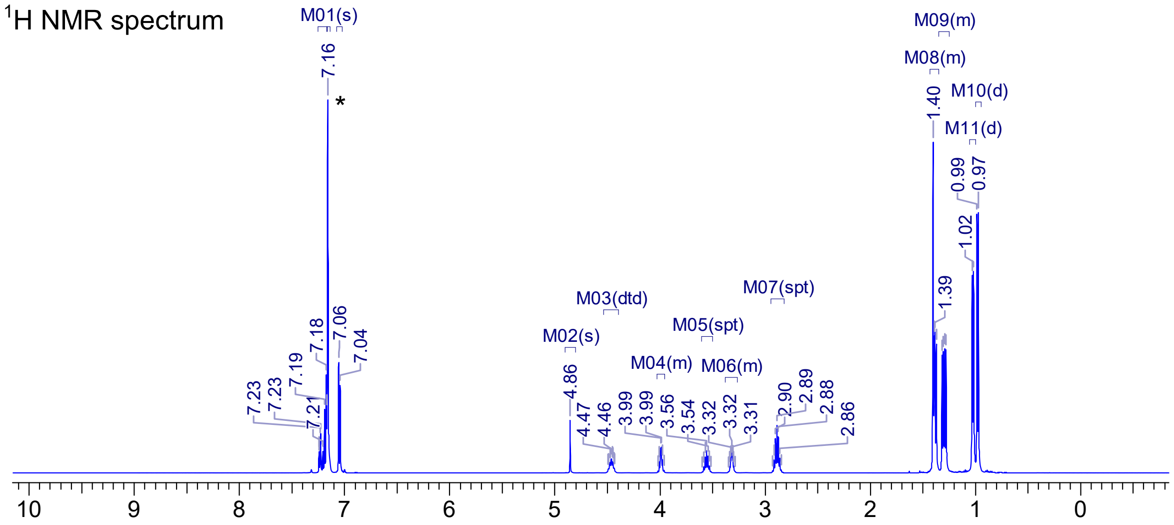

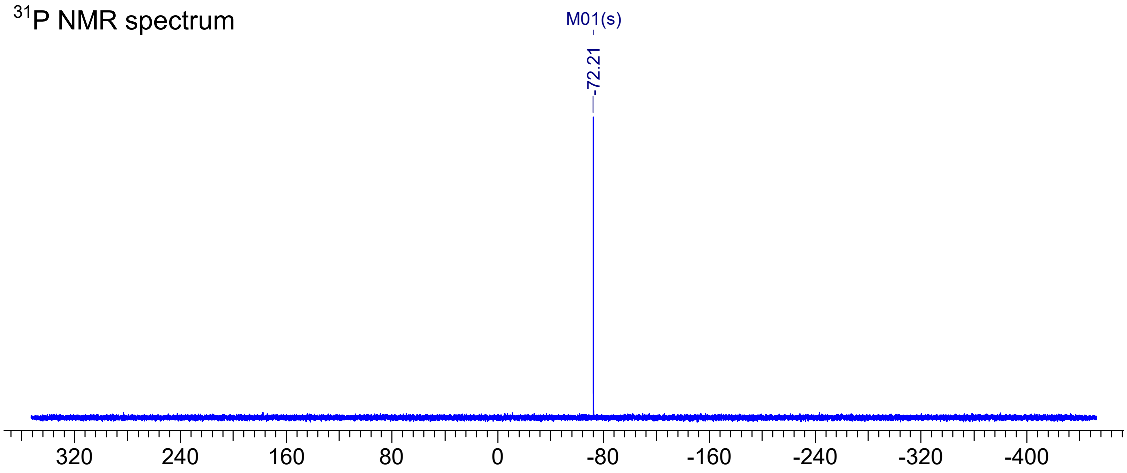

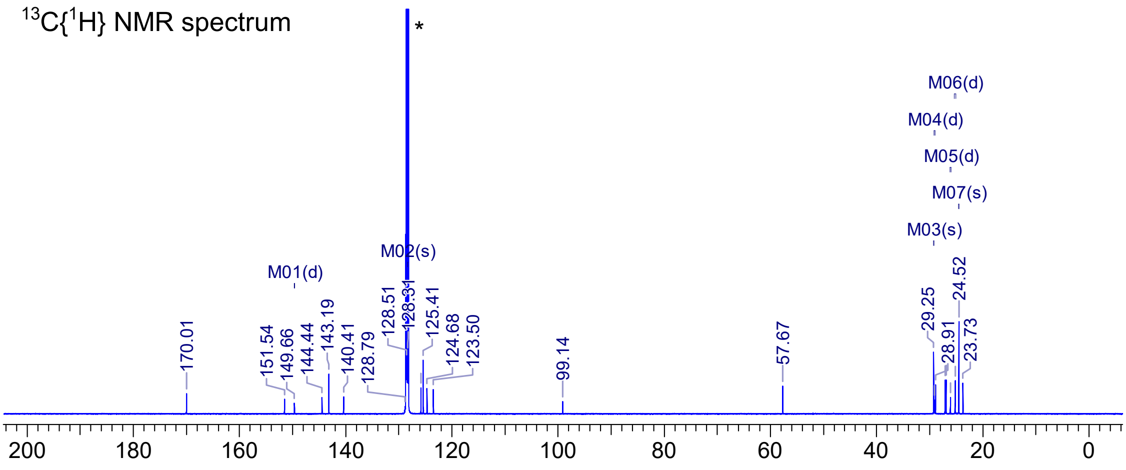
13C{1H} NMR spectrum
*
M06(d)
M04(d)
M05(d)
M07(s)
M03(s)
M02(s)
M01(d)
170.01
151.54
149.66
144.44
143.19
140.41
128.79
128.51
128.31
125.41
124.68
123.50
99.14
57.67
29.25
28.91
24.52
23.73
200
180
160
140
120
100
80
60
40
20
0

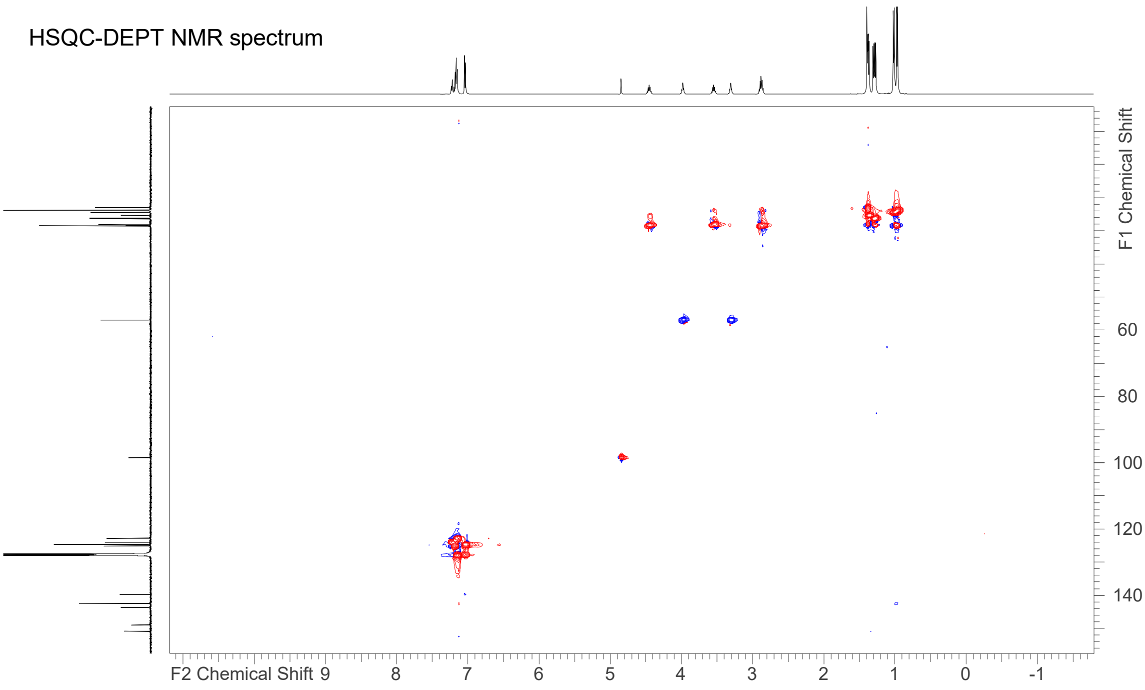
HSQC-DEPT NMR spectrum
F1 Chemical Shift
60
80
100
120
140
F2 Chemical Shift 9
8
7
6
5
4
3
2
1
0
-1

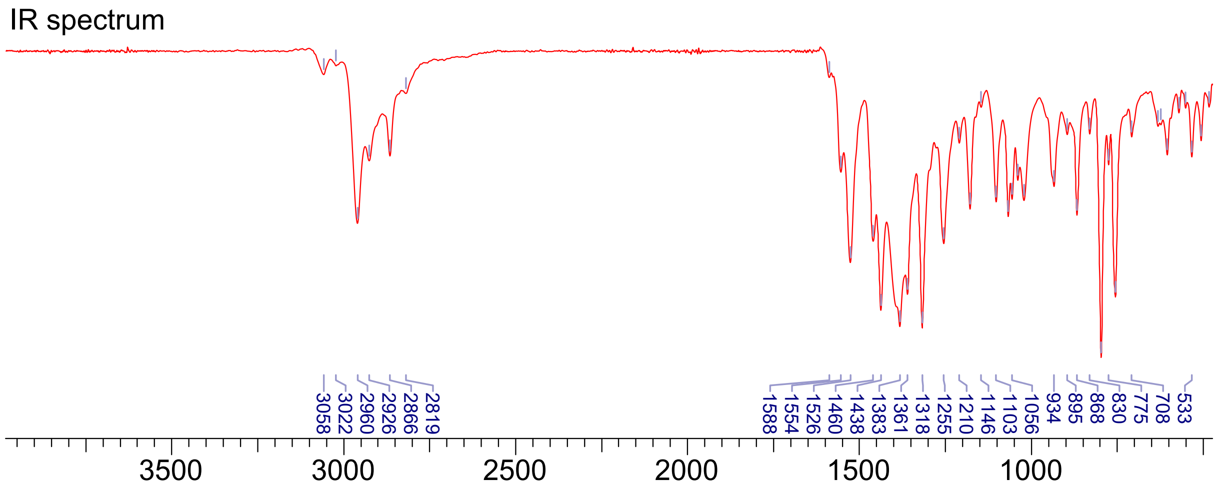

2.2 Synthesis of 2

1 (35 mg, 0.036 mmol) was dissolved in C_6H_6 (0.7 ml) in a J. Young NMR tube. *Tert*-butyl azide (1.05 eq., 3.70 mg, 0.0370 mmol) was added to the NMR tube causing the solution to quickly turn orange. All volatile components were removed *in vacuo* (1×10^{-3} mbar, 25 °C) and the orange solid extracted with *n*-hexane. The solution was filtered into a small vial and kept at −30 °C overnight resulting in the formation of orange crystals of **2**. The supernatant was removed, discarded and the product dried under a dynamic vacuum for three hours (1×10^{-3} mbar, 25 °C). Yield: 23.2 mg, 0.0217 mmol, 61.0%. Crystals suitable for X-ray diffraction were grown from a mixture of *n*-hexane/benzene (1:1) at room temperature.

Due to the (partly) reversible adduct formation with $^{t}BuN_3$ at high temperatures, it was not possible to obtain an accurate elemental analysis of **2**.

EA for $C_{59}H_{88}AsGaN_7P$ (M.W. = 1071.01 g/mol) Calcd. (found) in %: C 66.17 (67.85), H 8.28 (8.00), N 7.00 (7.20). **^{1}H NMR** (C_6D_6, 298.0 K, 499.98 MHz): δ (ppm) 7.16–7.20 (m, 2H; ArC*H*), 7.12–7.15 (m, 2H; ArC*H*), 7.02–7.08 (m, 4H; ArC*H*), 6.86–6.97 (m, 4H; ArC*H*), 4.88 (s, 1H; NacNac γ-H), 4.50–4.65 (m, 2H; Dipp{C*H*(CH_3)$_2$}), 4.15–4.30 (m, 2H; NCH_2), 3.30–3.50 (m, 4H; NCH_2 and Dipp{C*H*(CH_3)$_2$}), 3.15–3.38 (m, 2H; Dipp{C*H*(CH_3)$_2$}), 2.80–2.95 (m, 2H; Dipp{C*H*(CH_3)$_2$}), 1.60 (s, 9H; $^{t}BuCH_3$), 1.29 (s, 6H; NacNacCH_3), 1.25 (d, $^3J_{H\text{–}H}$ = 6.9 Hz, 6H; Dipp{CH(CH_3)$_2$}), 1.18–1.23 (m, 12H; Dipp{CH(CH_3)$_2$}), 1.13–1.17 (m, 6H; Dipp{CH(CH_3)$_2$}), 1.02 (br. d, $^3J_{H\text{–}H}$ = 6.7 Hz, 12H; Dipp{CH(CH_3)$_2$}), 0.74 (br. s, 6H; Dipp{CH(CH_3)$_2$}), 0.60 (br. d, $^3J_{H\text{–}H}$ = 6.3 Hz, 6H;

Dipp{CH(CH_3)$_2$}). **^{13}C{^{1}H} NMR** (C_6D_6, 298.0 K, 125.72 MHz): δ (ppm) 171.1 (s; Ar*C*), 151.3 (s; Ar*C*), 148.0 (br. s; Ar*C*), 145.3 (br. s; Ar*C*), 143.7 (br. S; Ar*C*), 142.6 (br. s; Ar*C*),142.3 (br. s; Ar*C*), 128.7 (s; Ar*C*H), 128.0 (s; Ar*C*H), 126.5 (s; Ar*C*H), 125.7 (s; Ar*C*H), 125.5 (s; Ar*C*H), 125.0 (s; Ar*C*H), 123.7 (s; Ar*C*H), 101.4 (br. s; NacNac*C*H), 59.5 (br. s; N*C*H_2), 57.4 (br. s; N*C*H_2), 32.4 (br. s; ^{t}Bu*C*H_3), 32.2 (s; Dipp{*C*H(CH$_3$)$_2$}), 29.5 (br. s; Dipp{*C*H(CH$_3$)$_2$}), 28.9 (br. s; Dipp{*C*H(CH$_3$)$_2$}), 28.8 (s; Dipp{*C*H(CH$_3$)$_2$}), 28.7 (br. d, $J_{C–P}$ = 5.5 Hz; Dipp{*C*H(CH$_3$)$_2$}), 27.2 (br. d, $J_{C–P}$ = 5.9 Hz; Dipp{CH(*C*H$_3$)$_2$}), 26.6 (br. s; Dipp{CH(*C*H$_3$)$_2$}), 25.4 (br. s; Dipp{CH(*C*H$_3$)$_2$}), 25.1 (br. d, $J_{C–P}$ = 1.0 Hz; Dipp{CH(*C*H$_3$)$_2$}), 24.8 (br. s; Dipp{CH(*C*H$_3$)$_2$}), 24.7 (br. s; Dipp{CH(*C*H$_3$)$_2$}), 23.4 (br. s, NacNac*C*H_3). **^{31}P{^{1}H} NMR** (C_6D_6, 298.0 K, 202.38 MHz): δ (ppm) −31.6 (s; As*P*Ga). **IR** (ATR measurement, 64 scans, cm^{-1}): 3042 (w), 2941 (s), 2909 (m), 2849 (m), 2805 (m), 1578 (m), 1527 (m), 1452 (s), 1428 (s), 1374 (s), 1354 (s), 1308 (m), 1274 (m), 1241 (m), 1197 (m); 1168 (m), 1098 (m), 1062 (s), 1042 (vs), 1016 (s), 979 (s), 952 (m), 932 (m), 891 (m), 861 (m), 825 (w), 813 (m), 793 (vs), 752 (s), 723 (w), 701 (m), 635 (w), 622 (w), 603 (w), 593 (m), 580 (w), 556 (m), 535 (s), 515 (m), 502 (m), 492 (m), 479 (m).

Figure S2. NMR and IR spectra of **2**. The crystals were dried *in vacuo* for 3 h (1×10^{-3} mbar, 25 °C), however, the sample still contains slight traces of *n*-pentane (^{1}H NMR: 0.87 ppm, 1.23 ppm; ^{13}C NMR: 14.3 ppm, 22.7 ppm, 34.5 ppm), indicated with asterisks. The solvent signals (C_6D_6, ^{1}H NMR: 7.16 ppm; ^{13}C NMR: 128.4 ppm) are also indicated with asterisks.

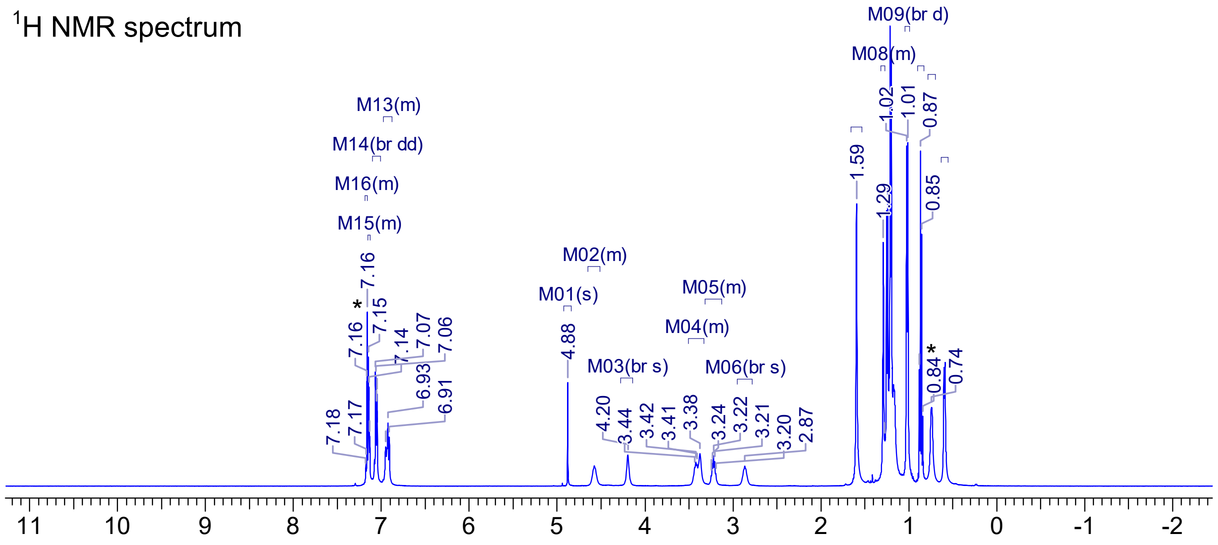

$^{31}P\{^{1}H\}$ NMR spectrum

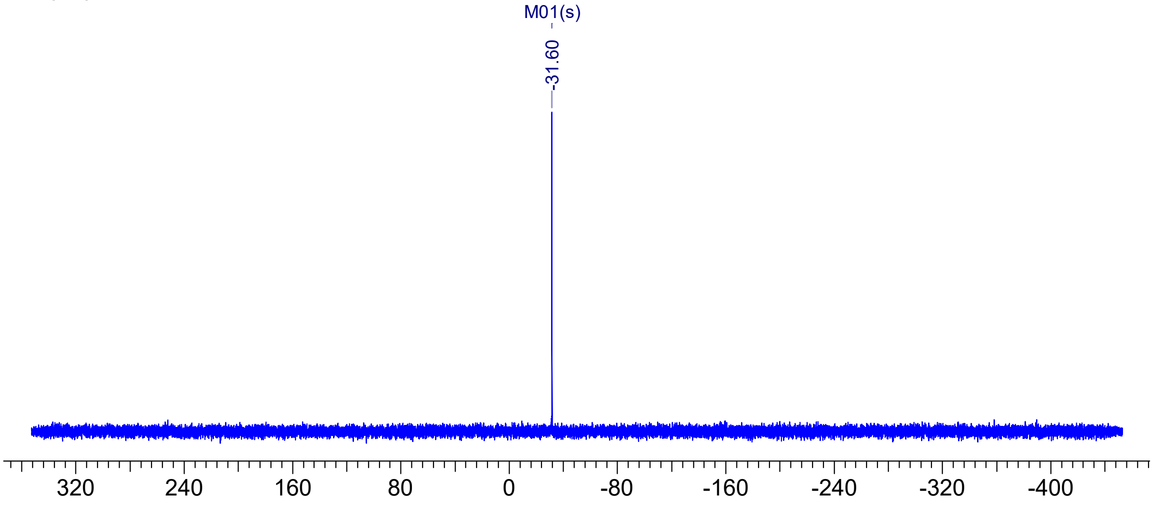

$^{13}C\{^{1}H\}$ NMR spectrum

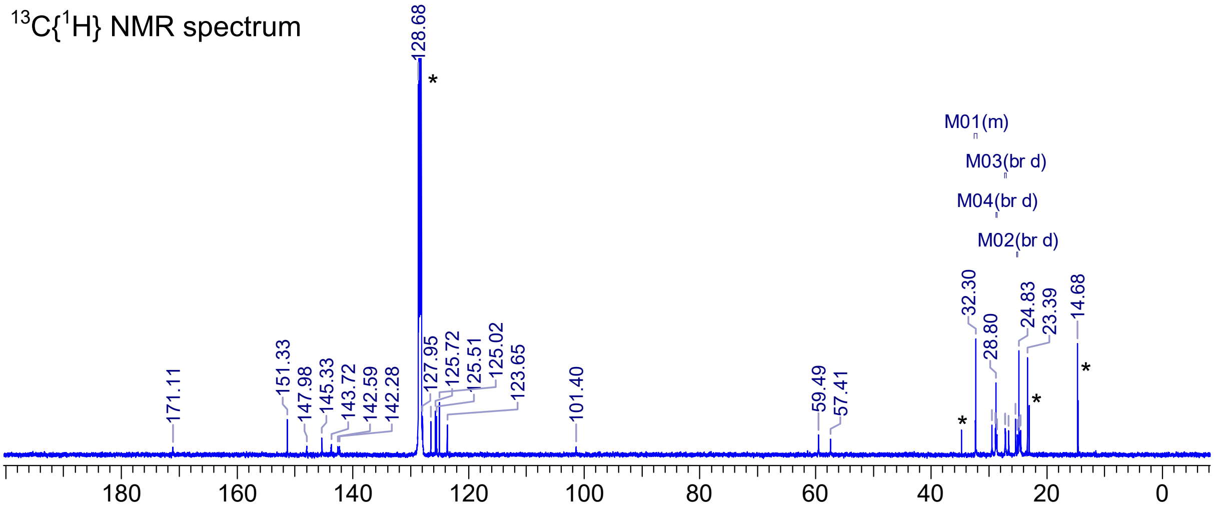

IR spectrum

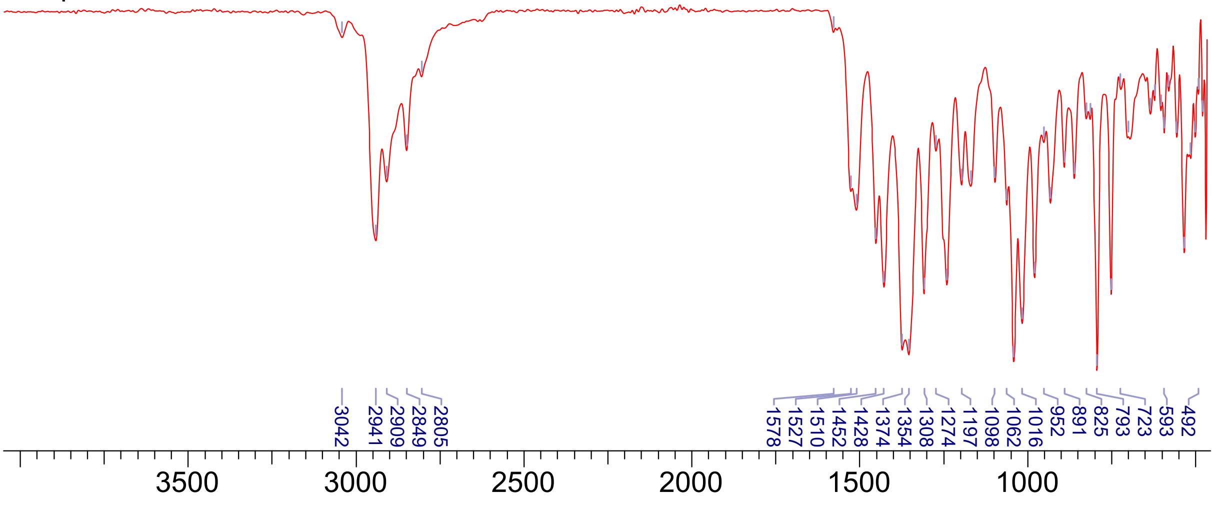

2.3 Synthesis of 3

1 (34.0 mg, 0.0350 mmol) was dissolved in C_6H_6 (0.7 ml) in a J. Young NMR tube. *N,N'*-Dicyclohexylcarbodiimide (1.05 eq., 7.57 mg, 0.0370 mmol) was added to the NMR tube, after which the solution quickly turned yellow. The solution was filtered into a small vial affording colourless crystals of **3** within minutes. The filtration needs to be carried out quickly, otherwise the product can precipitate in the filter. The supernatant was removed, discarded and the resulting colourless crystalline solid was washed with small amounts of cold *n*-hexane (3 × 0.5 ml, −30 °C). The resulting crystalline solid was dried *in vacuo* for three hours (1×10^{-3} mbar, 25 °C). Yield: 9.70 mg, 0.008 mmol, 23.5%.

EA for $C_{68}H_{101}AsGaN_6P$ (M.W. = 1178.21 g/mol) Calcd. (found) in %: C 69.32 (70.27), H 8.64 (8.18), N 5.92 (6.79). **^{1}H NMR** (THF-d_8, 298.0 K, 600.42 MHz): δ (ppm) 7.08–7.13 (m, 6H; ArC*H*), 7.03–7.07 (m, 4H; ArC*H*), 7.00–7.03 (br. s, 2H; ArC*H*), 6.93–6.98 (m, 4H; ArC*H*), 5.32 (s, 1H; Nacnac γ-H), 3.90–3.98 (m, 2H; NCH_2), 3.70–3.78 (m, 2H; Dipp{C*H*(CH_3)$_2$}), 3.40–3.47 (m, 1H; CyC*H*), 3.25–3.35 (m, 6H; NCH_2 and Dipp{C*H*(CH_3)$_2$}), 3.18–3.25 (m, 1H; CyC*H*), 2.89 (sept, $^3J_{H–H}$ = 6.9 Hz, 2H; Dipp{C*H*(CH_3)$_2$}), 2.42 (br. d, $^3J_{H–H}$ = 10.9 Hz, 2H; CyCH_2), 1.88 (br. d, $^3J_{H–H}$ = 12.9 Hz, 2H; CyCH_2), 1.65 (br. s, 8H; CyCH_2 and NacNacCH_3), 1.53–1.62 (m, 2H; CyCH_2), 1.25–1.38 (m, 10H; CyCH_2), 1.09 (br. d, $^3J_{H–H}$ = 6.8 Hz, $^3J_{H–H}$ = 2.3 Hz, 12H; Dipp{CH(CH_3)$_2$}), 1.05 (br. s, 3H; Dipp{CH(CH_3)$_2$}), 1.04 (br. d, $^3J_{H–H}$ = 3.8 Hz, 6H; Dipp{CH(CH_3)$_2$}), 1.02 (br. d, $^3J_{H–H}$ = 4.5 Hz, 6H; Dipp{CH(CH_3)$_2$}), 1.00 (br. s, 6H; Dipp{CH(CH_3)$_2$}), 0.99 (br. s, 3H; Dipp{CH(CH_3)$_2$}), 0.93–0.98 (m, 2H; CyCH_2), 0.85 (br. d, $^3J_{H–H}$ = 6.7 Hz, 6H; Dipp{CH(CH_3)$_2$}), 0.68 (br. d, $^3J_{H–H}$ = 6.7 Hz, 6H; Dipp{CH(CH_3)$_2$}). **^{13}C{^{1}H} NMR** (THF-d_8, 298.0 K, 150.97 MHz): δ (ppm) 171.3 (s; Ar*C*), 164.0 (d, $^1J_{C–P}$ = 68.1 Hz; NCN), 150.8 (s; Ar*C*), 148.6 (s; Ar*C*), 145.7 (s; Ar*C*), 144.0 (s; Ar*C*), 143.3 (s; Ar*C*), 142.5 (s; Ar*C*), 128.3 (s; Ar*C*H), 126.6 (s; Ar*C*H), 125.6 (s; Ar*C*H), 125.3 (s; Ar*C*H), 125.2 (s; Ar*C*H), 124.3 (s; Ar*C*H), 99.2 (s; NacNac*C*H), 66.5 (d, $J_{C–P}$ = 19.1 Hz; Cy*C*H), 60.2 (s, N*C*H_2), 57.7 (s; Cy*C*H), 36.6 (s; Cy*C*H_2), 35.4 (s, Cy*C*H_2), 29.7 (d, $J_{C–P}$ = 12.0 Hz, Cy*C*H_2), 29.0 (d, $J_{C–P}$ = 4.9 Hz; Cy*C*H_2), 28.8 (s; Dipp{*C*H(CH_3)$_2$}), 28.2 (s; Dipp{*C*H(CH_3)$_2$}), 27.7 (s; Dipp{*C*H(CH_3)$_2$}), 27.4 (d, $J_{C–P}$ = 8.7 Hz; Cy*C*H_2), 27.0 (s; Dipp{*C*H(CH_3)$_2$}), 26.0 (s; Dipp{CH(*C*H_3)$_2$}), 25.8 (s; Dipp{CH(*C*H_3)$_2$}), 25.0 (s; Dipp{CH(*C*H_3)$_2$}), 24.9 (s; Dipp{CH(*C*H_3)$_2$}), (d, $J_{C–P}$ = 6.5 Hz; NacNac*C*H_3). **^{31}P{^{1}H} NMR** (THF-d_8, 298.0 K, 243.05 MHz): δ (ppm) −27.6 (s;

As*P*Ga). **IR** (ATR measurement, 64 scans, cm^{-1}): 3044 (w), 2951 (m), 2909 (m), 2848 (m), 2833 (m), 2819 (s), 2776 (s), 1558 (m), 1548 (m), 1504 (m), 1451 (m), 1430 (s), 1378 (s), 1364 (m), 1351 (m), 1337 (w), 1308 (m), 1289 (s), 1277 (w), 1249 (m), 1242 (m), 1208 (m), 1191 (m), 1141 (w), 1101 (m), 1061 (m), 1052 (m), 1032 (m), 1018 (m), 978 (w), 931 (m), 918 (w), 894 (w), 883 (m), 863 (m), 855 (w), 823 (m), 821 (m), 792 (s), 751 (s), 726 (w), 718 (w), 712 (w), 706 (w), 698 (w), 675 (vs), 645 (w), 633 (w), 628 (w), 623 (w), 619 (w), 613 (w), 600 (w), 582 (w), 588 (w), 555 (m), 530 (m), 508 (m), 493 (m), 482 (m).

Figure S3. NMR and IR spectra of **3** (solvent signals indicated with asterisks).

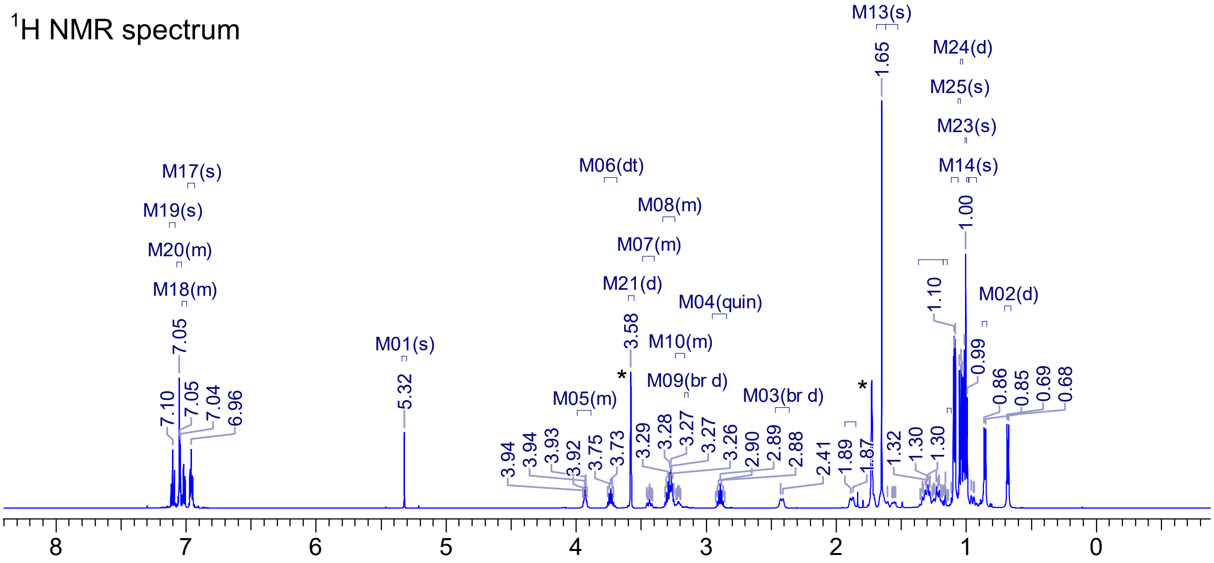

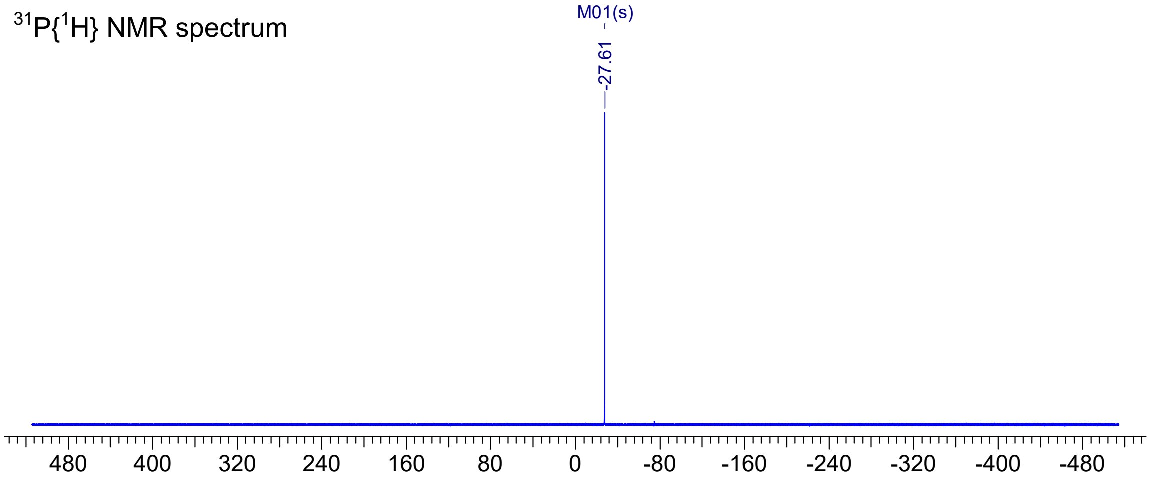

$^{13}C\{^1H\}$ NMR spectrum

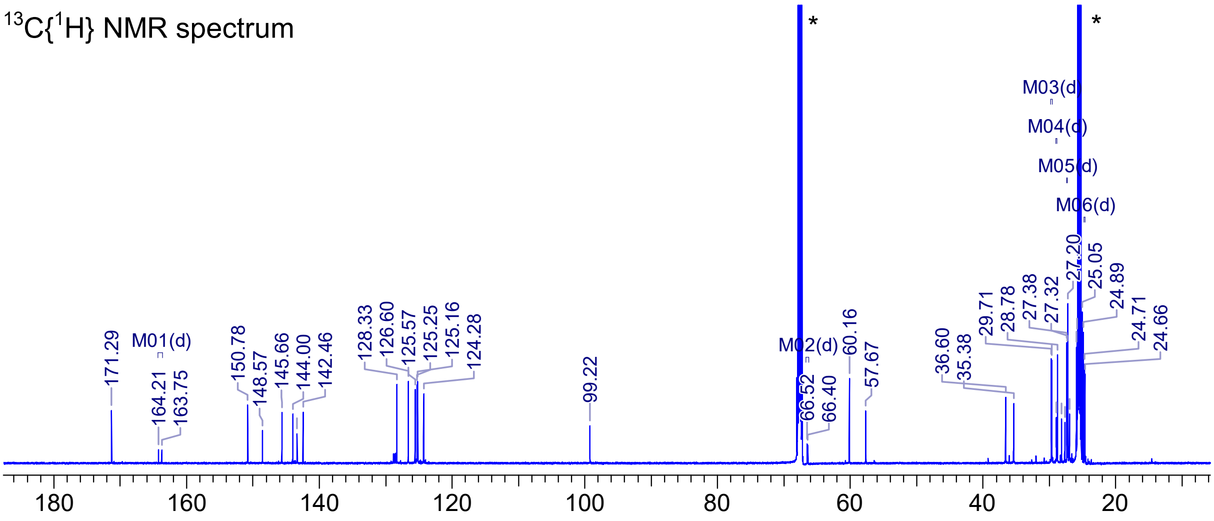

IR spectrum

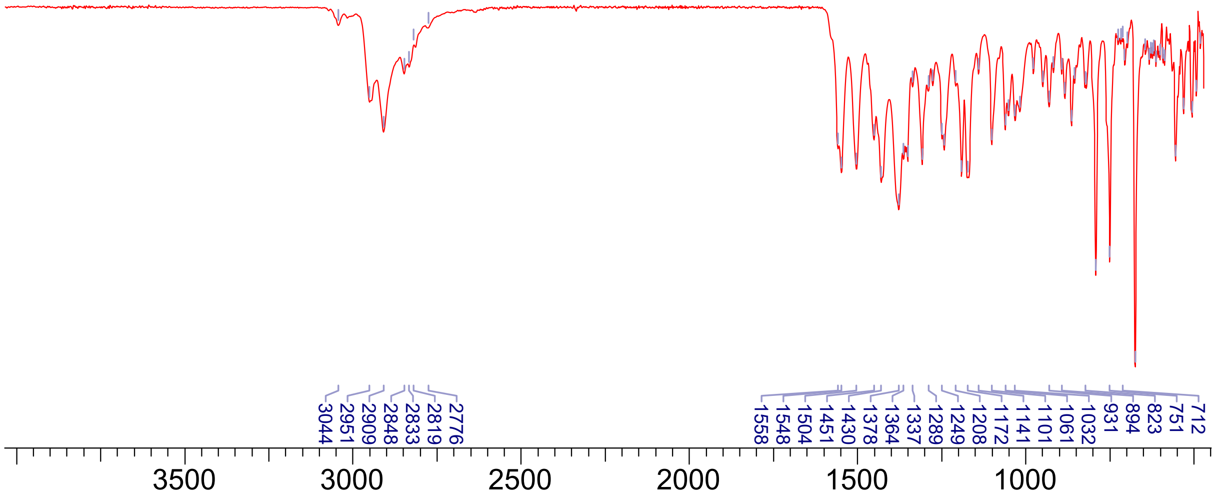

2.4 Synthesis of 4

1 (52.0 mg, 0.0540 mmol) was dissolved in C_6H_6 (0.7 ml) in a J. Young NMR tube. 2,4,6-trimethylphenyl isocyanate (1.06 eq., 9.10 mg, 0.0560 mmol) was added to the NMR tube, and the resulting reaction mixture turned yellow. After the addition, all volatile components were removed *in vacuo* (1×10^{-3} mbar, 25 °C) and the yellow solid was extracted with *n*-hexane. The solution was filtered into a small vial. Storage of the solution at −30 °C overnight afforded yellow crystals of **4**. The supernatant was removed and discarded, and the solid washed with small amounts of cold *n*-hexane (3

× 0.3 ml, −30 °C). The resulting yellow product was dried under a dynamic vacuum for three hours (1×10^{-3} mbar, 25 °C). Yield: 33.7 mg, 0.030 mmol, 55.5%. Crystals suitable for X-ray diffraction were grown from a mixture of *n*-hexane/benzene (1:1) at room temperature.

EA for $C_{65}H_{90}AsGaN_5OP$ (M.W. = 1133.08 g/mol) Calcd. (found) in %: C 68.90 (69.55), H 8.01 (7.91), N 6.18 (6.29). **^{1}H NMR** (C_6D_6, 298.0 K, 600.42 MHz): δ (ppm) 7.20–7.35 (m, 6H; ArC*H*), 7.02–7.15 (m, 3H; ArC*H*), 6.95–7.01 (br. s, 2H; Mes ArC*H*), 6.85–6.90 (br. s, 1H; Mes ArC*H*), 6.59 (s, 1H; Mes ArC*H*), 4.66 (s, 1H; NacNac γ-H), 4.08 (br. s, 1H; Dipp{C*H*(CH_3)$_2$}), 3.94 (br. s, 1H; NCH_2), 3.85 (br. s, 1H; Dipp{C*H*(CH_3)$_2$}), 3.74 (br. s, 2H; Dipp{C*H*(CH_3)$_2$}), 3.65 (br. s, 1H; NCH_2), 3.48 (br. s, 2H; NCH_2 and Dipp{C*H*(CH_3)$_2$}), 3.24 (br. s, 1H; NCH_2), 2.87–3.05 (m, 2H; Dipp{C*H*(CH_3)$_2$}), 2.79 (br. s, 1H; Dipp{C*H*(CH_3)$_2$}), 2.14 (s, 3H; MesCH_3); 1.88 (br. s, 3H; MesCH_3), 1.15–1.53 (m, 42H; MesCH_3 and/or NacNacCH_3 and/or Dipp{CH(CH_3)$_2$}), 0.90–1.05 (m, 9H; MesCH_3 and/or NacNacCH_3 and/or Dipp{CH(CH_3)$_2$}), 0.73 (br. s, 3H; MesCH_3 and/or NacNacCH_3 and/or Dipp{CH(CH_3)$_2$}), 0.50 (br. s, 3H; MesCH_3 and/or NacNacCH_3 and/or Dipp{CH(CH_3)$_2$}). **^{13}C{^{1}H} NMR** (C_6D_6, 298.0 K, 150.97 MHz): δ (ppm) 171.5 (br. s; Ar*C*), 171.1 (s; Ar*C*), 171.0 (s; Ar*C*), 170.9 (s; Ar*C*), 150.7 (br. s; Ar*C*), 150.0 (br. s; Ar*C*), 148.4 (br. s; Ar*C*), 147.0 (d, $^3J_{C\text{–}P}$ = 5.5 Hz; Ar*C*), 146.7 (br. s; Ar*C*), 145.7 (br. s; Ar*C*), 144.6 (br. s; Ar*C*), 143.9 (br. s; Ar*C*), 142.8 (br. s; Ar*C*), 150.7 (br. s; Ar*C*), 139.9 (br. d, $^1J_{C\text{–}P}$ = 46.3 Hz; MesN*C*O), 129.2 (s; Ar*C*H), 128.9 (s; Ar*C*H), 128.7 (s; Ar*C*H), 127.9 (br. s; Ar*C*H), 127.3 (br. s; Ar*C*H), 126.0 (br. s; Ar*C*H), 125.4 (br. s; Ar*C*H), 125.1 (br. s; Ar*C*H), 125.0 (br. s; Ar*C*H), 124.3 (br. s; Ar*C*H), 123.9 (br. s; Ar*C*H), 97.5 (s; NacNac*C*H), 59.1 (br. d, $^3J_{C\text{–}P}$ = 27.8 Hz; N*C*H_2), 29.9 (br. s; Dipp{*C*H(CH_3)$_2$}), 29.4 (br. d, $J_{C\text{–}P}$ = 13.1 Hz; Dipp{*C*H(CH_3)$_2$}), 28.8 (br. s; Dipp{*C*H(CH_3)$_2$}), 26.8 (br. s; Dipp{CH(*C*H_3)$_2$}), 26.5 (br. s; Dipp{CH(*C*H_3)$_2$}), 26.0 (br. s; Dipp{CH(*C*H_3)$_2$}), 25.7 (br. s; Dipp{CH(*C*H_3)$_2$}), 25.3 (br. s; Dipp{CH(*C*H_3)$_2$}), 25.2 (br. s; Dipp{CH(*C*H_3)$_2$}), 25.1 (br. s; Dipp{CH(*C*H_3)$_2$}), 24.8 (br. s; Dipp{CH(*C*H_3)$_2$}), 24.1 (br. s; Dipp{CH(*C*H_3)$_2$}), 23.9 (br. d, $J_{C\text{–}P}$ = 18.0 Hz; Dipp{CH(*C*H_3)$_2$}), 21.3 (s; NacNac*C*H_3), 18.8 (s, MesCH$_3$). **^{31}P{^{1}H} NMR** (C_6D_6, 298.0 K, 243.05 MHz): δ (ppm) −34.5 (s; As*P*Ga). **IR** (ATR measurement, 64 scans, cm^{-1}): 3045 (w), 3002 (vw), 2942 (s), 2909 (m), 2891 (m), 2849 (m), 2815 (s), 1571 (s), 1554 (w), 1531 (m), 1515 (s), 1455 (s), 1430 (s), 1371 (vs), 1351 (s), 1308 (vs), 1247 (s), 1202 (m), 1175 (m), 1142 (m), 1142 (m), 1097 (s), 1091 (s), 1062 (s), 1048 (m), 1018 (m), 961 (w), 938 (w), 929 (w), 895 (m), 868 (m), 855 (m), 831 (m), 793 (vs), 776 (m), 756 (m), 748 (m), 726 (s), 713 (m), 692 (m), 680

(m), 663 (w), 643 (w), 628 (w), 619 (w), 609 (w), 602 (w), 589 (m), 569 (m), 549 (m), 543 (m), 539 (m), 512 (m), 487 (m), 476 (m).

Figure S4. NMR and IR spectra of **4** (solvent signals indicated with asterisks).

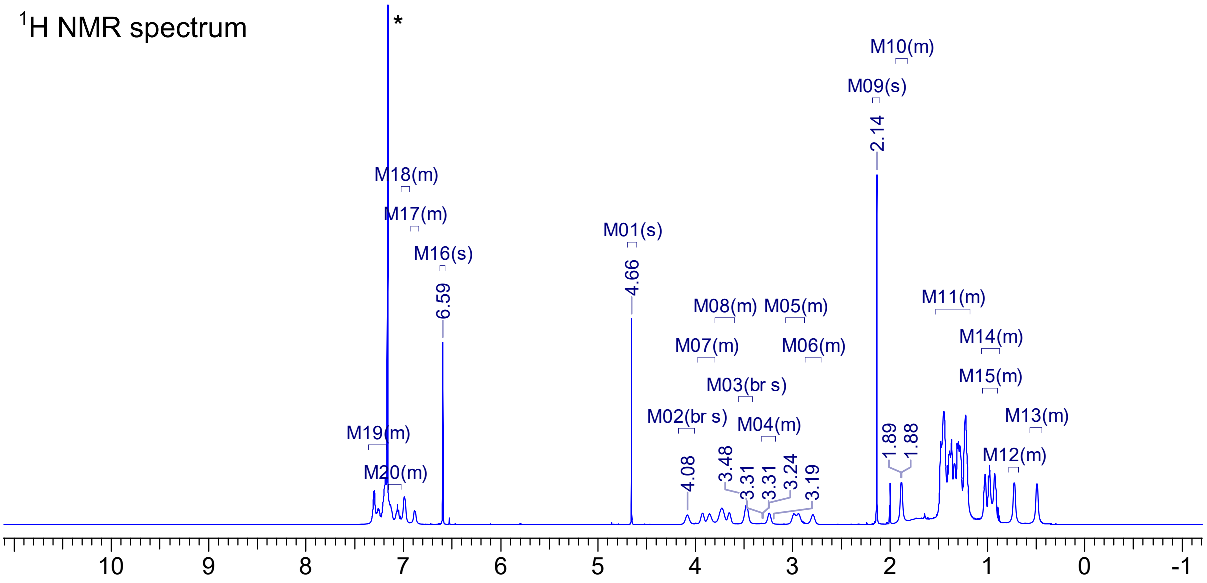

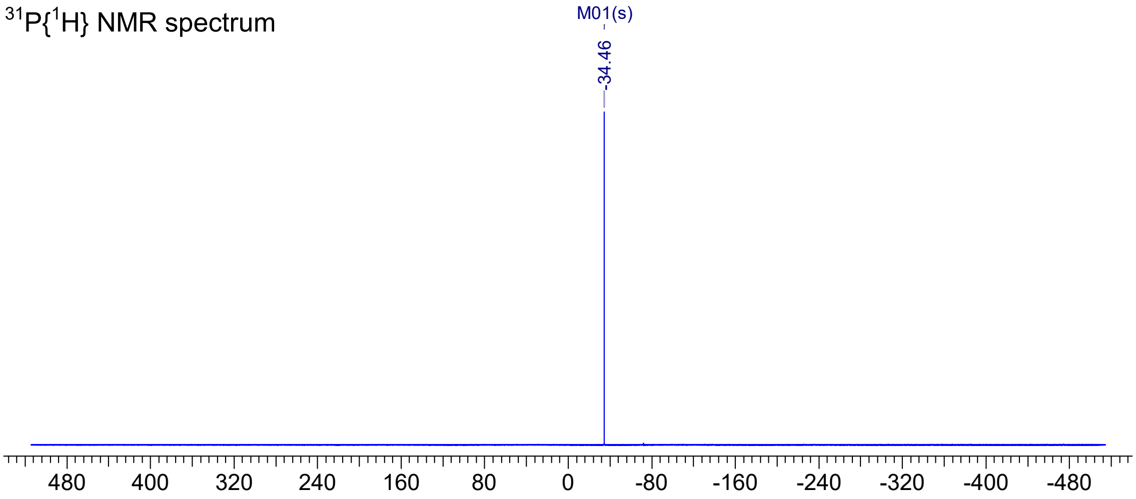

$^{13}C\{^{1}H\}$ NMR spectrum

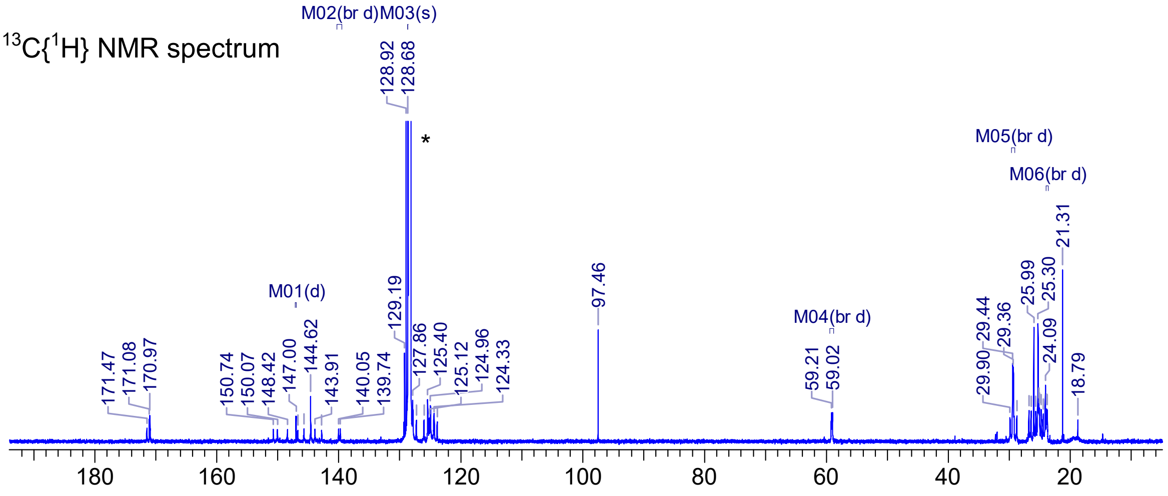

IR spectrum

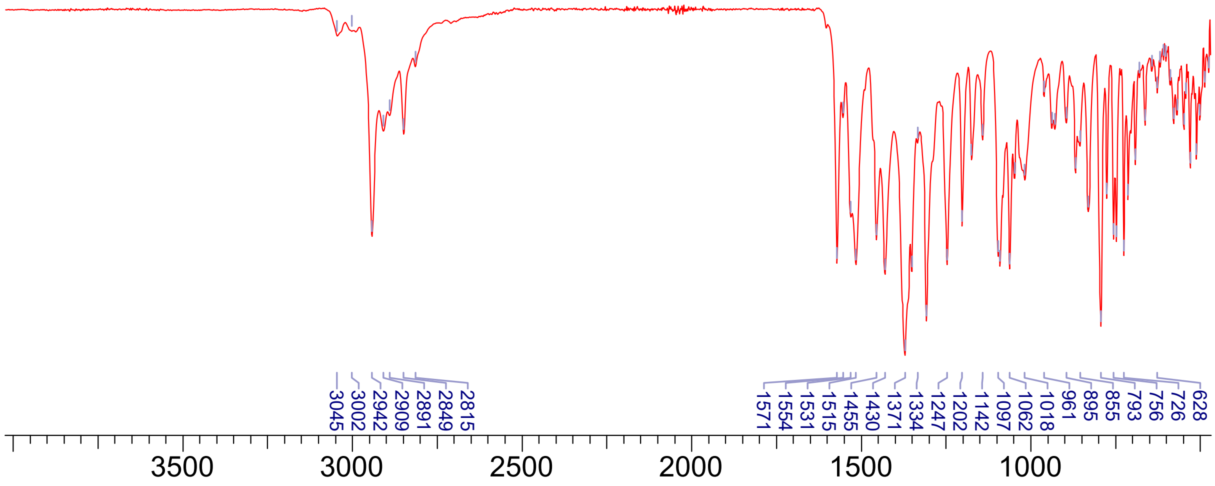

2.5 Synthesis of 5

1 (58.0 mg, 0.060 mmol) was dissolved in C_6H_6 (0.7 ml) in a J. Young NMR tube. Benzaldehyde (1.35 eq., 8.60 mg, 0.0810 mmol) was added to the NMR tube causing the solution to quickly turn yellow. After addition, all volatile components were removed *in vacuo* ($1×10^{-3}$ mbar, 25 °C) and the yellow solid extracted with *n*-hexane. The solution was filtered into a small vial. After storage at −30 °C overnight, yellow crystals of **5** were obtained. The supernatant was removed and discarded, and the product was dried *in vacuo* for three hours ($1×10^{-3}$ mbar, 25 °C). Yield: 22.5 mg, 0.0210 mmol, 35.0%. Crystals suitable for X-ray diffraction were grown from a mixture of *n*-hexane/benzene (1:1) at room temperature.

E.A. for $C_{62}H_{85}AsGaN_4OP$ (M.W.= 1078.00 g/mol) Calcd. (found) in %: C 69.08 (69.27), H 7.95 (7.63), N 5.20 (5.28). **^{1}H NMR** (C_6D_6, 298.0 K, 499.93 MHz): δ (ppm) 7.32–7.37 (m, 2H; ArC*H*), 7.20–7.28 (m, 3H; ArC*H*), 7.17–7.19 (m, 2H; ArC*H*), 7.08–7.14 (m, 5H; ArC*H*), 6.70–6.75 (m, 1H; Ph ArC*H*), 6.61 (t, $^3J_{H–H}$ = 7.6 Hz, 2H; Ph ArC*H*), 5.96 (br. d, ^3J = 7.1 Hz, 2H; Ph ArC*H*); 5.59 (d, $^3J_{P–H}$ = 34 Hz, 1H; PhC*H*O); 4.81 (s, 1H; Nacnac γ-H), 4.40 (dq, $^3J_{H–H}$ = 12.8 Hz, *J* = 6.6 Hz, 1H; Dipp{C*H*(CH$_3$)$_2$}), 4.23 (td, $^3J_{H–H}$ = 9.7 Hz, *J* = 5.7 Hz, 1H; NC*H*$_2$), 3.91 (sept, $^3J_{H–H}$ = 6.9, 1H; Dipp{C*H*(CH$_3$)$_2$}), 3.57 (sept, $^3J_{H–H}$ = 6.7, 1H; Dipp{C*H*(CH$_3$)$_2$}), 3.30–3.50 (m, 5H; NC*H*$_2$ and Dipp{C*H*(CH$_3$)$_2$}), 3.20 (sept, $^3J_{H–H}$ = 6.8, 1H; Dipp{C*H*(CH$_3$)$_2$}), 3.09–3.17 (m, 1H; NC*H*$_2$); 2.89 (sept, $^3J_{H–H}$ = 6.7, 1H; Dipp{C*H*(CH$_3$)$_2$}), 1.94 (d, $^3J_{H–H}$ = 6.7, 3H; Dipp{CH(C*H*$_3$)$_2$}), 1.59 (s, 3H; Dipp{CH(C*H*$_3$)$_2$}), 1.52 (s, 3H; Dipp{CH(C*H*$_3$)$_2$}), 1.45–1.49 (m, 6H; Dipp{CH(C*H*$_3$)$_2$}), 1.32–1.40 (m, 12H; Dipp{CH(C*H*$_3$)$_2$}), 1.20–1.28 (m, 12H; NacNacC*H*$_3$ and Dipp{CH(C*H*$_3$)$_2$}), 1.19 (d, $^3J_{H–H}$ = 6.9, 3H; Dipp{CH(C*H*$_3$)$_2$}), 1.10–1.16 (m, 6H; Dipp{CH(C*H*$_3$)$_2$}), 0.93 (d, $^3J_{H–H}$ = 6.7, 3H; Dipp{CH(C*H*$_3$)$_2$}), 0.39 (d, $^3J_{H–H}$ = 6.7, 3H; Dipp{CH(C*H*$_3$)$_2$}). **^{13}C{^{1}H} NMR** (C_6D_6, 298.0 K, 125.71 MHz): δ (ppm) 170.7 (s; Ar*C*), 169.4 (s; Ar*C*), 150.7 (s; Ar*C*), 150.2–150.4 (m; Ar*C*), 149.2 (s; Ar*C*), 147.7 (s; Ar*C*), 146.2 (s; Ar*C*), 145.2 (s; Ar*C*), 145.1 (s; Ar*C*), 145.0 (s; Ar*C*), 144.2 (s; Ar*C*), 143.3 (s; Ar*C*), 141.2 (s; Ar*C*), 140.8 (s; Ar*C*), 128.8 (s; Ar*C*H), 128.7 (s; Ar*C*H), 128.3 (s; Ar*C*H), 127.2 (s; Ar*C*H), 126.4 (s; Ar*C*H), 125.7 (s; Ar*C*H), 125.4 (d, $^3J_{C–P}$ = 5.5 Hz; Ar*C*H), 125.1 (s; Ar*C*H), 124.6 (d, $J_{C–P}$ = 30.0 Hz, Ar*C*H), 124.7 (d, $J_{C–P}$ = 13.6 Hz; Ar*C*H) 124.0 (s; Ar*C*H), 123.8 (s; Ar*C*H), 97.9 (s; NacNac*C*H$_3$), 79.3 (d, $^1J_{C–P}$ = 27.3 Hz; Ph*C*HO), 58.7 (d, $^3J_{C–P}$ = 42.7 Hz; N*C*H$_2$), 30.0 (d, $J_{C–P}$ = 14.5 Hz; Dipp{*C*H(CH$_3$)$_2$}), 29.7 (br. s; Dipp{*C*H(CH$_3$)$_2$}), 29.5 (s; Dipp{*C*H(CH$_3$)$_2$}), 29.4 (s;

Dipp{$CH(CH_3)_2$}), 29.3 (s; Dipp{$CH(CH_3)_2$}), 29.0 (s; Dipp{$CH(CH_3)_2$}), 28.2 (s; Dipp{$CH(CH_3)_2$}), 27.7 (s; Dipp{$CH(CH_3)_2$}), 27.4 (s; Dipp{$CH(CH_3)_2$}), 26.9 (s; Dipp{$CH(CH_3)_2$}), 26.5 (s; Dipp{$CH(CH_3)_2$}), 26.0 (s; Dipp{$CH(CH_3)_2$}), 25.7 (d, J_{C-P} = 14.5 Hz; Dipp{$CH(CH_3)_2$}), 25.5 (s; Dipp{$CH(CH_3)_2$}), 25.4 (s; Dipp{$CH(CH_3)_2$}), 25.2 (s; Dipp{$CH(CH_3)_2$}), 25.0 (s; Dipp{$CH(CH_3)_2$}), 24.9 (s; Dipp{$CH(CH_3)_2$}), 24.8 (d, J_{C-P} = 1.8 Hz; Dipp{$CH(CH_3)_2$}), 24.6 (s; Dipp{$CH(CH_3)_2$}), 24.5 (s; Dipp{$CH(CH_3)_2$}), 22.9 (s; NacNacCH_3). **^{31}P{^{1}H} NMR** (C_6D_6, 298.0 K, 202.38 MHz): δ (ppm) 67.6 (s, As*P*Ga). **IR** (ATR measurement, 64 scans, cm^{-1}): 3039 (w), 3004 (w), 2944 (s), 2909 (m), 2848 (m), 2831 (m), 2705 (vw), 1583 (vw), 1568 (vw), 1535 (m), 1513 (m), 1478 (w), 1451 (m), 1428 (s), 1372 (s), 1360 (m), 1351 (m), 1335 (w), 1308 (s9, 1282 (w), 1247 (m), 1201 (w), 1192 (w), 1179 (m), 1171 (m), 1154 (w), 1139 (w), 1102 (m), 1097 (m), 1082 (w), 1065 (m), 1049 (m), 1031 (m), 1019 (m), 994 (w), 981 (s), 964 (w), 931 (m), 891 (m), 882 (m), 841 (s), 829 (s), 791 (vs), 766 (w), 749 (vs), 718 (w), 698 (vs), 685 (m), 640 (m), 623 (w), 612 (w), 589 (s), 546 (w), 528 (m), 515 (m), 500 (w).

Figure S5. NMR and IR spectra of **5** (solvent signals indicated with asterisks).

^{1}H NMR spectrum

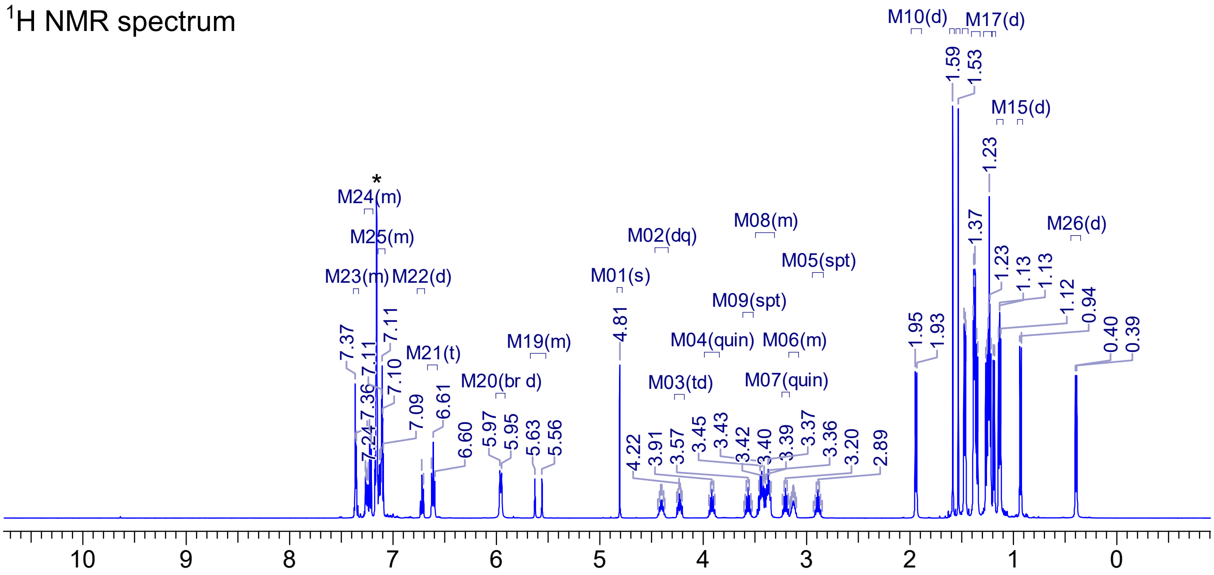

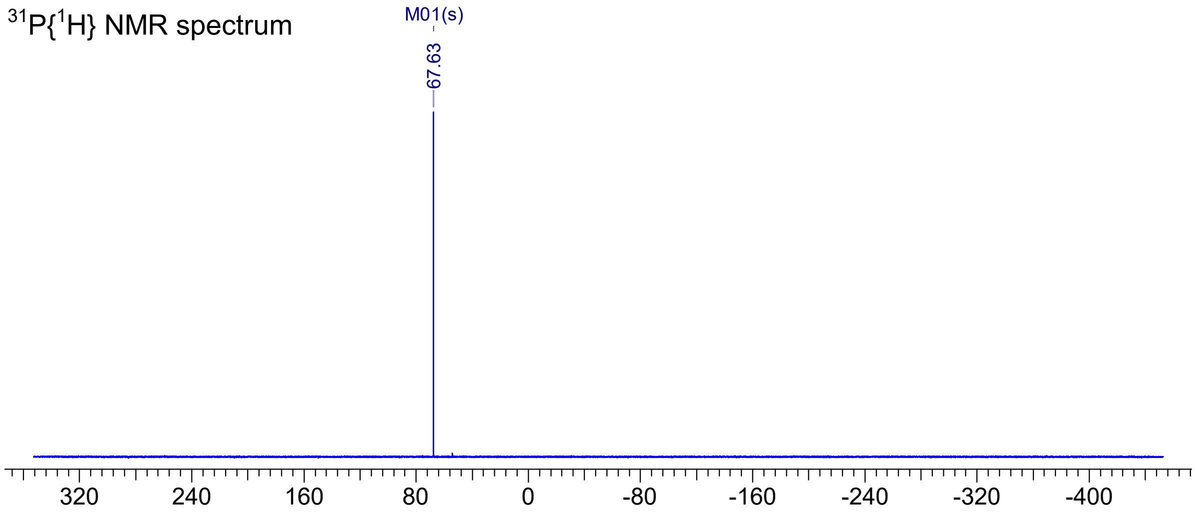
31P{1H} NMR spectrum
M01(s)
67.63
320
240
160
80
0
-80
-160
-240
-320
-400

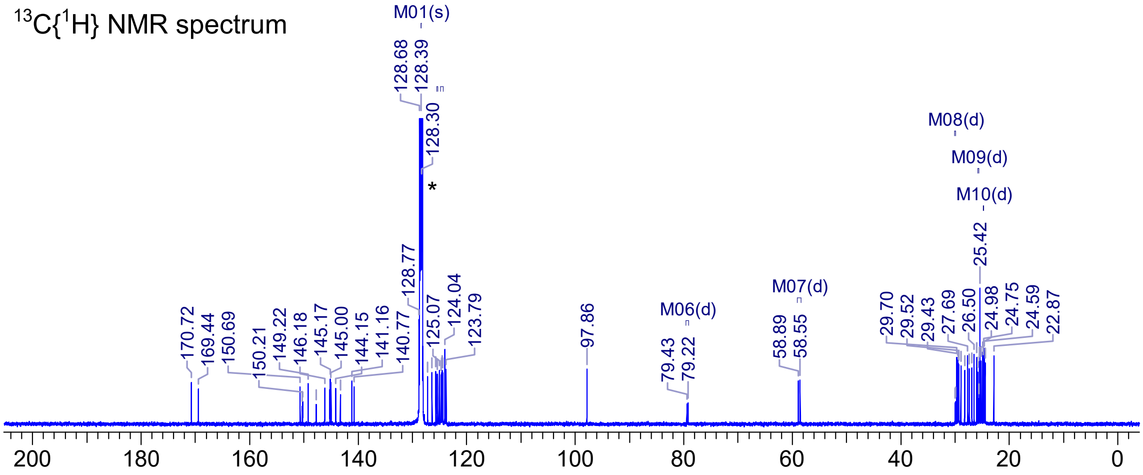
13C{1H} NMR spectrum
M01(s)
128.68
128.39
128.30
*
M08(d)
M09(d)
M10(d)
25.42
M07(d)
M06(d)
170.72
169.44
150.69
150.21
149.22
146.18
145.17
145.00
144.15
141.16
140.77
128.77
125.07
124.04
123.79
97.86
79.43
79.22
58.89
58.55
29.70
29.52
29.43
27.69
26.50
24.98
24.75
24.59
22.87
200
180
160
140
120
100
80
60
40
20
0

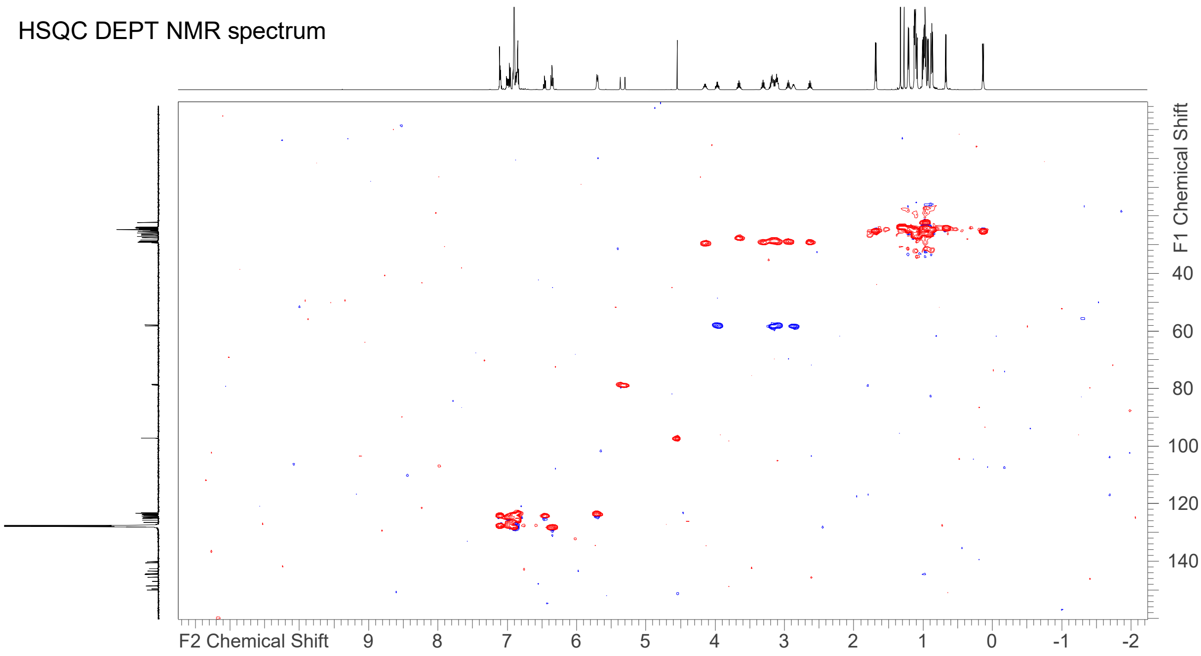
HSQC DEPT NMR spectrum
F1 Chemical Shift
40
60
80
100
120
140
F2 Chemical Shift
9
8
7
6
5
4
3
2
1
0
-1
-2

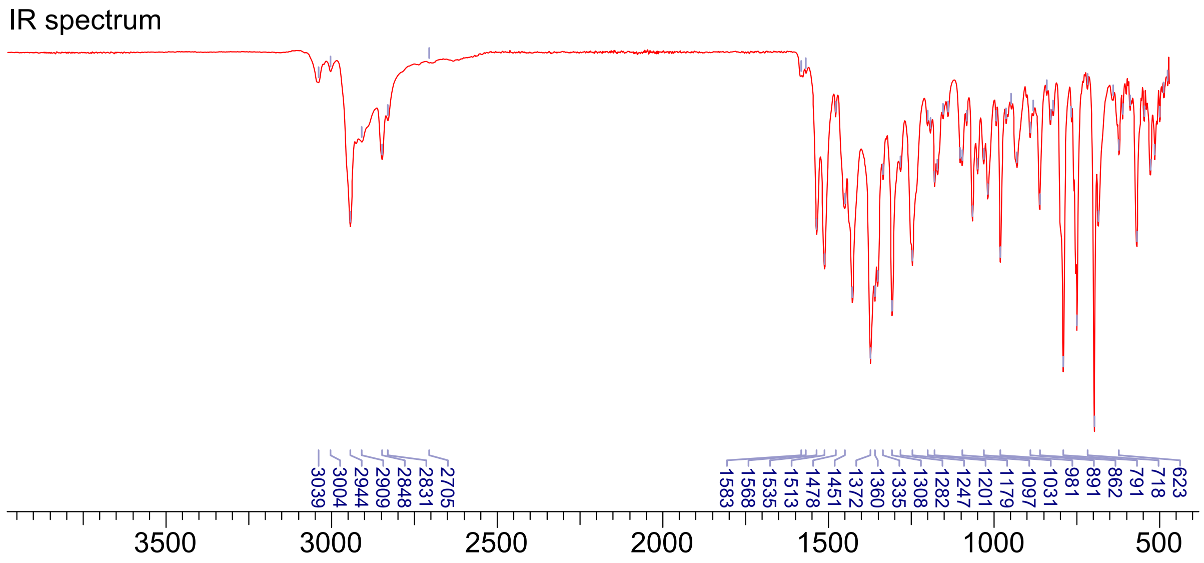

2.6 Synthesis of 7

1 (50.0 mg, 0.0520 mmol) was dissolved in C_6H_6 (0.7 ml) in a J. Young NMR tube. Dry CO_2 (2 bar) was added to the NMR tube, which was then sealed and stored at ambient temperature overnight (approx. 16 hours). Reaction progress was monitored using $^{31}P\{^1H\}$ NMR spectroscopy. When conversion to the main product in the ^{31}P NMR spectrum (−9.0 ppm) was complete, all volatiles were removed *in vacuo* (1×10^{-3} mbar, 25 °C). The resulting colourless precipitate was extracted with *n*-pentane (0.5 mL). The resulting solution was filtered into a small vial and kept at −30 °C overnight affording small, colourless crystals of **7**. The supernatant was removed with a syringe and discarded. The resulting colourless crystals were dried *in vacuo* for three hours (1×10^{-3} mbar, 25 °C). Yield: 25.6 mg, 0.0240 mmol, 47.0%.

EA for $C_{57}H_{79}AsGaN_4O_4P$ (M.W. = 1059.9 g/mol) Calcd. (found) in %: C 64.59 (65.64), H 7.51 (6.85), N 5.29 (5.07). **^{1}H NMR** (C_6D_6, 298.0 K, 499.93 MHz): δ (ppm) 7.17–7.19 (m, 1H; ArC*H*), 6.96–7.00 (m, 7H; ArC*H*), 7.03–7.14 (m, 4H; ArC*H*), 4.79 (s, 1H; Nacnac γ-H), 3.88–3.98 (m, 2H; NCH_2), 3.65–3.73 (m, 2H; Dipp{C*H*(CH_3)$_2$}), 3.52–3.62 (m, 2H; Dipp{C*H*(CH_3)$_2$}), 3.15–3.25 (m, 2H; NCH_2), 3.65–3.73 (sept, $^3J_{H-H}$ = 6.8 Hz, 4H; Dipp{C*H*(CH_3)$_2$}), 1.44 (s, 6H; NacNacCH_3), 1.32 (d, $^3J_{H-H}$ = 6.7 Hz, 12H; Dipp{CH(CH_3)$_2$}), 1.24 (d, $^3J_{H-H}$ = 6.9 Hz, 12H; Dipp{CH(CH_3)$_2$}), 1.22 (br. d, $^3J_{H-H}$ = 6.9 Hz, 6H; Dipp{CH(CH_3)$_2$}), 1.04 (d, $^3J_{H-H}$ = 6.7 Hz, 6H; Dipp{CH(CH_3)$_2$}), 0.96 (d, $^3J_{H-H}$ = 6.9 Hz, 12H; Dipp{CH(CH_3)$_2$}). **$^{13}C\{^1H\}$ NMR** (C_6D_6, 298.0 K, 125.71 MHz): δ (ppm) 177.8 (d, $^1J_{C-P}$ = 40.0 Hz; *C*O_2), 173.5 (s; Ar*C*), 150.6 (s; Ar*C*), 147.5 (s; Ar*C*), 144.7

(s; Ar*C*), 142.3 (s; Ar*C*), 137.9 (s; Ar*C*), 129.2 (s; Ar*C*H), 128.7 (s; Ar*C*H), 128.3 (s; Ar*C*H), 127.0 (s; Ar*C*H), 125.5 (s; Ar*C*H), 125.3 (s; Ar*C*H), 124.7 (s; Ar*C*H), 124.3 (s; Ar*C*H), 97.1 (s; NacNac*C*H), 59.2 (s; N*C*H_2), 29.7 (s; Dipp{*C*H(CH_3)$_2$}), 29.1 (d, $J_{C–P}$ = 7.3 Hz; Dipp{*C*H(CH_3)$_2$}), 26.1 (s; Dipp{CH(*C*H_3)$_2$}), 26.0 (s; Dipp{CH(*C*H_3)$_2$}), 25.3 (s; Dipp{CH(*C*H_3)$_2$}), 25.2 (s; Dipp{CH(*C*H_3)$_2$}), 25.1 (s; Dipp{CH(*C*H_3)$_2$}), 25.0 (s; Dipp{CH(*C*H_3)$_2$}), 23.7 (s; NacNac*C*H_3). **^{31}P{^{1}H} NMR** (C_6D_6, 298.0 K, 202.37 MHz): δ (ppm) −9.0 (s; As*P*(CO_2)$_2$). **IR** (ATR measurement, 64 scans, cm^{-1}): 3058 (vw), 2961 (s), 9272 (s), 2867 (m), 2826 (m), 1646 (s), 1632 (s), 1589 (s), 1461 (s), 1449 (s), 1384 (m), 1363 (vs), 1316 (s), 1256 (s), 1210 (vs), 1179 (m), 1105 (m), 1067 (m), 1056 (m), 1041 (m), 1028 (m), 951 (w), 935 (w), 905 (w), 890 (w), 829 (m), 801 (s), 758 (s), 728 (w), 717 (w), 708 (w), 684 (w), 639 (w), 602 (w), 585 (w), 570 (w), 551 (w), 534 (w), 530 (w), 516 (w), 506 (w), 499 (w), 490 (w), 474 (w), 461 (w).

Figure S6. NMR and IR spectra of **7** (C_6D_6 solvent signals indicated by asterisks).

^{1}H NMR spectrum

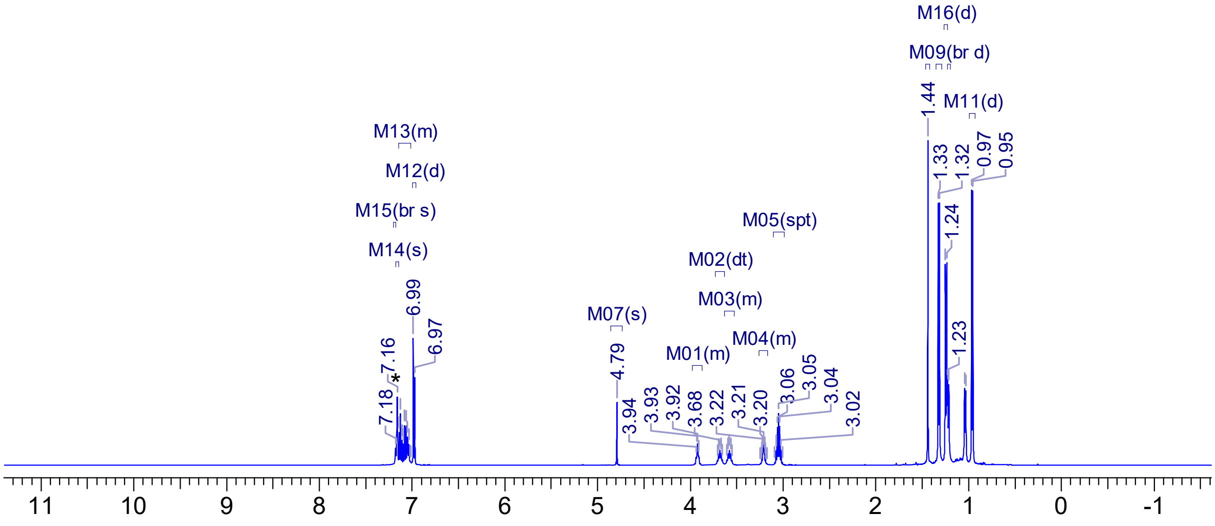

^{31}P NMR spectrum

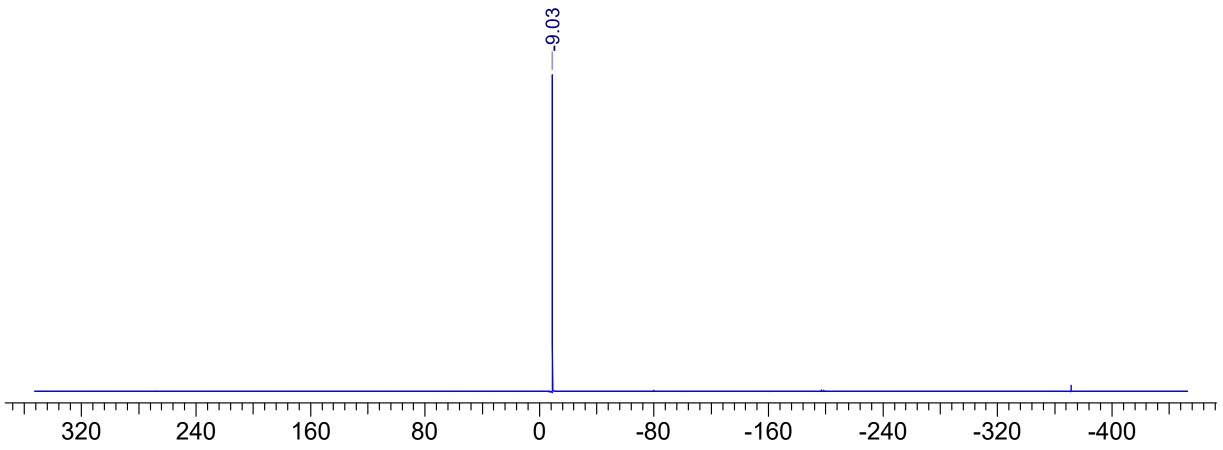

$^{13}C\{^{1}H\}$NMR spectrum

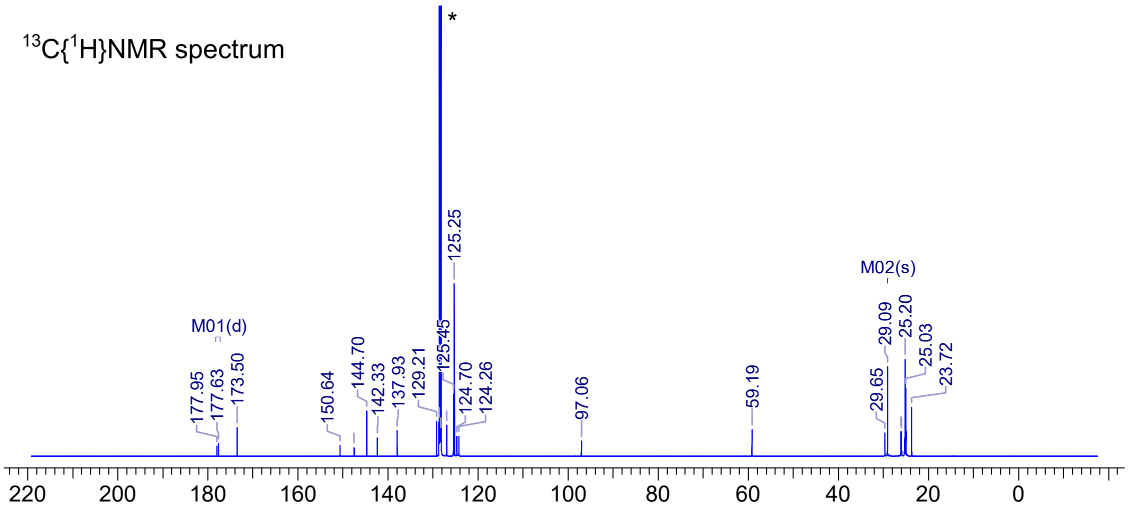

IR spectrum

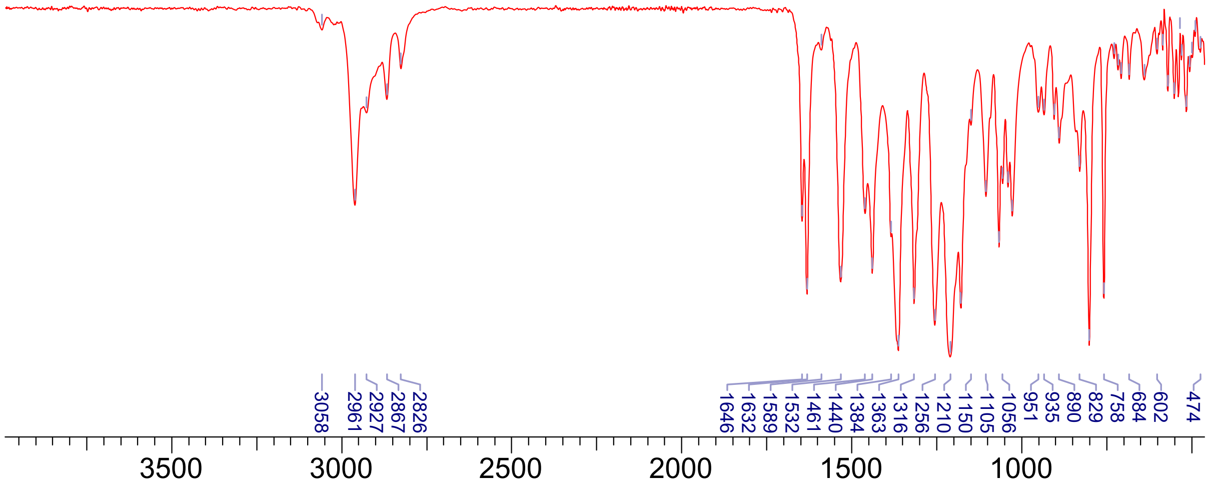

2.7 Synthesis of 8

1 (55.0 mg, 0.0570 mmol) was dissolved in C_6H_6 (0.7 ml) in a J. Young NMR tube. Dry CO_2 (2 bar) was added to the NMR tube, which was sealed and heated to 80 °C for approx. 3 days in an oil bath. The reaction progress was monitored by ^{31}P NMR spectroscopy. When the major species formed corresponded to a singlet at −371.1 ppm in the $^{31}P\{^1H\}$ NMR spectrum, all volatiles were removed *in vacuo* (1×10^{-3} mbar, 25 °C) and the colourless precipitate was extracted with *n*-hexane (0.5 mL). The resulting solution was filtered into a small vial and kept at −30 °C overnight affording small, colourless crystals of **8**. The supernatant was removed with a syringe and discarded. The resulting colourless crystals were dried *in vacuo* for three hours (1×10^{-3} mbar, 25 °C). Yield: 18.9 mg, 0.0190 mmol, 32.9%.

Notice: Due to very broad ^{1}H NMR signals at 298 K (see Figure S7), the ^{1}H NMR spectrum was recollected at 343 K, where the signals are sharper. In the ^{13}C NMR spectrum, broadening was observed for some signals as well at 298 K, but this was less pronounced than in the ^{1}H NMR spectrum. In the ^{31}P NMR spectrum at 298 K, no signal broadening was observed.

EA for $C_{56}H_{79}AsGaN_4O_2P$ (M.W. = 1015.89 g/mol) Calcd. (found) in %: C 66.21 (66.00), H 7.84 (7.34), N 5.52 (5.08). **^{1}H NMR** (C_6D_6, 343.0 K, 499.93 MHz): δ (ppm) 6.98–7.15 (m, 12H; ArC*H*), 4.89 (s, 1H; Nacnac γ-H), 4.03–4.25 (m, 4H; Dipp{C*H*(CH_3)$_2$}), 3.71–3.85 (m, 2H; NCH_2), 3.37–3.51 (m, 4H; Dipp{C*H*(CH_3)$_2$}), 3.05–3.19 (m, 2H; NCH_2), 2.14 (br. s, 3H; Dipp{CH(CH_3)$_2$} and NacNacCH_3), 1.53 (br. s; 6H; Dipp{CH(CH_3)$_2$} and NacNacCH_3), 0.98–1.50 (45 H; Dipp{CH(CH_3)$_2$} and NacNacCH_3). **$^{13}C\{^1H\}$ NMR** (C_6D_6, 298.0 K, 150.98 MHz): δ (ppm) 181.2 (d, $^1J_{C-P}$ = 92.9 Hz; P*C*O), 169.9 (br. s; Ar*C*), 150.5 (br. s; Ar*C*), 142.6 (br. s; Ar*C*), 140.8 (s; Ar*C*), 128.0 (br. s; Ar*C*H), 127.9 (br. s; Ar*C*H), 126.4 (br. s; Ar*C*H), 124.9 (br. s; Ar*C*H), 124.2 (br. s; Ar*C*H), 97.7 (s; NacNac*C*H), 59.7 (s; N*C*H_2), 38.2 (s; Dipp{*C*H(CH_3)$_2$}), 32.9 (s; Dipp{*C*H(CH_3)$_2$}), 32.0 (s; Dipp{*C*H(CH_3)$_2$}), 31.3 (s; Dipp{*C*H(CH_3)$_2$}), 30.2 (s; Dipp{*C*H(CH_3)$_2$}), 29.8 (s; Dipp{*C*H(CH_3)$_2$}), 29.5 (s; Dipp{*C*H(CH_3)$_2$}), 28.8 (br. s; Dipp{*C*H(CH_3)$_2$}), 28.2 (br. s; Dipp{*C*H(CH_3)$_2$}), 27.9 (s; Dipp{CH(*C*H_3)$_2$}), 26.0 (s; Dipp{CH(*C*H_3)$_2$}), 24.6 (s; Dipp{CH(*C*H_3)$_2$}), 22.8 (s; Dipp{CH(*C*H_3)$_2$}), 19.7 (NacNac*C*H_3). **$^{31}P\{^1H\}$ NMR** (C_6D_6, 298.0 K, 243.05 MHz): δ (ppm) −371.1 (s; Ga*P*CO). **IR** (ATR measurement, 40 scans, cm^{-1}): 3061 (w), 2961 (s), 2925 (m), 2865

(m), 2833 (w), 1922 (vs), 1588 (w), 1556 (m), 1526 (s), 1496 (m), 1460 (s), 1434 (s), 1384 (s), 1370 (s), 1359 (s), 1344 (m), 1315 (s), 1254 (m), 1209 (w), 1198 (w), 1181 (m), 1101 (m), 1077 (m), 1061 (m), 1039 (m), 1021 (s), 936 (s), 898 (w), 870 (m), 833 (m), 800 (s), 760 (s), 720 (vs), 706 (s), 644 (w), 633 (w), 605 (w), 592 (w), 574 (w), 538 (w), 517 (m), 479 (m), 464 (m).

Figure S7. NMR and IR spectra of **8**. Due to extremely broad signals in the ^{1}H NMR spectrum at 298 K, the ^{1}H NMR spectrum was recollected at 343 K. The solvent signals (C_6D_6, ^{1}H NMR: 7.16 ppm; ^{13}C NMR: 128.4 ppm) are indicated with asterisks.

^{1}H NMR spectrum at 298 K

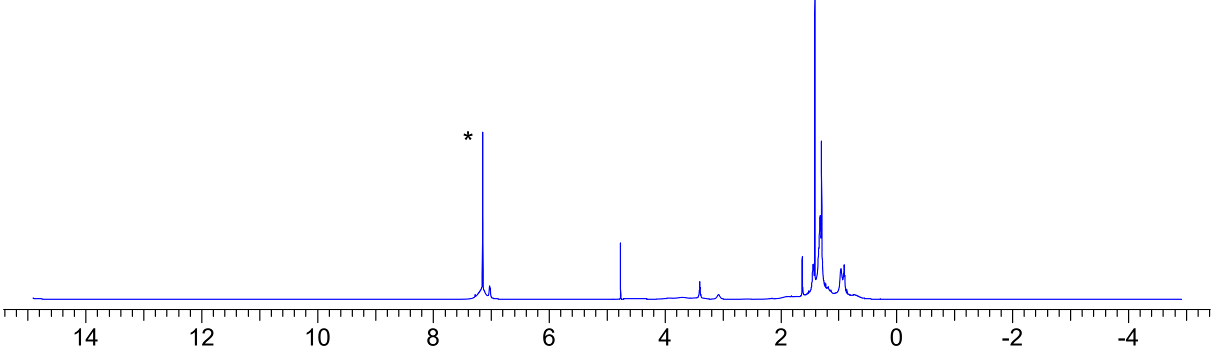

^{1}H NMR spectrum at 343 K

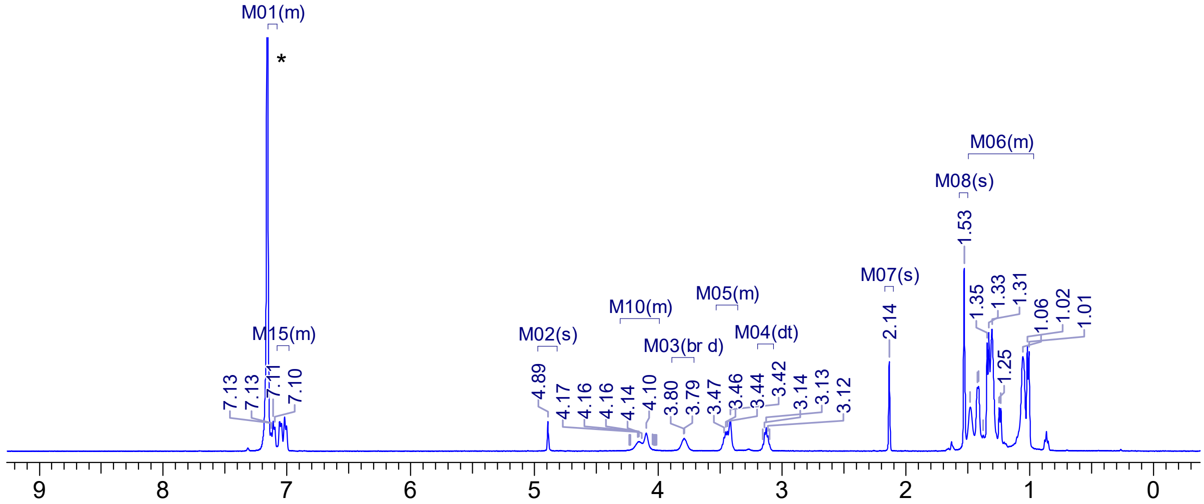

^{31}P NMR spectrum

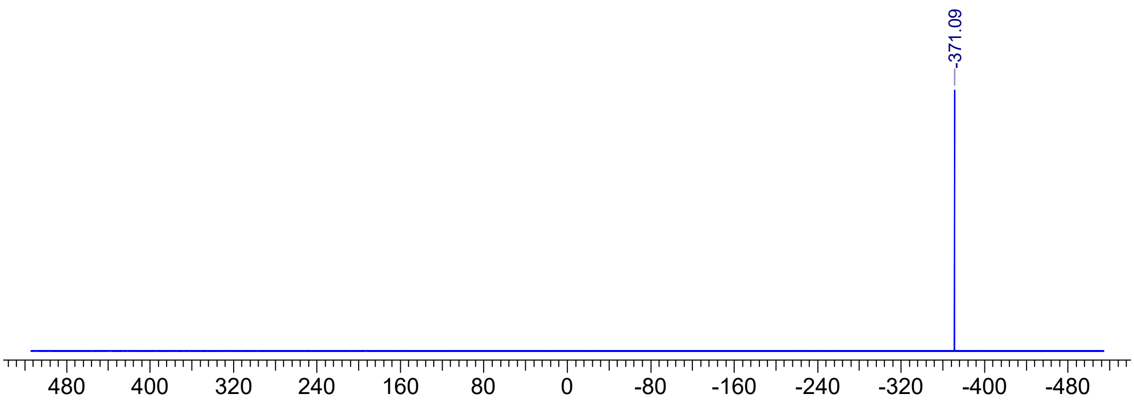

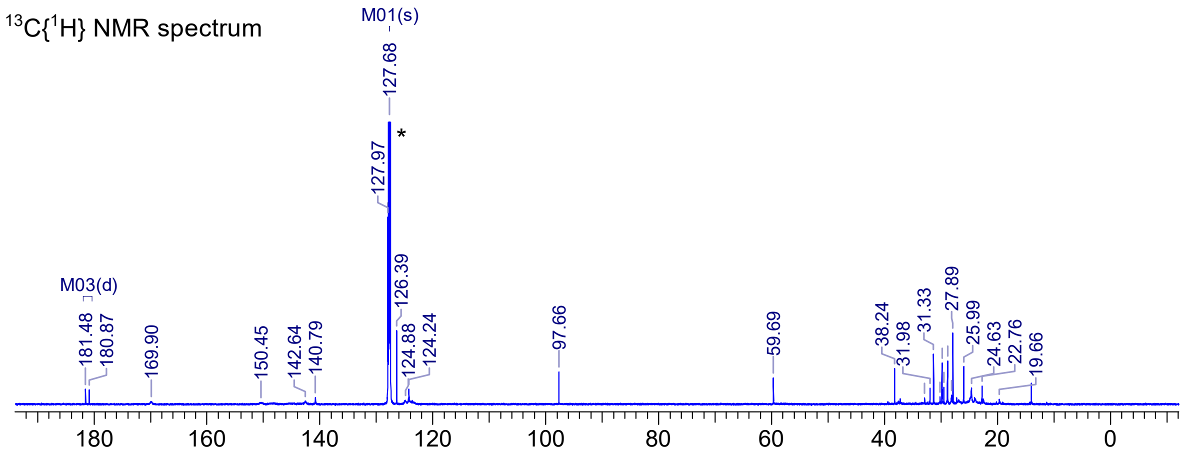

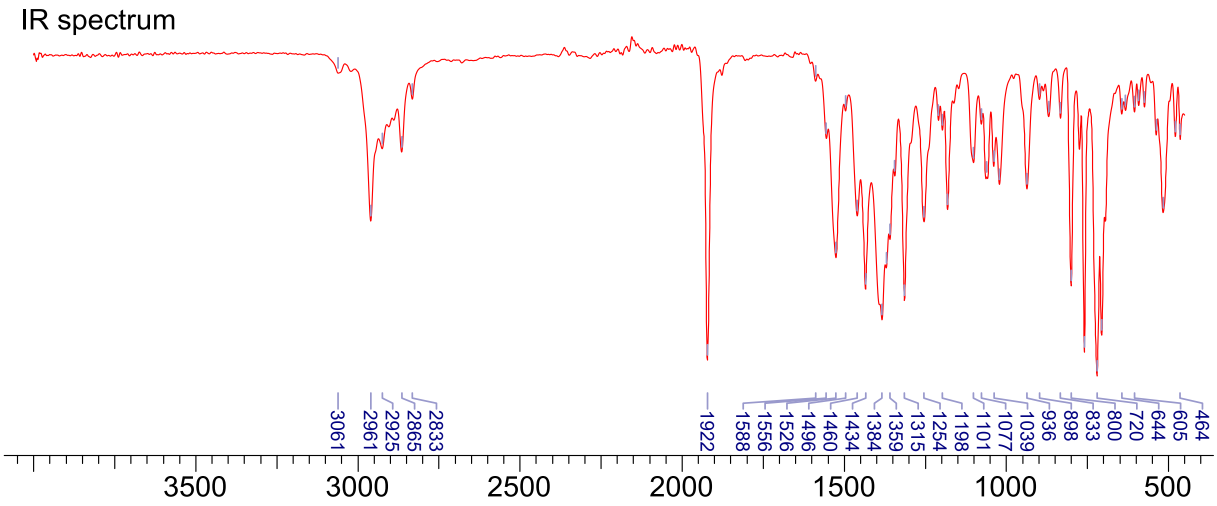

2.8 Synthesis of 9

Compound **B** (33 mg, 0.036 mmol) was dissolved in C_6H_6 (0.7 ml) in a J. Young NMR tube. CS_2 (2.1 µl, 2.7 mg, 0.036 mmol) was added to the NMR tube at ambient temperature (25 °C) resulting in an immediate colour change of the reaction mixture from red to dark purple. The tube was kept at ambient temperature for one hour after which all volatile components were removed *in vacuo* (1×10^{-3} mbar, 25 °C). The purple solid was extracted with *n*-hexane (0.5 mL) and the resulting solution was filtered into a small vial and kept at −30 °C overnight to give purple crystals of **9**. The supernatant was removed with a syringe and discarded. The resulting purple crystals were dried *in vacuo* for three hours (1×10^{-3} mbar, 25 °C). Yield: 9.2 mg, 0.0090 mmol, 26 %.

EA for $C_{56}H_{79}GaN_4P_2S_2$ (M.W. = 1004.07 g/mol) Calcd. (found) in %: C 66.99 (65.81), H 7.93 (6.99), N 5.58 (4.77). **^{1}H NMR** (C_6D_6, 298.0 K, 600.42 MHz): δ (ppm) 7.14–7.19

(m, 2H; ArC*H*), 7.04–7.10 (m, 4H; ArC*H*), 6.93–6.98 (m, 4H; ArC*H*), 6.88–6.92 (m, 2H; ArC*H*), 4.66 (s, 1H; Nacnac γ-H), 3.85–3.93 (m, 2H; NCH_2), 3.78 (sept, $^3J_{H\text{–}H}$ = 6.9 Hz, 2H; Dipp{C*H*(CH_3)$_2$}), 3.72 (sept, $^3J_{H\text{–}H}$ = 6.8 Hz, 2H; Dipp{C*H*(CH_3)$_2$}), 3.20–3.30 (m, 2H; NCH_2), 3.15 (sept, $^3J_{H\text{–}H}$ = 6.8 Hz, 2H; Dipp{C*H*(CH_3)$_2$}), 3.05 (sept, $^3J_{H\text{–}H}$ = 6.8 Hz, 2H; Dipp{C*H*(CH_3)$_2$}), 1.38 (s, 6H; NacNacCH_3), 1.36 (d, $^3J_{H\text{–}H}$ = 6.7 Hz, 6H; Dipp{CH(CH_3)$_2$}); 1.12–1.17 (m, 18H; Dipp{CH(CH_3)$_2$}), 1.04–1.10 (m, 12H; Dipp{CH(CH_3)$_2$}), 0.99 (d, $^3J_{H\text{–}H}$ = 6.9 Hz, 6H; Dipp{CH(CH_3)$_2$}), 0.92 (d, $^3J_{H\text{–}H}$ = 6.7 Hz, 6H; Dipp{CH(CH_3)$_2$}). **^{13}C{^{1}H} NMR** (C_6D_6, 298.0 K, 150.98 MHz): δ (ppm) 170.6 (s; *C*S_2), 151.9 (d, *J* = 7.3 Hz; Ar*C*), 150.1 (s; Ar*C*), 144.9 (s; Ar*C*), 143.7 (s; Ar*C*), 141.6 (s; Ar*C*), 138.2 (d; *J* = 7.1 Hz; Ar*C*), 128.7 (s; Ar*C*H), 127.9 (s; Ar*C*H), 125.4 (s; Ar*C*H), 125.2 (s; Ar*C*H), 124.8 (s; Ar*C*H), 124.7 (s; Ar*C*H), 97.5 (s; NacNac*C*H), 52.4 (s; N*C*H_2), 32.3 (s; Dipp{*C*H(CH_3)$_2$}), 29.8–30.1 (m; Dipp{*C*H(CH_3)$_2$}), 28.9 (s; Dipp{*C*H(CH_3)$_2$}), 27.9 (s; Dipp{CH(*C*H_3)$_2$}), 26.8 (s; Dipp{CH(*C*H_3)$_2$}), 26.4 (s; Dipp{CH(*C*H_3)$_2$}), 26.2 (s; Dipp{CH(*C*H_3)$_2$}), 25.5 (s; Dipp{CH(*C*H_3)$_2$}), 25.2 (s; Dipp{CH(*C*H_3)$_2$}), 24.9 (s; Dipp{CH(*C*H_3)$_2$}), 24.7 (dd, $J_{C\text{–}P}$ = 30.0 Hz, $J_{C\text{–}P}$ = 8.7 Hz; Dipp{CH(*C*H_3)$_2$}), 23.4 (s; NacNac*C*H_3). **^{31}P NMR** (C_6D_6, 298.0 K, 243.05 MHz): δ (ppm) 102.7 (d, $^1J_{P\text{–}P}$ = 614.0 Hz; *P*PGa), −287.3 (d, $^1J_{P\text{–}P}$ = 614.0 Hz; P*P*Ga). **IR** (ATR measurement, 64 scans, cm^{-1}): 3044 (w), 3004 (vw), 2942 (s), 2909 (m), 2853 (m), 1574 (w), 1544 (m), 1527 (s), 1448 (s), 1430 (s), 1388 (s), 1374 (s), 1364 (m), 1355 (s), 1334 (w), 1305 (s), 1283 (m), 1187 (m), 1171 (m), 1158 (s), 1144 (s), 1099 (m), 1066 (s), 1052 (m), 1021 (s), 976 (m), 934 (m), 881 (m), 852 (m), 798 (s), 792 (s), 763 (m), 755 (vs), 726 (w), 718 (w), 702 (w), 695 (w), 668 (s), 640 (m), 635 (m), 619 (w), 598 (m), 548 (s), 538 (s), 528 (m), 519 (s), 508 (m), 490 (m).

Figure S8. NMR and IR spectra of **9**. The crystals were dried *in vacuo* for 3 h (1×10^{-3} mbar, 25 °C), however, the sample still contains slight traces of *n*-pentane (^{1}H NMR: 0.87 ppm, 1.23 ppm; ^{13}C NMR: 14.3 ppm, 22.7 ppm, 34.5 ppm), indicated with asterisks. The solvent signals (C_6D_6, ^{1}H NMR: 7.16 ppm; ^{13}C NMR: 128.4 ppm) are also indicated with asterisks.

^{1}H NMR spectrum

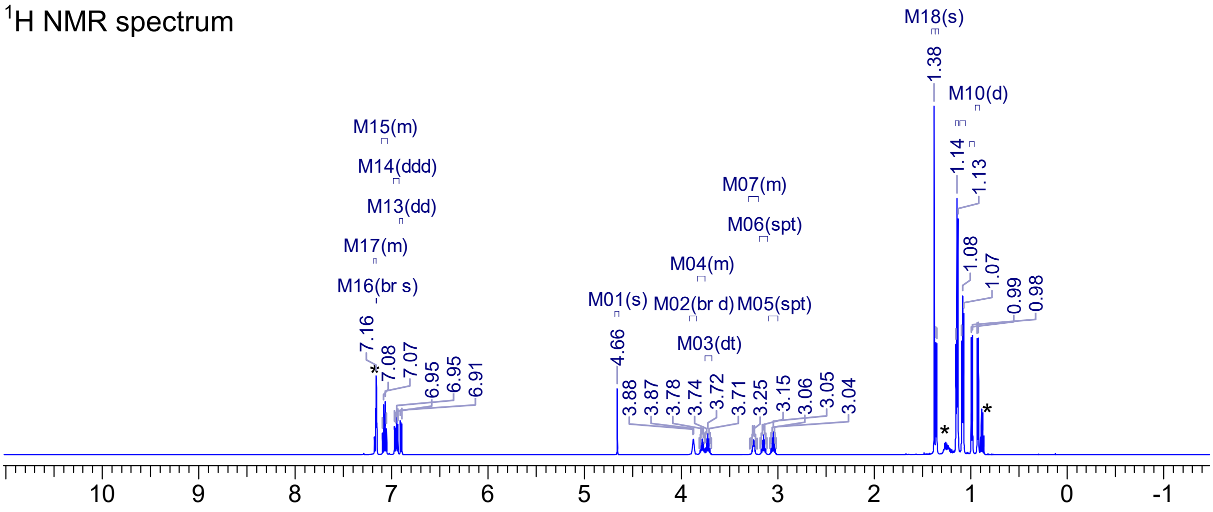

^{31}P NMR spectrum

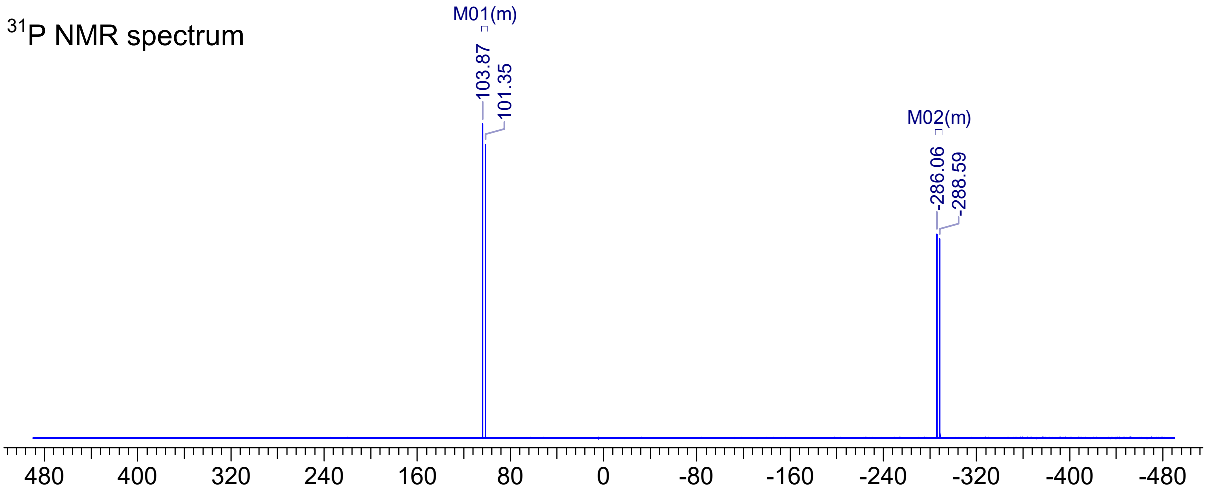

^{13}C{^{1}H} NMR spectrum

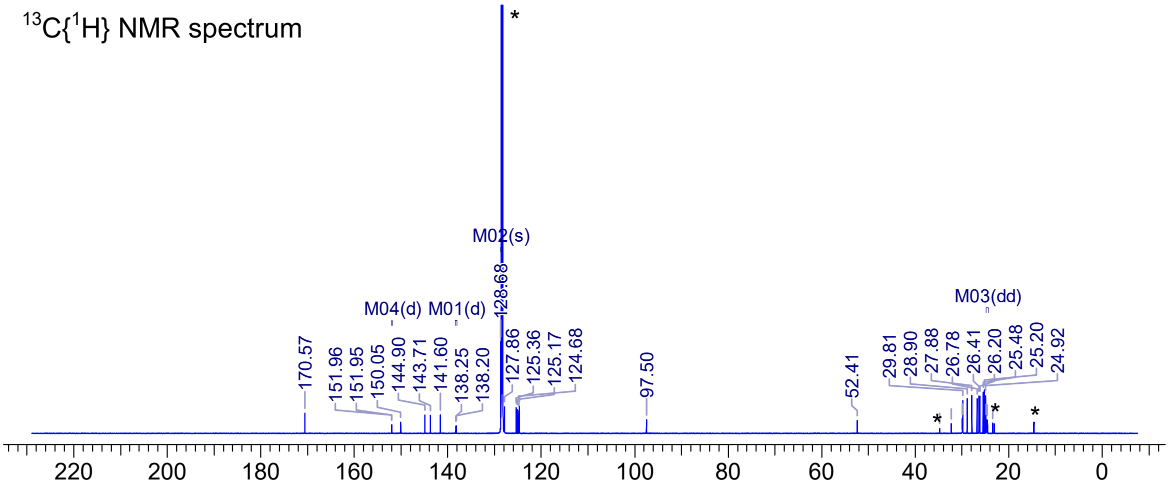

IR spectrum

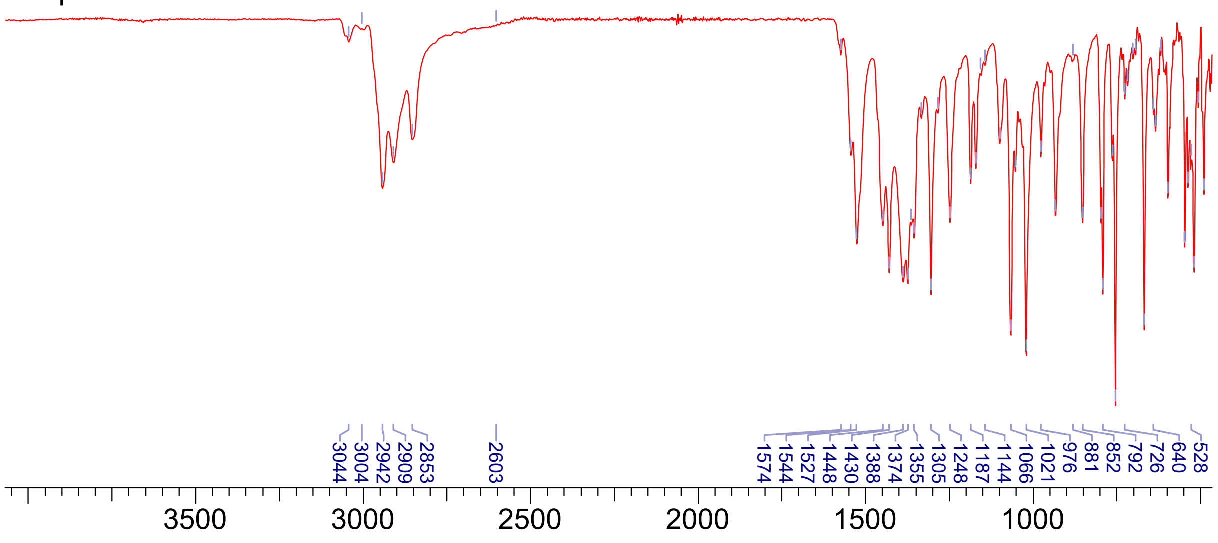

2.9 Synthesis of 10

1 (50.0 mg, 0.0520 mmol) was dissolved in C_6H_6 (0.7 ml) in a J. Young NMR tube. An excess of CS_2 (1 drop) was added to the NMR tube at ambient temperature (25 °C) after which the colour of the reaction mixture changed from dark red to orange. The tube was kept at ambient temperature for one hour and afterwards all volatile components were removed *in vacuo* (1×10^{-3} mbar, 25 °C). The orange precipitate was extracted with *n*-hexane (0.5 mL) and the resulting slightly turbid solution was filtered into a small vial. Storage of this solution at ambient temperature overnight afforded orange crystals of **10** (suitable for X-ray diffraction). The supernatant was removed with a syringe and discarded. The resulting orange crystals were dried *in vacuo* for three hours (1×10^{-3} mbar, 25 °C). Yield: 29.4 mg, 0.0140 mmol, 54.5 % (of dimer, max. yield is 100%).

EA for $C_{112}H_{158}As_2Ga_2N_8P_2S_4$ (M.W. = 2096.04 g/mol) Calcd. (found) in %: C 64.18 (64.42), H 7.60 (8.23), N 5.35 (4.93). **^{1}H NMR** (C_6D_6, 298.0 K, 600.42 MHz): δ (ppm) 7.25–7.38 (m, 13H; ArC*H*), 7.19 ($^3J_{H–H}$ = 7.5 Hz, 1H; ArC*H*), 7.12–7.15 (m, 2H; ArC*H*), 7.08 ($^3J_{H–H}$ = 7.5 Hz, 1H; ArC*H*), 6.94–7.05 (m, 6H; ArC*H*), 6.79 ($^3J_{H–H}$ = 7.3 Hz, 1H; ArC*H*), 4.80 (s, 1H; NacNac γ-H), 4.67 (s, 1H; NacNac γ-H), 4.30–4.40 (m, 1H; NCH_2), 4.20–4.28 (m, 1H; NCH_2), 4.02–4.11 (m, 2H; Dipp{C*H*(CH_3)$_2$}), 3.85–4.01 (m, 4H; Dipp{C*H*(CH_3)$_2$} and NCH_2), 3.60–3.65 (m, 1H; Dipp{C*H*(CH_3)$_2$}), 3.53–3.58 (m, 2H; Dipp{C*H*(CH_3)$_2$} and NCH_2), 3.40–3.50 (m, 4H; Dipp{C*H*(CH_3)$_2$} and NCH_2), 3.30–3.40

(m, 1H; NCH_2), 3.20–3.30 (m, 2H; Dipp{C*H*(CH_3)$_2$}), 3.05–3.10 (m, 3H; Dipp{C*H*(CH_3)$_2$}), 2.90–3.00 (m, 3H; Dipp{C*H*(CH_3)$_2$} and NCH_2), 1.86 (d, $^3J_{H–H}$ = 6.5 Hz, 3H; NacNacCH_3), 1.81 (d, $^3J_{H–H}$ = 6.5 Hz, 3H; NacNacCH_3), 1.68 (d, $^3J_{H–H}$ = 6.7 Hz, 3H; Dipp{CH(CH_3)$_2$}), 1.60–1.64 (m, 6H; NacNacCH_3), 1.59 (d, $^3J_{H–H}$ = 6.9 Hz, 3H; Dipp{CH(CH_3)$_2$}), 1.52–1.56 (m, 6H; Dipp{CH(CH_3)$_2$}), 1.44–1.48 (m, 21H; Dipp{CH(CH_3)$_2$}), 1.40 (d, $^3J_{H–H}$ = 6.7 Hz, 3H; Dipp{CH(CH_3)$_2$}), 1.32–1.37 (m, 6H; Dipp{CH(CH_3)$_2$}), 1.30 (d, $^3J_{H–H}$ = 6.9 Hz, 3H; Dipp{CH(CH_3)$_2$}), 1.27 (d, $^3J_{H–H}$ = 6.7 Hz, 3H; Dipp{CH(CH_3)$_2$}), 1.52–1.56 (m, 9H; Dipp{CH(CH_3)$_2$}), 1.14–1.18 (m, 6H; Dipp{CH(CH_3)$_2$}), 1.08–1.12 (m, 9H; Dipp{CH(CH_3)$_2$}), 0.99 (d, $^3J_{H–H}$ = 6.7 Hz, 3H; Dipp{CH(CH_3)$_2$}), 0.97 (d, $^3J_{H–H}$ = 6.7 Hz, 3H; Dipp{CH(CH_3)$_2$}), 0.94 (d, $^3J_{H–H}$ = 6.7 Hz, *3*H; Dipp{CH(CH_3)$_2$}), 0.92 (d, $^3J_{H–H}$ = 6.7 Hz, *3*H; Dipp{CH(CH_3)$_2$}), 0.61 (d, $^3J_{H–H}$ = 6.7 Hz, 3H; Dipp{CH(CH_3)$_2$}), 0.40–0.47 (m, 6H; Dipp{CH(CH_3)$_2$}), 0.21 (d, $^3J_{H–H}$ = 6.5 Hz, 3H; Dipp{CH(CH_3)$_2$}). **^{13}C{^{1}H} NMR** (C_6D_6, 298.0 K, 150.98 MHz): δ (ppm) 169.8 (s; Ar*C*), 169.5 (s; Ar*C*), 169.4 (s; Ar*C*), 168.8 (s; Ar*C*), 150.7 (s; Ar*C*), 150.0 (d, $^3J_{C–P}$ = 6.5 Hz; Ar*C*), 149.2 (br. s; Ar*C*), 148.6 (br. s; Ar*C*), 148.5 (s; Ar*C*), 146.3 (s; Ar*C*), 146.2 (s; Ar*C*), 146.0 (s; Ar*C*), 145.3 (s; Ar*C*), 145.1 (s; Ar*C*), 144.6 (s; Ar*C*), 144.3 (s; Ar*C*), 144.2 (s; Ar*C*), 144.1 (s; Ar*C*), 143.9 (s; Ar*C*), 143.4 (s; Ar*C*), 142.6 (s; Ar*C*), 142.0 (s; Ar*C*), 141.7 (s; Ar*C*), 141.5 (s; Ar*C*), 140.4 (s; Ar*C*), 139.9 (s; Ar*C*), 127.2 (s; Ar*C*H), 126.7 (s; Ar*C*H), 126.5 (s; Ar*C*H), 125.7 (s; Ar*C*H), 125.5 (s; Ar*C*H), 125.2 (s; Ar*C*H), 125.1 (s; Ar*C*H), 124.9 (br. s; Ar*C*H), 124.7 (s; Ar*C*H), 124.3 (s; Ar*C*H), 124.1 (d, $^3J_{C–P}$ = 5.5 Hz; Ar*C*H), 124.0 (s; Ar*C*H), 123.7 (s; Ar*C*H), 123.5 (s; Ar*C*H), 123.4 (s; Ar*C*H), 123.3 (s; Ar*C*H), 122.8 (s; Ar*C*H), 122.4 (s; Ar*C*H), 121.5 (m; CS_2), 99.3 (s; NacNac*C*H), 97.0 (s; NacNac*C*H), 59.5 (s; NCH_2), 59.0 (s; NCH_2), 58.0 (s; NCH_2), 56.4 (s; NCH_2), 29.3 (d; $J_{C–P}$ = 7.0 Hz; Dipp{*C*H(CH_3)$_2$}), 29.2 (br. s; Dipp{*C*H(CH_3)$_2$}), 29.1 (s; Dipp{*C*H(CH_3)$_2$}), 29.0 (br. d, $J_{C–P}$ = 7.6 Hz; Dipp{*C*H(CH_3)$_2$}), 28.9 (br. s; Dipp{*C*H(CH_3)$_2$}), 28.8 (s; Dipp{*C*H(CH_3)$_2$}), 28.7 (m; Dipp{*C*H(CH_3)$_2$}), 28.6 (m; Dipp{*C*H(CH_3)$_2$}), 28.4 (s; Dipp{*C*H(CH_3)$_2$}), 28.2 (br. d, $J_{C–P}$ = 3.8 Hz; Dipp{*C*H(CH_3)$_2$}), 27.7 (s; Dipp{CH(CH_3)$_2$}), 27.5 (d, $J_{C–P}$ = 6.5 Hz; Dipp{CH(CH_3)$_2$}), 27.4 (d, $J_{C–P}$ = 10.9 Hz; Dipp{CH(CH_3)$_2$}), 26.8 (s; Dipp{CH(CH_3)$_2$}), 26.7 (s; Dipp{CH(CH_3)$_2$}), 26.6 (s; Dipp{CH(CH_3)$_2$}), 26.5 (s; Dipp{CH(CH_3)$_2$}), 26.4 (s; Dipp{CH(CH_3)$_2$}), 25.8 (s; Dipp{CH(CH_3)$_2$}), 25.7 (s; Dipp{CH(CH_3)$_2$}), 25.5 (s; Dipp{CH(CH_3)$_2$}), 25.3 (d, $J_{C–P}$ = 15.3 Hz; Dipp{CH(CH_3)$_2$}), 25.1 (d, $J_{C–P}$ = 4.3 Hz; Dipp{CH(CH_3)$_2$}), 24.8 (s; Dipp{CH(CH_3)$_2$}), 24.7 (d, $J_{C–P}$ = 2.2 Hz; Dipp{CH(CH_3)$_2$}), 24.6 (s; Dipp{CH(CH_3)$_2$}), 24.5 (s; Dipp{CH(CH_3)$_2$}), 24.4 (d, $J_{C–P}$ = 7.1 Hz; Dipp{CH(CH_3)$_2$}), 24.2 (s;

Dipp{CH(CH_3)$_2$}), 24.1 (s; Dipp{CH(CH_3)$_2$}), 23.8 (s; Dipp{CH(CH_3)$_2$}), 23.5 (s; Dipp{CH(CH_3)$_2$}), 23.4 (s; Dipp{CH(CH_3)$_2$}), 25.3 (d, $J_{C–P}$ = 4.9 Hz; Dipp{CH(CH_3)$_2$}), 23.1 (s; NacNacCH_3), 22.6 (s; NacNacCH_3). **^{31}P NMR** (C_6D_6, 298.0 K, 243.05 MHz): δ (ppm) −25.9 (s; As*P*(S)Ga), −52.5 (s; As*P*(S)Ga). **IR** (ATR measurement, 64 scans, cm^{-1}): 3041 (w), 3002 (s), 2942 (m), 2908 (m), 2849 (m), 2809 (w), 1578 (w), 1543 (m), 1514 (s), 1452 (s), 1425 (s), 1375 (vs), 1352 (m), 1307 (s), 1247 (m), 1198 (m), 1168 (m), 1099 (m), 1061 (m), 1049 (m), 1034 (m), 1015 (m), 929 (m), 892 (m), 856 (m), 822 (m), 792 (vs), 751 (vs), 725 (w), 719 (w), 702 (w), 685 (w), 628 (m), 610 (w), 602 (w), 598 (w), 585 (w), 579 (w), 568 (w), 553 (w), 548 (w), 543 (w), 532 (m), 525 (w), 515 (w), 505 (m), 496 (w).

Figure S9. NMR and IR spectra of **10**. The crystals were dried *in vacuo* for 3 h (1×10^{-3} mbar, 40 °C), however, the sample still contains slight traces of *n*-pentane (^{1}H NMR: 0.87 ppm, 1.23 ppm; ^{13}C NMR: 14.3 ppm, 22.7 ppm, 34.5 ppm), indicated with asterisks. The solvent signals (C_6D_6, ^{1}H NMR: 7.16 ppm; ^{13}C NMR: 128.4 ppm) are also indicated with asterisks.

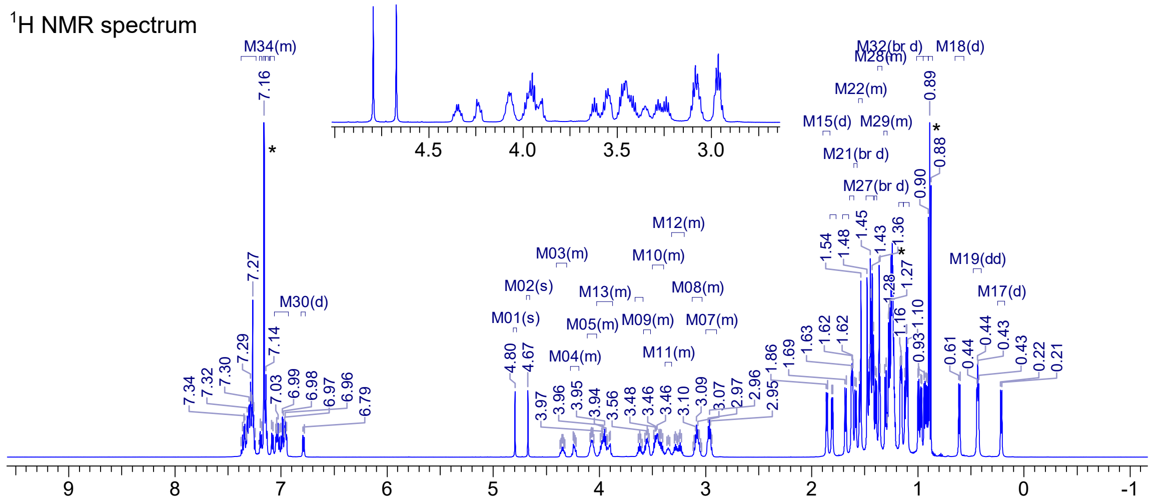

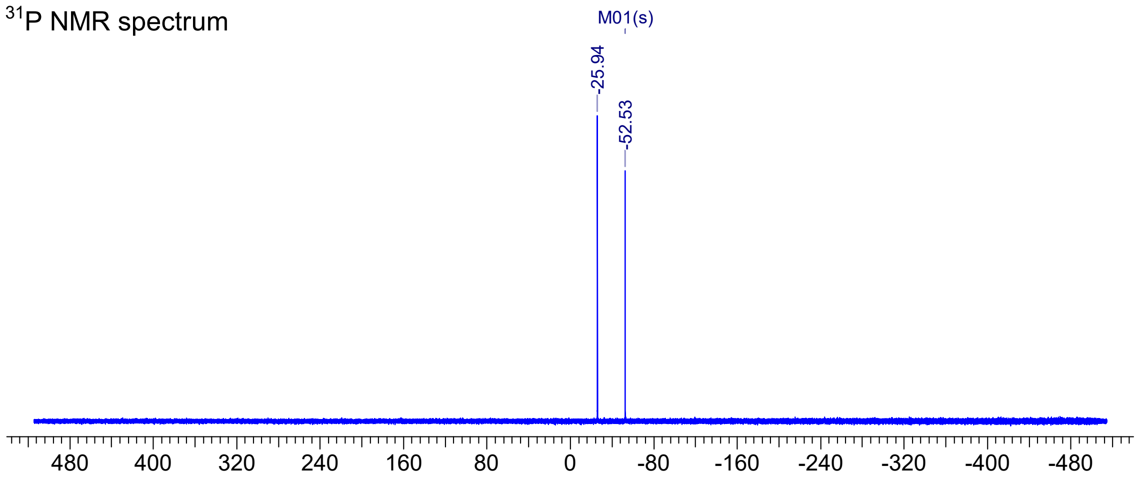

^{31}P NMR spectrum
M01(s)
-25.94
-52.53
480
400
320
240
160
80
0
-80
-160
-240
-320
-400
-480

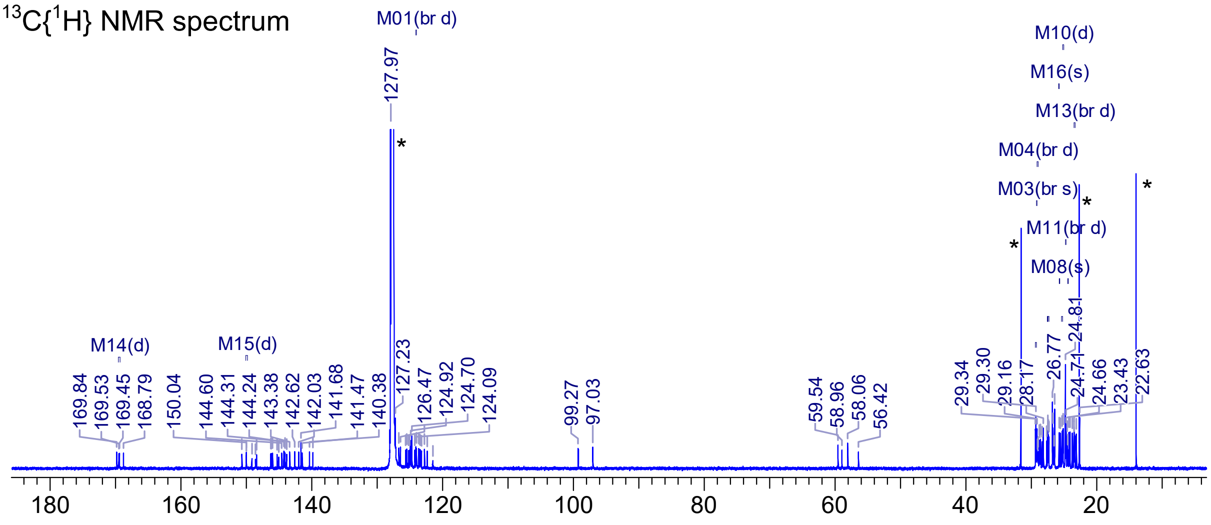

^{13}C{^{1}H} NMR spectrum
M01(br d)
127.97
M10(d)
M16(s)
M13(br d)
M04(br d)
M03(br s)
M11(br d)
M08(s)
M14(d)
M15(d)
169.84
169.53
169.45
168.79
150.04
144.60
144.31
144.24
143.38
142.62
142.03
141.68
141.47
140.38
127.23
126.47
124.92
124.70
124.09
99.27
97.03
59.54
58.96
58.06
56.42
29.34
29.30
29.16
28.17
26.77
24.71
24.81
24.66
23.43
22.63
*
180
160
140
120
100
80
60
40
20

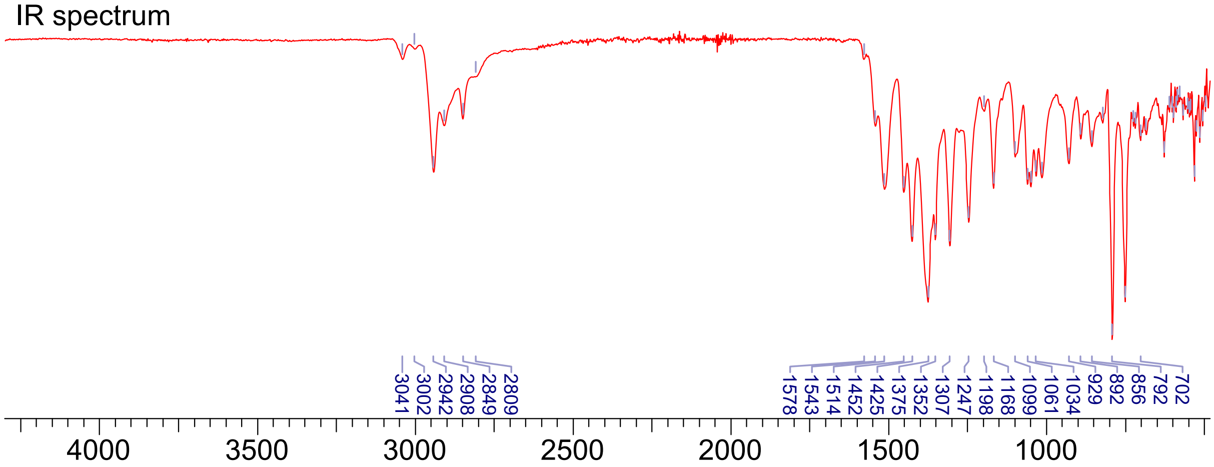

IR spectrum
3041
3002
2942
2908
2849
2809
1578
1543
1514
1452
1425
1375
1352
1307
1247
1198
1168
1099
1061
1034
929
892
856
792
702
4000
3500
3000
2500
2000
1500
1000

2.10 Synthesis of 11

1 (60.0 mg, 0.0610 mmol) was dissolved in C_6H_6 (0.7 ml) in a J. Young NMR tube. Dry COS (1 bar) was added to the NMR tube, which was then sealed and stored at room temperature for approx. 1 hour. Reaction progress was monitored using $^{31}P\{^1H\}$ NMR spectroscopy. When the spectrum revealed a singlet at −371.1 ppm, the volatile components were removed *in vacuo* ($1{\times}10^{-3}$ mbar, 25 °C) and the yellow precipitate was extracted with *n*-pentane/toluene (0.3 mL/0.3 mL). The resulting solution was filtered into a small vial and kept at room temperature overnight affording small, colourless crystals of **11**. The supernatant was removed with a syringe, discarded and the resulting colourless crystals were dried *in vacuo* for three hours ($1{\times}10^{-3}$ mbar, 25 °C). Yield: 27.3 mg, 0.0270 mmol, 43.4%.

EA for $C_{56}H_{79}AsGaN_4OSP$ (M.W. = 1031.95 g/mol) Calcd. (found) in %: C 65.18 (65.63), H 7.72 (7.76), N 5.43 (5.14). **^{1}H NMR** (C_6D_6, 298.2 K, 600.16 MHz): δ (ppm) 7.17–7.21 (m, 2H; ArC*H*), 7.08–7.11 (m, 3H; ArC*H*), 7.00–7.05 (m, 5H; ArC*H*), 6.96–6.99 (m, 2H; ArC*H*), 4.83 (s, 1H; NacNac γ-H), 3.81–3.90 (m, 2H; Dipp{C*H*(CH_3)$_2$}), 3.65–3.75 (m, 4H; Dipp{C*H*(CH_3)$_2$} and NCH_2), 3.50–3.60 (m, 2H; Dipp{C*H*(CH_3)$_2$}), 3.15–3.25 (m, 4H; Dipp{C*H*(CH_3)$_2$} and NCH_2); 1.54 (d, 6H, $^3J_{H–H}$ = 6.9 Hz; Dipp{CH(CH_3)$_2$}), 1.39 (s, 6H; NacNacCH_3), 1.54 (d, 6H, $^3J_{H–H}$ = 6.9 Hz; Dipp{CH(CH_3)$_2$}), 1.42 (d, 6H, $^3J_{H–H}$ = 6.9 Hz; Dipp{CH(CH_3)$_2$}), 1.26 (d, 6H, $^3J_{H–H}$ = 6.5 Hz, Dipp{CH(CH_3)$_2$}), 1.23 (d, 6H, $^3J_{H–H}$ = 6.9 Hz, Dipp{CH(CH_3)$_2$}), 1.11 (d, 6H, $^3J_{H–H}$ = 6.9 Hz; Dipp{CH(CH_3)$_2$}), 1.06 (d, 6H, $^3J_{H–H}$ = 6.9 Hz; Dipp{CH(CH_3)$_2$}), 0.58 (d, 6H, $^3J_{H–H}$ = 6.9 Hz; Dipp{CH(CH_3)$_2$}), 0.53 (d, 6H, $^3J_{H–H}$ = 6.9 Hz; Dipp{CH(CH_3)$_2$}). **^{13}C{^{1}H} NMR** (C_6D_6, 298.2 K, 150.91 MHz): δ (ppm) 172.0 (s; P*C*O), 150.6 (s; Ar*C*), 148.0 (s; Ar*C*), 146.3 (s; Ar*C*), 143.8 (s; Ar*C*), 141.6 (s; Ar*C*), 140.7 (s; Ar*C*), 129.7 (br. s; Ar*C*H), 128.9 (br. s; Ar*C*H), 127.3 (br. s; Ar*C*H), 126.0 (br. s; Ar*C*H), 125.2 (br. s; Ar*C*H), 124.8 (br. s; Ar*C*H), 124.3 (br. s; Ar*C*H), 101.6 (s; NacNac*C*H), 58.6 (s; N*C*H_2), 29.8 (s; Dipp{*C*H(CH_3)$_2$}), 29.4 (s; Dipp{*C*H(CH_3)$_2$}), 28.8 (s; Dipp{*C*H(CH_3)$_2$}), 28.4 (s; Dipp{*C*H(CH_3)$_2$}), 27.3 (s; Dipp{CH(*C*H_3)$_2$}), 26.5 (s; Dipp{CH(*C*H_3)$_2$}), 26.2 (s; Dipp{CH(*C*H_3)$_2$}), 25.6 (s; Dipp{CH(*C*H_3)$_2$}), 25.3 (s; Dipp{CH(*C*H_3)$_2$}), 25.1 (s; Dipp{CH(*C*H_3)$_2$}), 24.9 (s; Dipp{CH(*C*H_3)$_2$}), 24.7 (s; Dipp{CH(*C*H_3)$_2$}), 24.4 (s; Dipp{CH(*C*H_3)$_2$}), 21.8 (s; NacNac*C*H_3). **^{31}P NMR** (C_6D_6, 298.2 K, 242.95 MHz): δ (ppm) −365.4 (s, Ga*P*CO). **IR** (ATR measurement, 40 scans, cm^{-1}): 2957 (m), 2925 (m), 2866 (w), 2833 (w), 2653 (vw), 1907 (vs), 1587 (w), 1526 (s), 1456 (m), 1438 (m),

1377 (s), 1360 (m), 1318 (m), 1254 (m), 1209 (w), 1175 (w), 1103 (m), 1071 (m), 1057 (m), 1040 (m), 1020 (m), 963 (w), 936 (w), 908 (w), 883 (w), 865 (w), 836 (w), 800 (s), 777 (w), 757 (s), 722 (vw), 708 (vw), 640 (w), 594 (w), 575 (w), 553 (vw), 536 (w), 518 (m), 477 (vw), 430 (m), 408 (m).

Figure S10. NMR and IR spectra of **11**. The crystals were dried *in vacuo* for 3 h (1×10^{-3} mbar, 25 °C), however, the sample still contains slight traces of toluene (^{1}H NMR: 2.11 ppm, 7.02 ppm, 7.13 ppm), indicated with asterisks. The signal at 0.30 ppm stems from traces of grease, as indicated with asterisks. The solvent signals (C_6D_6, ^{1}H NMR: 7.16 ppm; ^{13}C NMR: 128.4 ppm) are also indicated with asterisks.

^{1}H NMR spectrum

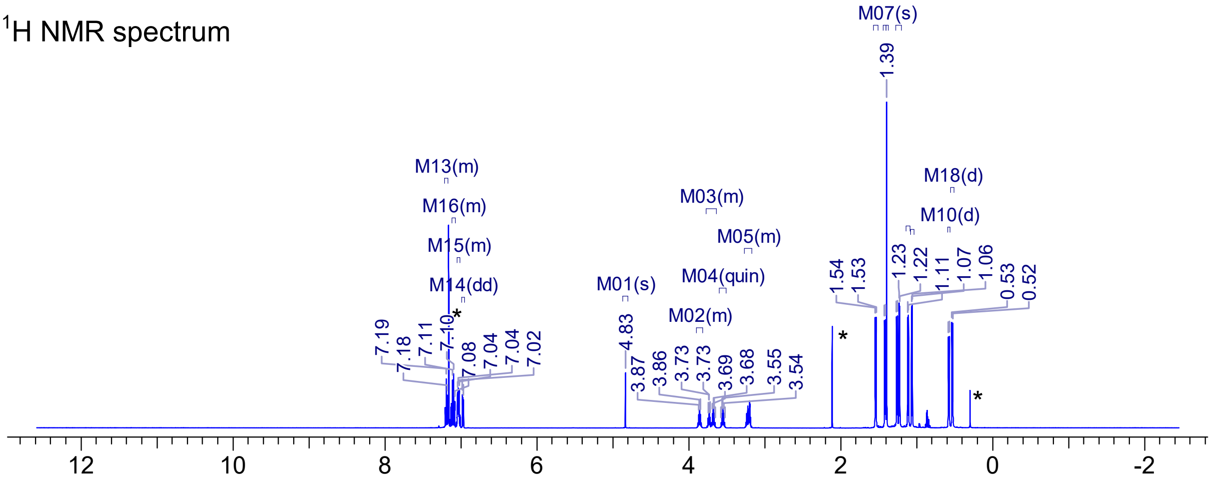

^{31}P NMR spectrum

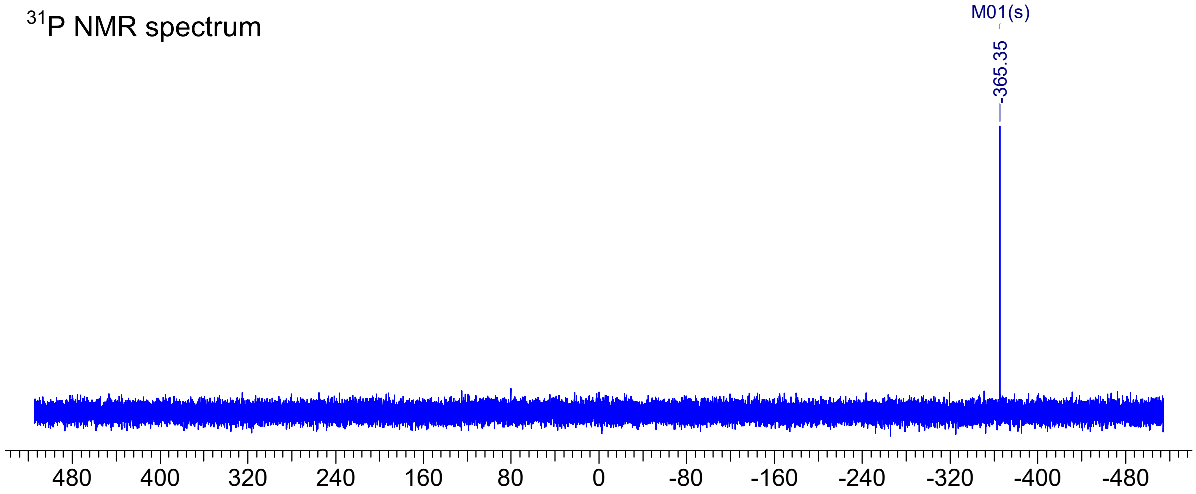

$^{13}C\{^1H\}$ NMR spectrum

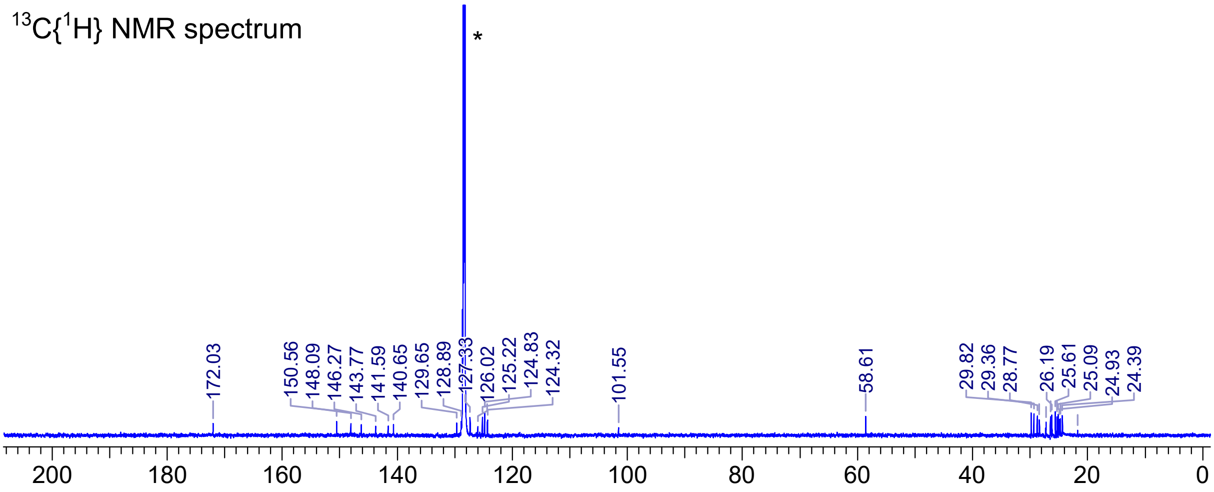

COSY NMR spectrum

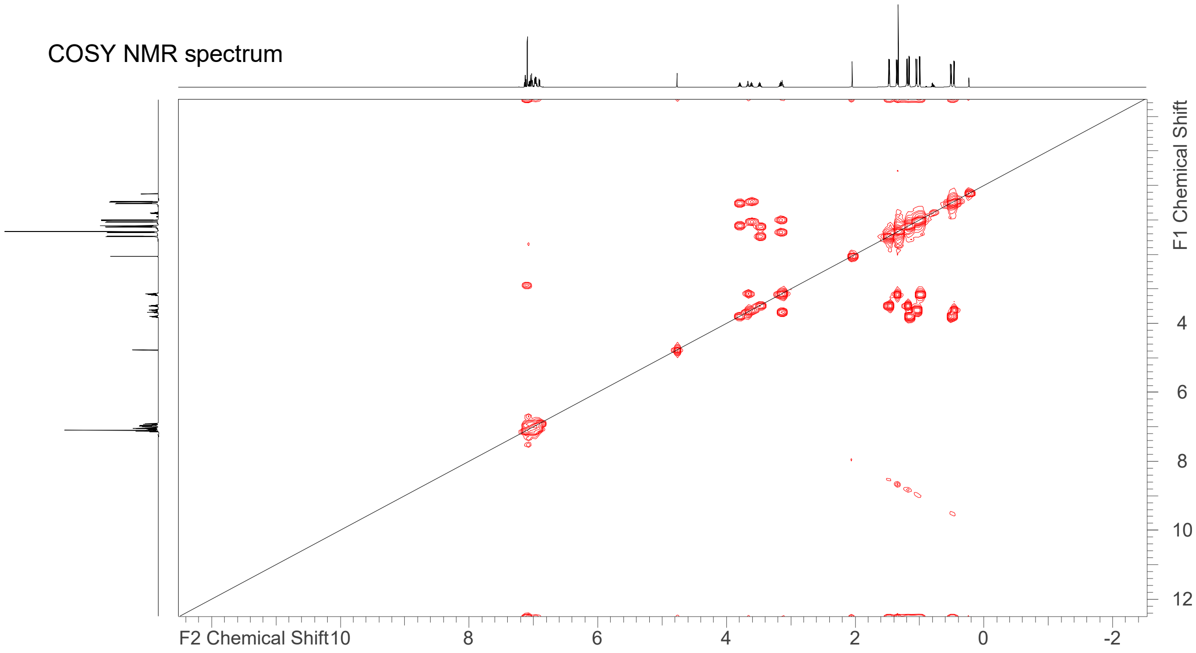

IR spectrum

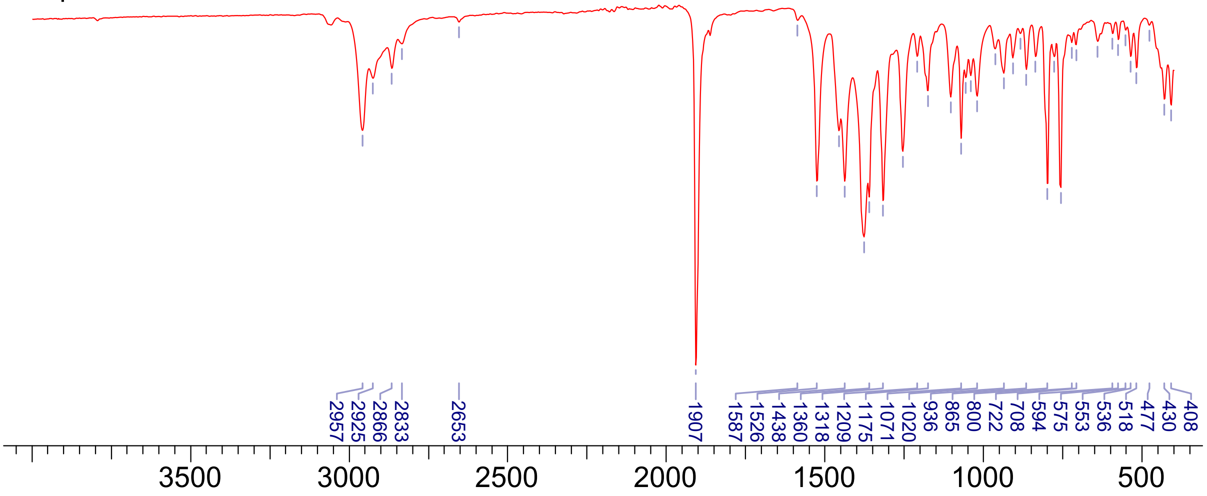

3 Additional spectroscopic details

Figure S11. $^{31}P\{^{1}H\}$ NMR spectra of the conversion of **1** with a large excess of CO_2 in C_6D_6. The ratio of the compounds in the reaction mixture after the addition of CO_2 depends on the amount of CO_2 that is added.

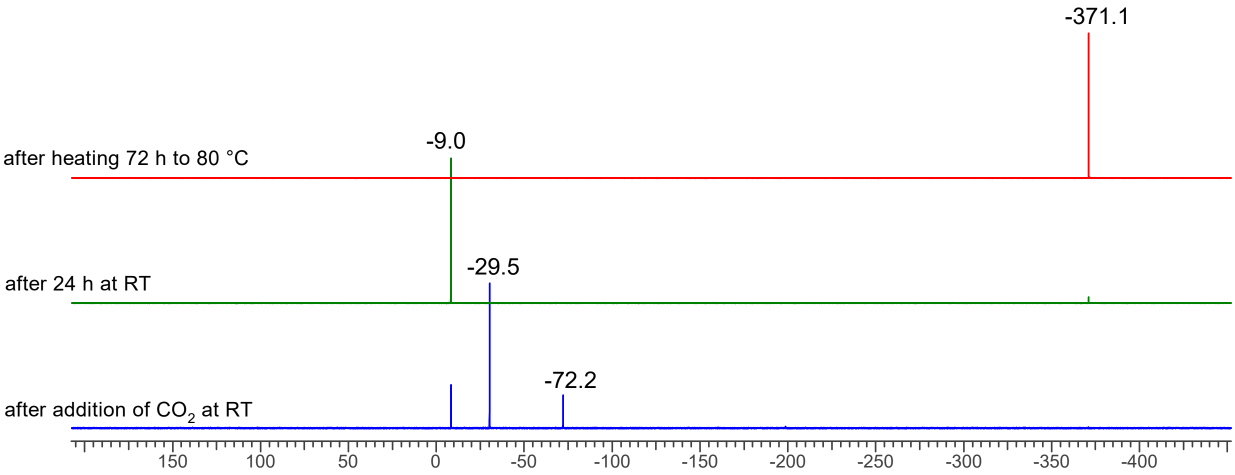

NMR spectroscopic tracing of the conversion of 1 with $^{13}CO_2$

Upon conversion of **1** with small amounts of dry $^{13}CO_2$, the reaction mixture shows directly after addition mainly two signals in its $^{31}P\{^{1}H\}$ NMR spectrum: One singlet at −72.2 ppm (starting material **1**) and one doublet at −29.1 ppm with a $^{1}J(^{31}P\text{-}^{13}C)$ coupling constant of 37.5 Hz, which can be assigned to species **6** (Figure S12). The subsequently collected $^{13}C\{^{1}H\}$ NMR spectrum of the reaction solution shows unreacted $^{13}CO_2$ (singlet, 125.1 ppm) and the signal of ^{13}C atom the P*C*O_2 of species **6** at 179.7 ppm with a $^{1}J(^{31}P\text{-}^{13}C)$ coupling constant of 37.5 Hz (Figure S13). When heating this reaction solution to 80 °C for 72 hours, the formation of compound **8** can be observed. The signal now appears in the ^{31}P NMR spectrum as a doublet with a $^{1}J(^{31}P\text{-}^{13}C)$ coupling constant of 92.9 Hz.

Figure S12. $^{31}P\{^{1}H\}$ NMR spectrum after the addition of $^{13}CO_2$ to a solution of **1** in C_6D_6.

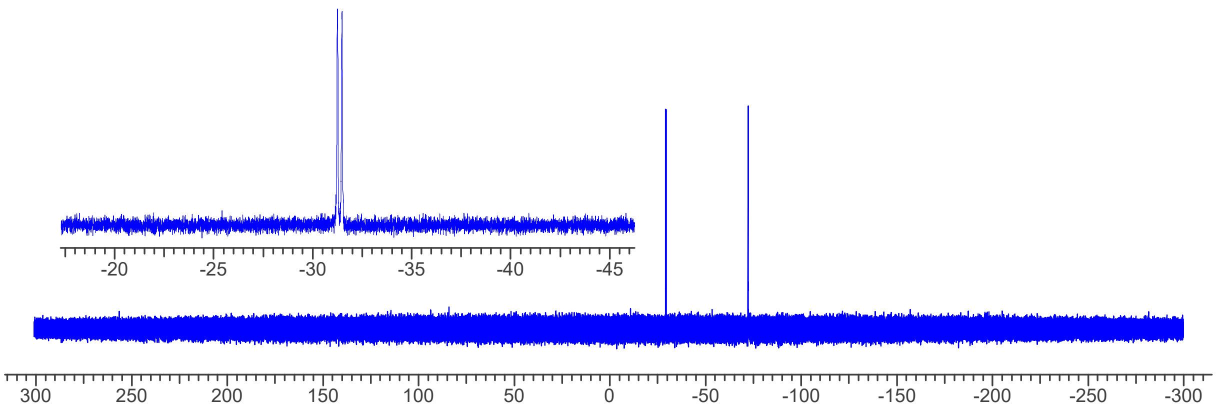

Figure S13. $^{13}C\{^{1}H\}$ NMR spectrum after the addition of $^{13}CO_2$ to a solution of **1** with in C_6D_6.

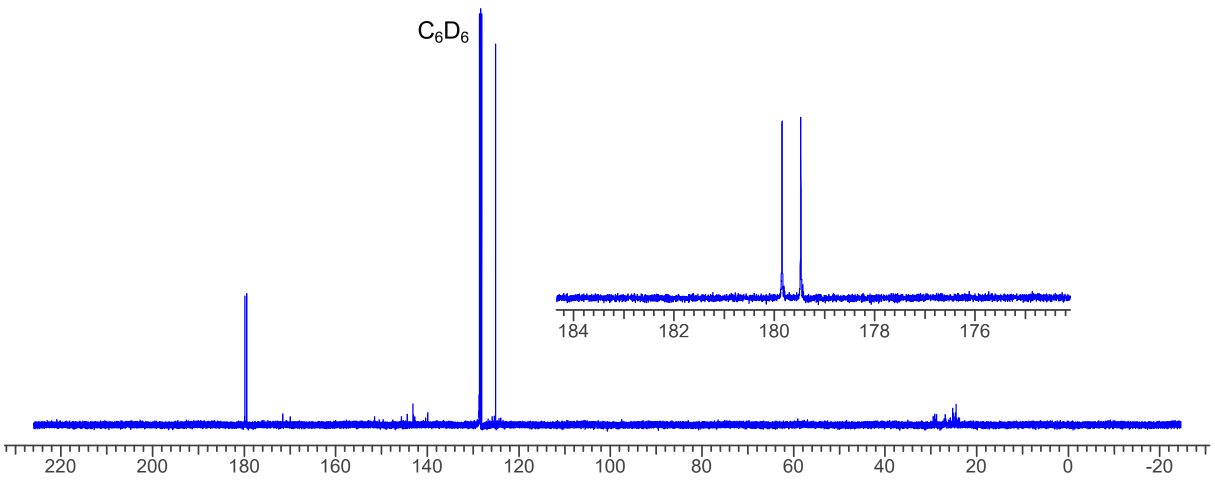

Figure S14. $^{31}P\{^{1}H\}$ NMR spectrum after heating the reaction solution of $^{13}CO_2$ and **1** in C_6D_6 to 80 °C for 72 hours.

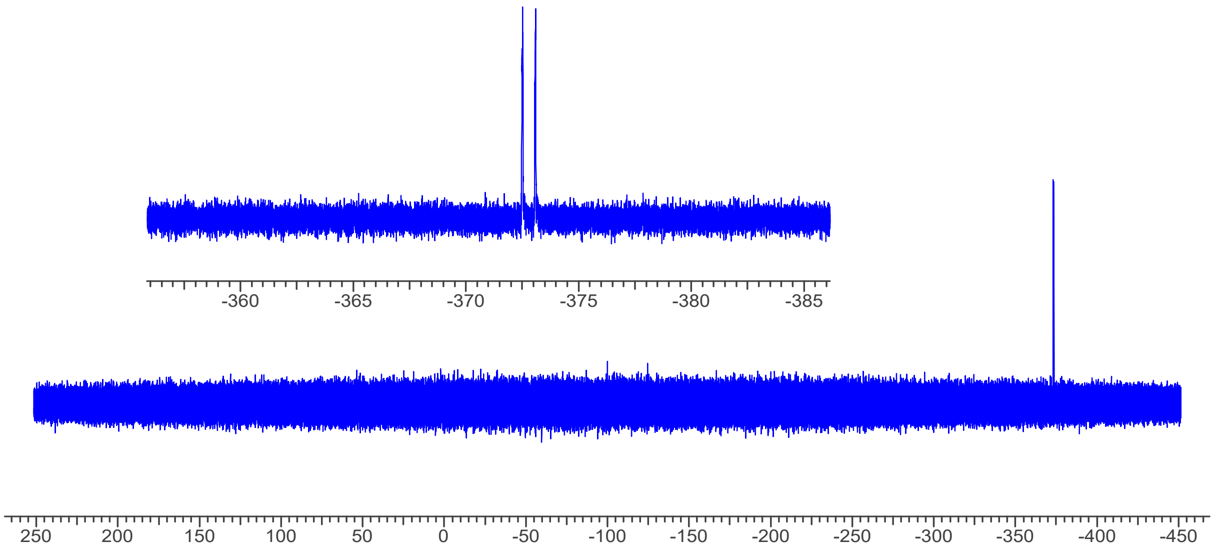

4 Single crystal X-ray diffraction

X-ray structure determination: Single-crystal X-ray diffraction data were collected using either an Oxford Diffraction Supernova dual-source diffractometer equipped with a 135 mm Atlas CCD area detector, a Rigaku XtaLAB Synergy-R diffractometer equipped with a HyPix-Arc 150 detector, or a Bruker APEX-II diffractometer equipped with an APEX II CCD Detector. Crystals were selected under Paratone-N oil, mounted on micro-mount loops and quench-cooled using an Oxford Cryosystems open flow N_2 cooling device. Data were collected using monochromated Cu Kα (λ = 1.54184 Å) or Mo Kα (λ = 0.70173 Å) radiation, and processed using the *CrysAlisPro* or Bruker *SAINT* packages, including unit cell parameter refinement and inter-frame scaling.[83,84] Equivalent reflections were merged, and diffraction patterns processed with the operating suites of the respective instruments. Structures were subsequently solved using direct methods and refined on F^2 using the SHELXL package.[85]

Table S2. Selected X-ray data collection/refinement parameters for **1**, **2**·$0.5C_6H_6$ and **3**·C_6H_6.

	**1**	**2**·$0.5C_6H_6$	**3**·C_6H_6
Formula	$C_{55}H_{79}AsGaN_4P$	$C_{62}H_{91}AsGaN_7P$	$C_{74}H_{107}AsGaN_6P$
CCDC	2409889	2409890	2409891
Fw [g mol^{-1}]	971.83	1110.02	1256.26
Crystal system	monoclinic	triclinic	orthorhombic
Space group	$P2_1/c$	P–1	$P2_12_12_1$
a (Å)	21.3086(2)	12.6878(2)	13.4475(2)
b (Å)	13.9591(1)	22.5101(3)	20.8956(3)
c (Å)	20.6685(2)	22.5732(3)	24.4457(3)
α (°)	90	75.984(1)	90
β (°)	118.573(2)	77.148(1)	90
γ (°)	90	87.776(1)	90
V (Å^3)	5399.07(12)	6097.58(15)	6869.08(17)
Z	4	4	4
Radiation, λ (Å)	Cu Kα, 1.54184	Cu Kα, 1.54184	Cu Kα, 1.54184
Temp (K)	150(2)	150(2)	150(2)
ρ_{calc} (g cm^{-3})	1.196	1.209	1.215
μ (mm^{-1})	1.930	1.785	1.638
Reflections collected	138165	155104	27676
Indep. reflections	11271	25265	13450
Parameters	577	1297	774
R(int)	0.0467	0.0361	0.0325
R1/wR2,[a] I ≥ 2σI (%)	2.56/6.58	2.58/6.60	2.86/6.78
R1/wR2,[a] all data (%)	3.01/6.85	3.03/6.90	3.18/6.96
GOF	1.024	1.021	1.033

[a] $R1 = [\Sigma||F_o| - |F_c||]/\Sigma|F_o|$; $wR2 = \{[\Sigma w[(F_o)^2 - (F_c)^2]^2]/[\Sigma w(F_o^2)^2]\}^{1/2}$; $w = [\sigma^2(F_o)^2 + (AP)^2 + BP]^{-1}$, where $P = [(F_o)^2 + 2(F_c)^2]/3$ and the A and B values are 0.0357 and 1.94 for **1**, 0.0355 and 2.14 for **2**·$0.5C_6H_6$, and 0.0339 and 0.20 for **3**·C_6H_6.

Table S3. Selected X-ray data collection/refinement parameters for **4**·0.5C_6H_6·0.5hex, **5**·4C_6H_6 and **7**.

	**4**·0.5C_6H_6·0.5hex	**5**·4C_6H_6	**7**
Formula	$C_{68}H_{93}AsGaN_5OP$	$C_{86}H_{109}AsGaN_4OP$	$C_{57}H_{79}AsGaN_4O_4P$
CCDC	2409892	2409893	2409894
Fw [g mol^{-1}]	1172.08	1390.38	1059.85
Crystal system	monoclinic	monoclinic	orthorhombic
Space group	$P2_1/n$	$P2_1/n$	$Pna2_1$
a (Å)	12.2864(2)	21.6461(1)	16.3651(1)
b (Å)	25.6905(3)	16.5972(1)	18.4812(1)
c (Å)	20.8532(2)	22.5185(1)	18.6521(1)
α (°)	90	90	90
β (°)	92.4730(10)	106.036(1)	90
γ (°)	90	90	90
V (Å^3)	6576.05(15)	7775.30(8)	5641.26(6)
Z	4	4	4
Radiation, λ (Å)	Cu Kα, 1.54184	Cu Kα, 1.54184	Cu Kα, 1.54184
Temp (K)	150(2)	150(2)	150(2)
ρ_{calc} (g cm^{-3})	1.184	1.188	1.248
μ (mm^{-1})	1.685	1.500	1.946
Reflections collected	48216	215120	275999
Indep. reflections	13608	16224	11678
Parameters	710	708	631
R(int)	0.0409	0.0449	0.0548
R1/wR2,[a] I ≥ 2σI (%)	4.19/10.47	4.42/10.97	3.47/9.52
R1/wR2,[a] all data (%)	5.32/11.40	5.32/11.96	3.57/9.58
GOF	1.026	1.039	1.076

[a] $R1 = [\Sigma||F_o| - |F_c||]/\Sigma|F_o|$; $wR2 = \{[\Sigma w[(F_o)^2 - (F_c)^2]^2]/[\Sigma w(F_o^2)^2]\}^{1/2}$; $w = [\sigma^2(F_o)^2 + (AP)^2 + BP]^{-1}$, where $P = [(F_o)^2 + 2(F_c)^2]/3$ and the A and B values are 0.0514 and 4.87 for **4**·0.5C_6H_6·0.5hex, 0.0536 and 5.22 for **5**·4C_6H_6, and 0.0546 and 3.12 for **7**.

Table S4. Selected X-ray data collection/refinement parameters for **8**·hex, **9**·2hex and **10**·3.5pent.

	**8**·hex	**9**·2hex	**10**·3.5pent
Formula	$C_{62}H_{93}AsGaN_4O_2P$	$C_{68}H_{107}GaN_4P_2S_2$	$C_{129.5}H_{200}As_2Ga_2N_8P_2S_4$
CCDC	2409895	2409896	2409897
Fw [g mol^{-1}]	1102.01	1176.35	2348.42
Crystal system	monoclinic	monoclinic	triclinic
Space group	$P2_1/c$	$P2_1$	P–1
a (Å)	13.3562(1)	15.3228(1)	18.1433(4)
b (Å)	24.7151(2)	14.2179(1)	18.6339(4)
c (Å)	18.5920(1)	15.4652(1)	20.1323(5)
α (°)	90	90	77.529(2)
β (°)	91.831(1)	92.946(1)	85.250(2)
γ (°)	90	90	79.331(2)
V (Å^3)	6134.08(8)	3364.77(4)	6524.4(3)
Z	4	2	2
Radiation, λ (Å)	Cu Kα, 1.54184	Cu Kα, 1.54184	Cu Kα, 1.54184
Temp (K)	200(2)	150(2)	150(2)
ρ_{calc} (g cm^{-3})	1.193	1.161	1.195
μ (mm^{-1})	1.780	1.888	2.261
Reflections collected	73862	51668	162857
Indep. reflections	12771	14011	27016
Parameters	658	767	1351
R(int)	0.0319	0.0298	0.0653
R1/wR2,[a] I ≥ 2σI (%)	2.74/6.83	2.50/6.31	4.20/10.54
R1/wR2,[a] all data (%)	3.23/7.21	2.62/6.41	5.93/11.83
GOF	1.029	1.040	1.017

[a] $R1 = [\Sigma||F_o| - |F_c||]/\Sigma|F_o|$; $wR2 = \{[\Sigma w[(F_o)^2 - (F_c)^2]^2]/[\Sigma w(F_o{}^2)^2\}^{1/2}$; $w = [\sigma^2(F_o)^2 + (AP)^2 + BP]^{-1}$, where $P = [(F_o)^2 + 2(F_c)^2]/3$ and the A and B values are 0.0327 and 2.28 for **855%**·hex, 0.0385 and 0.37 for **9**·2hex, and 0.0624 and 3.77 for **10**·3.5pent.

Table S5. Selected X-ray data collection/refinement parameters for **11**·tol.

	**11**·tol
Formula	$C_{63}H_{87}AsGaN_4OPS$
CCDC	2409898
Fw [g mol^{-1}]	1124.03
Crystal system	orthorhombic
Space group	$Pna2_1$
a (Å)	26.5128(16)
b (Å)	13.4752(7)
c (Å)	33.7623(19)
α (°)	90
β (°)	90
γ (°)	90
V (Å^3)	12062.1(12)
Z	8
Radiation, λ (Å)	Mo Kα, 0.71073
Temp (K)	175(2)
ρ_{calc} (g cm^{-3})	1.238
μ (mm^{-1})	1.104
Reflections collected	626527
Indep. reflections	29951
Parameters	1329
R(int)	0.0503
R1/wR2,[a] I ≥ 2σI (%)	2.63/6.91
R1/wR2,[a] all data (%)	3.05/7.26
GOF	1.067

[a] $R1 = [\Sigma||F_o| - |F_c||]/\Sigma|F_o|$; $wR2 = \{[\Sigma w[(F_o)^2 - (F_c)^2]^2]/[\Sigma w(F_o^2)^2]\}^{1/2}$; $w = [\sigma^2(F_o)^2 + (AP)^2 + BP]^{-1}$, where $P = [(F_o)^2 + 2(F_c)^2]/3$ and the A and B values are 0.0404 and 4.05 for **11**·tol.

5 Additional computational details

Please note that all computations were carried out for single, isolated molecules in the gas phase (ideal gas approximation). There may well be significant differences between gas phase and condensed phase/solution. Where possible, solid state structures were used as a starting point for geometry optimizations. For the optimized .xyz data, see additional supporting file.

GAUSSIAN calculations

Electronic structure computations[63a] for the NBO analysis (see Figure S15) were carried out using Gaussian 09[67]. NBO analyses were made using NBO 6.0.[64-66] To optimize the structure $\mathbf{1}_{\mathbf{DFT}}$ the pure DFT functional PBE[68-70] and the def2-TZVP[71] basis set were used in combination with dispersion correction D3(BJ)[72,73] (notation: PBE-D3/def2TZVP). The structure $\mathbf{1}_{\mathbf{DFT}}$ was fully optimized, and a frequency analysis was performed to confirm the structure as a minimum. Computational data for species $\mathbf{A}_{\mathbf{DFT}}$ and $\mathbf{B}_{\mathbf{DFT}}$ can be found in a previously published study.[29]

Figure S15. Comparison of Kohn-Sham-Orbitals of $\mathbf{B}_{\mathbf{DFT}}$ (left)[29] and $\mathbf{1}_{\mathbf{DFT}}$ (right).

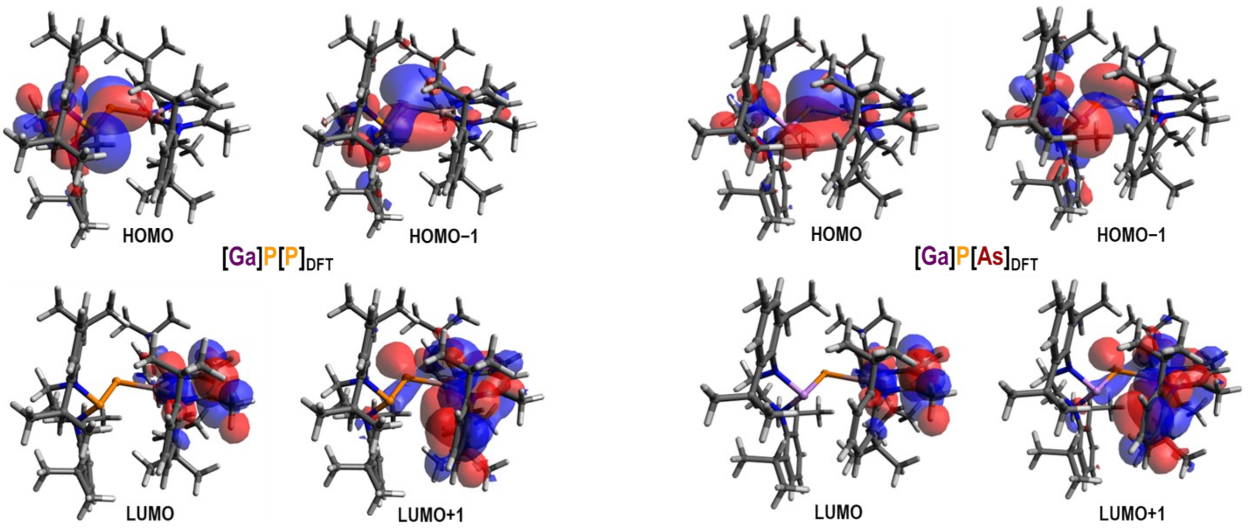

NBO analysis of compound 1_{DFT}

Natural Charges **Ga1** 1.32449 **P1** −0.85707 **As1** 1.03477

Wiberg bond index Ga–P1 1.4548

Wiberg bond index P1–As1 1.0335

BD P1–As1 occupancy 1.95140

55.51% P1 s (13.17%) p (85.59%) d (1.20%) f (0.03%)

43.49% As1 s (14.11%) p (84.91%) d (0.95%) f (0.02%)

BD(1) P1–Ga1 occupancy 1.90707

65.61% P1 s (17.04%) p (81.75%) d (1.20%) f (0.01%)

34.39% Ga1 s (53.09%) p (46.51%) d (0.25%) f (0.15%)

BD(2) P1–Ga1 occupancy 1.87313%

82.66% P1 s (0.07%) p (99.17%) d (0.73%) f (0.03%)

17.34% Ga1 s (0.23%) p (99.09%) d (0.58%) f (0.09%)

LP As1 occupancy 1.95893

s (68.94%) p (31.04%) d (0.02%) f (0.00%)

Summary of calculated data (GAUSSIAN)

Table S6: Summary of calculated data (PBE-D3/def2-TZVP) rounded to the fourth decimal place. All energies in atomic units.

Compound	PG	NIMAG	E_{tot}	U_0	U_{298}	H_{298}	G_{298}
AsPGa	C1	0	−6862.1871	−6860.9555	−6860.8856	−6860.8847	−6861.0596

ORCA calculations

DFT structure optimizations were performed using ORCA 5.0.4[75] and employed the exchange-correlation functional PBE[68-70] in conjunction with Grimme's dispersion correction D3(BJ)[72,73] and the def2-TZVP[71] basis set (notation PBE-D3/def2-TZVP). The resolution-of-identity (RI) approximation was applied, using Weigend's accurate Coulomb-fitting basis set (W06).[63b] All structures were fully optimized and confirmed as minima or transition states by analytic frequency analyses.

Reaction paths were investigated using the nudged elastic band (NEB) method[76-79] implemented in ORCA at the PBE-D3/def2-TZVP level of theory. Subsequently, all relevant transition states were optimized at the PBE-D3/def2-TZVP level of theory as outlined above and confirmed as minima or transitions states by frequency analyses.

As starting points for the NEB scans, the geometry-optimized van-der-Waals adducts were chosen, as products the optimized corresponding (2+2) or (2+3) adducts, respectively. The respective highest energy image (HEI) structure revealed by each NEB scan was geometry optimized and verified as a transition state by frequency analysis. For the (3+2) addition of **1** and COS, only one possible minimum structure was found (coordination of S to P atom and O to Ga atom, **(3+2)_OSC**).

The carbene intermediates were verified to be singlet carbenes (**(3+2)_CO_2_S** and **(3+2)_CS_2_S**) with a closed-shell singlet ground state. The triplet states (**(3+2)_CO_2_T** and **(3+2)_CS_2_T**) are considerably higher in energy (Table S8).

To analyze the dimerization reaction of **(3+2)_CS_2_S** yielding **10**, we used a truncated model system due to the very large size of compound **10**. In the model system, the Dipp substituents were substituted with Ph substituents, indicated as **1_Ph**, **(3+2)_CS_2_Ph** and **10_Ph**. The calculations indicate that the dimerization process from **(3+2)_CS_2_Ph** yielding formally 0.5 equivalents **10_Ph** is a highly exergonic process (−115.3 kJ/mol). Although the formation of the model system **(3+2)_CS_2_Ph** was computed to be overall slightly exergonic (−23.5 kJ/mol, Table S7) unlike the formation of the carbene intermediate with Dipp substituents **(3+2)_CS_2** (+12.2 kJ/mol, Table S7) the overall energetic trend for the exergonic formation of the dimerization product becomes clearly visible from these model calculations.

Table S7: Free reaction energies $\Delta G°_{298}$ in kJ/mol (PBE-D3/def2-TZVP) for the formation of the indicated compounds starting from **1** + CX_2 (X = O, S).

**Reaction product**	**1_CO_2_vdW**	**TS_(2+2)_CO_2**	**6_{DFT}**	**(3+2)_CO_2_S**	**7_{DFT}**
**d*G*°$_{298}$** [kJ/mol]	37.7	75.2	−6.6	130.6	−9.4
**Reaction product**	**1_CS_2_vdW**	**TS_(2+2)_CS_2**	**6_CS_2_$_{DFT}$**	**TS_(3+2)_CS_2**	**(3+2)_CS_2_S**
**d*G*°$_{298}$** [kJ/mol]	30.8	80.5	−91.9	54.3	12.2
**Reaction product**	**6_OCS_$_{DFT}$**	**6_SCO_$_{DFT}$**	**(3+2)_OSC**		
**d*G*°$_{298}$** [kJ/mol]	−15.9	−64.5	65.5		
**Reaction product**	**(3+2)_CS_2_Ph**	**½ 10_Ph**			
**d*G*°$_{298}$** [kJ/mol]	−23.5*	−115.3*			

6_OCS_$_{DFT}$ coordination mode: GaOPC heterocycle; **6_SCO_$_{DFT}$** coordination mode: GaSPC heterocycle; **(3+2)_OSC**: GaO and PS bonds. *In the model systems **(3+2)_CS_2_Ph** and **10_Ph**, the Dipp groups are substituted with Ph groups, the calculated energies of **(3+2)_CS_2_Ph** and **10_Ph** are referring to **1_Ph** as starting material.

Figure S16. Schematic view of frontier orbitals of **1** (HOMO) and CO_2/CS_2 (only π_x orbitals displayed, HOMO−1, HOMO and LUMO).

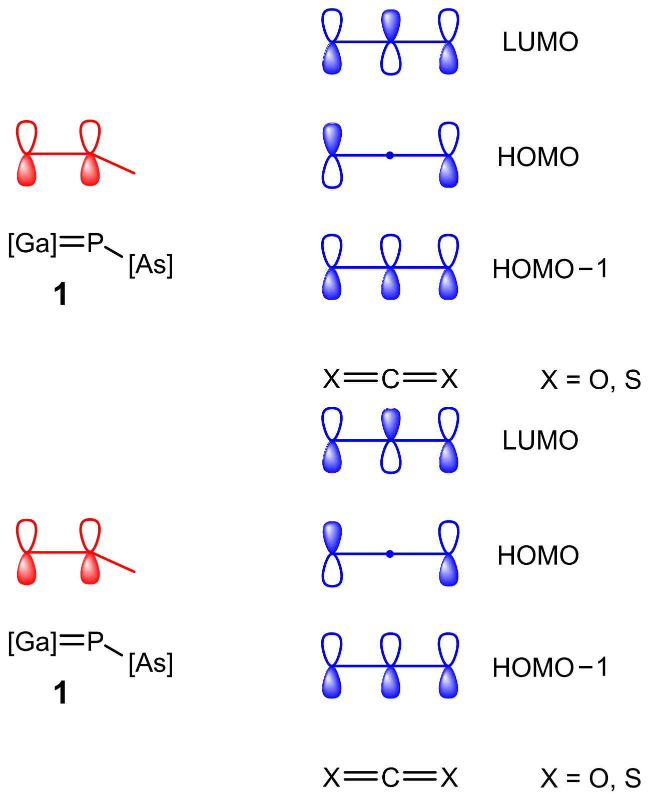

Figure S17. Schematic view of the energy diagram of the (2+2) or (2+3) addition reactions of **1** and CO_2 or CS_2, respectively at the PBE-D3/def2-TZVP level of theory. *For computational reasons, the dimerization energy $\Delta G°_{dim}$ formally yielding 0.5 eq. **10** was calculated for a truncated model system **10_Ph**.

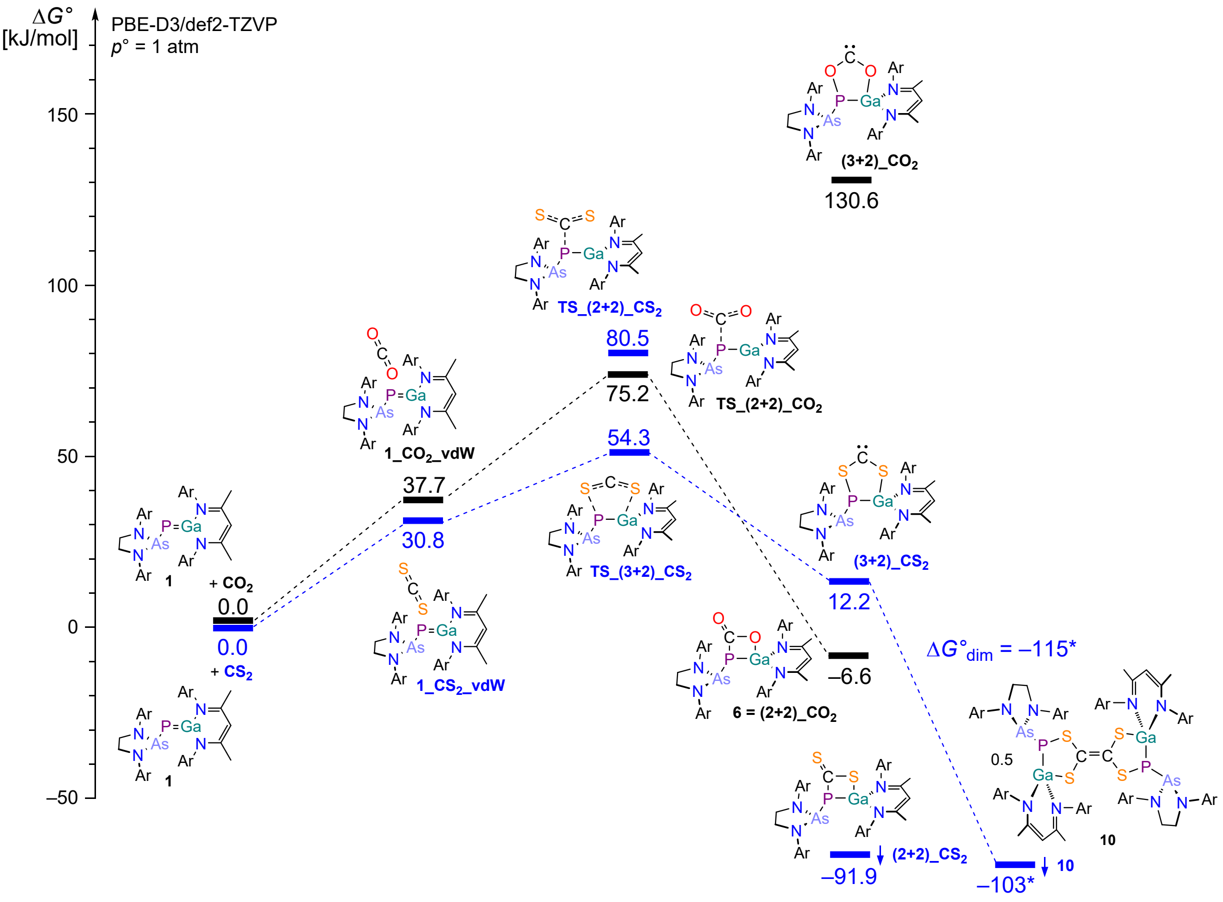

Summary of calculated data (ORCA)

Table S8: Summary of calculated data (PBE-D3/def2-TZVP) rounded to the fourth decimal place. All energies in atomic units.

Compound	PG	NIMAG	E_{tot}	U_0	U_{298}	H_{298}	G_{298}
**1_{DFT}**	C_1	0	−6861.7569	−6860.5662	−6860.4936	−6860.4927	−6860.6599
**CO_2**	$D_{\infty h}$	0	−188.4803	−188.4704	−188.4674	−188.4666	−188.4848
**1_CO_2_vdW**	C_1	0	−7050.2351	−7049.0323	−7048.9555	−7048.9545	−7049.1303
**TS_(2+2)_CO_2**	C_1	1	−7050.2228	−7049.0202	−7048.9450	−7048.9440	−7049.1160
**6_{DFT}**	C_2	0	−7050.2550	−7049.0513	−7048.9758	−7048.9749	−7049.1472
**(3+2)_CO_2_S**	C_1	0	−7050.2011	−7048.9988	−7048.9231	−7048.9221	−7049.0949
**(3+2)_CO_2_T**	C_1	0	−7050.1185				
**7_{DFT}**	C_1	0	−7238.7488	−7237.5326	−7237.4539	−7237.4530	−7237.6331
**CS_2**	$D_{\infty h}$	0	−834.1570	−834.1511	−834.1478	−834.1469	−834.1687
**1_CS_2_vdW**	C_s	0	−7695.9181	−7694.7192	−7694.6422	−7694.6413	−7694.8168
**TS_(2+2)_CS_2**	C_1	1	−7695.8973	−7694.7002	−7694.6233	−7694.6224	−7694.7979
**6_CS_2_DFT**	C_1	0	−7695.9655	−7694.7665	−7694.6899	−7694.6890	−7694.8635
**TS_(3+2)_CS_2**	C_1	1	−7695.9073	−7694.7098	−7694.6330	−7694.6321	−7694.8079
**(3+2)_CS_2_S**	C_1	0	−7695.9251	−7694.7266	−7694.6499	−7694.6490	−7694.8239
**(3+2)_CS_2_T**	C_1	0	−7695.8571				
**COS**	$C_{\infty v}$	0	−511.3197	−511.3118	−511.3087	−511.3078	−511.3280
**6_OCS_{DFT}**	C_1	0	−7373.0974	−7371.8965	−7371.8204	−7371.8195	−7371.9939
**6_SCO_{DFT}**	C_1	0	−7373.1160	−7371.9149	−7371.8386	−7371.8377	−7372.0124
**(3+2)_OSC**	C_1	0	−7373.0663	−7371.8664	−7371.7901	−7371.7891	−7371.9629
**1_Ph**	C_1	0	−5919.1720	−5918.6366	−5918.5994	−5918.5985	−5918.6998
**(3+2)_CS_2_Ph**	C_1	0	−6753.3543	−6752.8108	−6752.7696	−6752.7686	−6752.8775
**10_Ph**	C_1	0	−13506.8256	−13505.7356	−13505.6518	−13505.6508	−13505.8427

6_OCS_{DFT} coordination mode: GaOPC heterocycle; **6_SCO_{DFT}** coordination mode: GaSPC heterocycle; **(3+2)_OSC**: GaO and PS bonds; **(3+2)_CO_2_S** and **(3+2)_CS_2_S** closed-shell singlet states, **(3+2)_CO_2_T** and **(3+2)_CS_2_T** triplet states.

6 References

(Numeration of references in ESI as cited in the manuscript)

[29] L. S. Szych, L. Denker, J. Feld and J. M. Goicoechea, *Chem. Eur. J.* **2024**, e202401326.

[30] D. W. N. Wilson, J. Feld and J. M. Goicoechea, *Angew. Chem. Int. Ed.* **2020**, *59*, 20914–20918.

[63] (a) J. Bresien, *SLURM interface for ORCA and Gaussian*, University of Rostock, **2020**; (b) F. Weigend, *Phys. Chem. Chem. Phys.* **2006**, *8*, 1057–1065.

[64] (a) F. E. D. Glendening, J. K. Badenhoop, A. E. Reed, J. E. Carpenter, J. A. Bohmann, C. M. Morales, C. R. Landis and F. Weinhold, **NBO 6.0**. Theoretical Chemistry Institute, University of Wisconsin: Madison 2013. (b) J. E. Carpenter and F. Weinhold, *J. Mol. Struct.: THEOCHEM* **1988**, *169*, 41–62.

[65] F. Weinhold and C. R. Landis, in *Valency and Bonding. A Natural Bond Orbital Donor-Acceptor Perspective*, Cambridge University Press, **2005**.

[66] F. Weinhold and J. Carpenter, in *The Structure of Small Molecules and Ions*, ed. R. Naaman, Z. Vager, Springer US, Boston, MA, **1988**, pp. 227–236.

[67] M. J. Frisch, G. W. Trucks, H. B. Schlegel, G. E. Scuseria, M. A. Robb, J. R. Cheeseman, G. Scalmani, V. Barone, B. Mennucci, G. A. Petersson, H. Nakatsuji, M. Caricato, X. Li, H. P. Hratchian, A. F. Izmaylov, J. Bloino, G. Zheng, J. L. Sonnenberg, M. Hada, M. Ehara, K. Toyota, R. Fukuda, J. Hasegawa, M. Ishida, T. Nakajima, Y. Honda, O. Kitao, H. Nakai, T. Vreven, J. A. Montgomery Jr., J. E. Peralta, F. Ogliaro, M. Bearpark, J. J. Heyd, E. Brothers, K. N. Kudin, V. N. Staroverov, T. Keith, R. Kobayashi, J. Normand, K. Raghavachari, A. Rendell, J. C. Burant, S. S. Iyengar, J. Tomasi, M. Cossi, N. Rega, J. M. Millam, M. Klene, J. E. Knox, J. B. Cross, V. Bakken, C. Adamo, J. Jaramillo, R. Gomperts, R. E. Stratmann, O. Yazyev, A. J. Austin, R. Cammi, C. Pomelli, J. W. Ochterski, R. L. Martin, K. Morokuma, V. G. Zakrzewski, G. A. Voth, P. Salvador, J. J. Dannenberg, S. Dapprich, A. D. Daniels, O. Farkas, J. B. Foresman, J. V. Ortiz, J. Cioslowski and D. J. Fox, **Gaussian 09**, *Revision E.01*, Gaussian, Inc., Wallingford CT, **2013**.

[68] J. P. Perdew, K. Burke and M. Ernzerhof, *Phys. Rev. Lett.* **1996**, *77*, 3865–3868.

[69] J. P. Perdew, K. Burke and M. Ernzerhof, *Phys. Rev. Lett.* **1997**, *78*, 1396.

[70] C. Adamo and V. Barone, *J. Chem. Phys*. **1999**, *110*, 6158–6170.

[71] F. Weigend and R. Ahlrichs, *Phys. Chem. Chem. Phys.* **2005**, *7*, 3297.

[72] S. Grimme, J. Antony, S. Ehrlich and H. Krieg, *J. Chem. Phys.* **2010**, *132*, 154104.

[73] S. Grimme, S. Ehrlich and L. Goerigk, *J. Comput. Chem.* **2011**, *32*, 1456–1465.

[75] F. Neese, *WIREs Comput. Mol. Sci.* **2022**, *12*, 1–15.

[76] (a) G. Mills, H. Jónsson and G. K. Schenter, *Surf. Sci.*, 1995, **324**, 305–337; (b) H. Jónsson, G. Mills and K. W. Jacobsen, in *Classical and Quantum Dynamics in Condensed Phase Simulations*, WORLD SCIENTIFIC, **1998**, pp. 385–404.

[77] G. Henkelman and H. Jónsson, *J. Chem. Phys.* **2000**, *113*, 9978–9985.

[78] G. Henkelman, B. P. Uberuaga and H. Jónsson, *J. Chem. Phys.* **2000**, *113*, 9901–9904.

[79] E. Maras, O. Trushin, A. Stukowski, T. Ala-Nissila and H. Jónsson, *Comput. Phys. Commun.* **2016**, *205*, 13–21.

[80] O. Kysliak, H. Görls and R. Kretschmer, *Dalton Trans.* **2020**, *49*, 6377–6383.

[81] W. L. F. Armarego and C. L. L. Chai, *Purification of Laboratory Chemicals*; Elsevier, **2013**.

[82] M. Swetha, P. Venkata Ramana and S. G. Shirodkar, *Org. Prep. Proced. Int.* **2011**, *43*, 348–353.

[83] *CrysAlisPro*, Agilent Technologies, Version 1.171.42.72a.

[84] *SAINT V8.40A* (**2020**), Bruker AXS, Madison, WI.

[85] (a) G. M. Sheldrick in SHELXL97, Programs for Crystal Structure Analysis (Release 97-2), Institut für Anorganische Chemie der Universität, Tammanstrasse 4, D-3400 Göttingen, Germany, **1998**; (b) G. M. Sheldrick, *Acta Crystallogr. Sect. A* **1990**, *46*, 467−473; (c) G. M. Sheldrick, *Acta Crystallogr. Sect. A* **2008**, *64*, 112−122.
